# Synthesis and Biological Evaluation of *β*-Phenylalanine Derivatives Containing Sulphonamide and Azole Moieties as Antiproliferative Candidates in Lung Cancer Models

**DOI:** 10.3390/molecules30153303

**Published:** 2025-08-07

**Authors:** Vytautas Mickevičius, Kazimieras Anusevičius, Birutė Sapijanskaitė-Banevič, Ilona Jonuškienė, Linas Kapočius, Birutė Grybaitė, Ramunė Grigalevičiūtė, Povilas Kavaliauskas

**Affiliations:** 1 Department of Organic Chemistry, Kaunas University of Technology, Radvilenu˛ Rd. 19, LT-50254 Kaunas, Lithuania; vytautas.mickevicius@ktu.lt (V.M.); povilas.kavaliauskas@som.umaryland.edu (P.K.); 2 Biological Research Centre, Lithuanian University of Health Sciences, Tilžės g. 18, LT-47181 Kaunas, Lithuania; 3 Department of Animal Nutrition, Lithuanian University of Health Sciences, Tilžės g. 18, LT-47181 Kaunas, Lithuania; 4 Department of Microbiology and Immunology, University of Maryland School of Medicine, Baltimore, MD 21201, USA; 5 Institute of Infectious Diseases and Pathogenic Microbiology, Birštono Str. 38A, LT-59116 Prienai, Lithuania

**Keywords:** oxadiazole, triazole, 1,2,4-triazolo [3,4-*b*][1,3,4]thiadiazine, sulphanilamide, β-phenylalanine, azole, H69AR cells, anticancer agents

## Abstract

In this study, a series of novel *β*-phenylalanine derivatives were synthesised and evaluated for their anticancer activity. The 3-(4-methylbenzene-1-sulfonamido)-3-phenylpropanoic acid (**2**) was prepared using *β*-phenylalanine as a core scaffold. The *β*-amino acid derivative **2** was converted to the corresponding hydrazide **4**, which enabled the development of structurally diverse heterocyclic derivatives including pyrrole **5**, pyrazole **6**, thiadiazole **8**, oxadiazole **11**, triazoles **9** and **12** with Schiff base analogues **13** and series1,2,4-triazolo [3,4-*b*][1,3,4]thiadiazines **14**. These modifications were designed to enhance chemical stability, solubility, and biological activity. All compounds were initially screened for cytotoxicity against the A549 human lung adenocarcinoma cell line, identifying *N*-[3-(3,5-dimethyl-1*H*-pyrazol-1-yl)-3-oxo-1-phenylpropyl]-4-methylbenzenesulfonamide (**5**) and (*E*)-*N*-{2-[4-[(4-chlorobenzylidene)amino]-5-thioxo-4,5-dihydro-1*H*-1,2,4-triazol-3-yl]-1-phenylethyl}-4-methylbenzenesulfonamide (**13b**) as the most active. The two lead candidates were further evaluated in H69 and H69AR small cell lung cancer lines to assess activity in drug-sensitive and multidrug-resistant models. Schiff base **13b** containing a 4-chlorophenyl moiety, retained potent antiproliferative activity in both H69 and H69AR cells, comparable to cisplatin, while compound **5** lost efficacy in the resistant phenotype. These findings suggest Schiff base derivative **13b** may overcome drug resistance mechanisms, a limitation commonly encountered with standard chemotherapeutics such as doxorubicin. These results demonstrate the potential role of *β*-phenylalanine derivatives, azole-containing sulphonamides, as promising scaffolds for the development of novel anticancer agents, particularly in the context of lung cancer and drug-resistant tumours.

## 1. Introduction

Numerous biochemical regulatory pathways and molecular targets have been identified as promising strategies in the development of effective anticancer therapies, with particular attention given to small molecules capable of modulating these pathways [1]. One such pathway involves sphingosine-1-phosphate (S1P), a bioactive sphingolipid metabolite that, through interactions with extracellular chaperones, regulates a wide range of physiological and pathological processes, including cell proliferation, migration, survival, and angiogenesis [2]. Among the five known S1P receptors, sphingosine-1-phosphate receptor 1 (S1PR1) has emerged as a key mediator of these effects. Notably, the S1PR1 signalling is recognised as a therapeutically actionable target, with small-molecule modulators of S1PR1 showing potential to disrupt tumour-promoting signalling networks and overcome drug resistance in various cancers [3,4,5]. Against this background, *α*-phenylalanine derivatives containing azole and sulphonamide moieties have been developed as small-molecule antagonists of S1PR1, demonstrating significant biological activity [6]. However, their limited metabolic stability has restricted their development as drug candidates.

Another potential target for small-molecule compounds is CYP26A1 [7], which is considered a promising therapeutic candidate due to its critical role in retinoid metabolism and its association with cancer cell resistance. In study [8], a compound with an α-phenylalanine scaffold was identified as a potential CYP26A1 inhibitor in an in silico study. However, the metabolic stability of the compound in vitro was insufficient. Considering this limitation, *β*-phenylalanine was proposed as an alternative pharmacophore for *a*-phenylalanine. Novel *β*-phenylalanine derivatives were rationally designed to form coordination bonds with the haem group of CYP26A1, thereby enhancing their interaction with the target and improving their therapeutic potential.

Aminopeptidase N (APN/CD13) is a membrane-bound enzyme that is frequently overexpressed in tumour cells and plays a critical role in cancer invasion, metastasis, and angiogenesis [9]. By selecting β-phenylalanine as a non-proteogenic amino acid and a metabolically stable pharmacophoric scaffold, a series of compounds was synthesised with potential therapeutic activity against aminopeptidase N (APN) [10]. The resulting derivatives exhibited promising biological activity, supporting their candidacy for targeted anticancer therapy.

In another study [11], a series of sulphonamide derivatives containing a *β*-phenylalanine scaffold was synthesised and evaluated for their inhibitory activity against eukaryotic elongation factor 2 kinase (eEF2K), as well as for their cytotoxic effects. eEF2K, an atypical Ca^2+^/calmodulin-dependent Ser/Thr kinase, is frequently overexpressed or hyperactivated in various cancer types and is, therefore, considered a compelling target for therapeutic intervention. The study [11] revealed that several compounds exhibited significant biological activity, supporting their potential as lead candidates for the development of eEF2K-targeted anticancer agents.

A review of the literature reveals that, in the development of effective small-molecule agents for targeted cancer therapy, *β*-phenylalanine scaffolds serve as metabolically stable cores. The design rationale was further strengthened by the inclusion of sulphonamide and azole moieties, which are known for their improved target binding and pharmacokinetic properties [12,13,14]. This structural combination allows the design of compounds capable of modulating key cancer-related targets such as S1PR1, CYP26A1, APN/CD13, and eEF2K, supporting the structure–activity relationship approach. Consequently, derivatives incorporating *β*-phenylalanine, sulphonamides, and azoles are being investigated as potential candidates for targeted anticancer therapy.

## 2. Results and Discussions

### 2.1. Chemistry

**Scheme 1:**
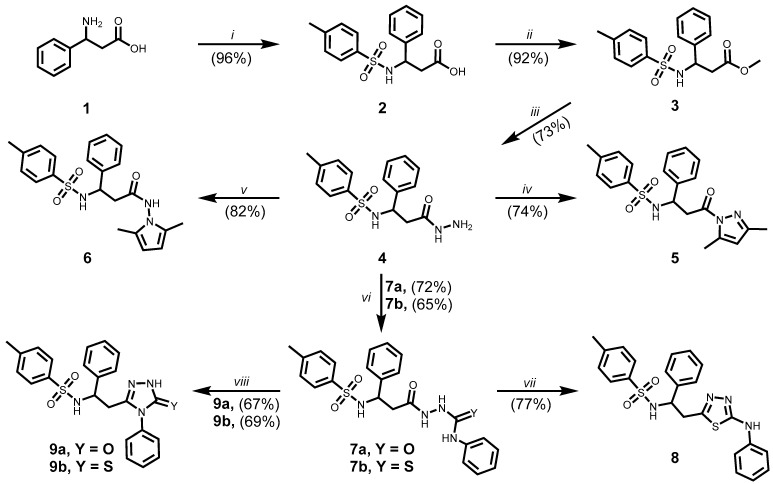
Synthesis of compounds **2**–**9**. (**i**) 4-TsCl, 15% Na_2_CO_3(aq)_, Et_2_O, r.t., 24 h, 20% HCl, pH~3; (**ii**) CH_3_OH, H_2_SO_4_, reflux, 8 h; (**iii**) NH_2_NH_2_ ^.^ H_2_O, 2-PrOH, reflux, 24 h; (**iv**) 2,4-pentanedione, 2-PrOH, AcOH, reflux, 2 h; (**v**) 2,5-hexanedione, 1,4-dioxane, AcOH, reflux 2 h; (**vi**) phenyl isocyanate or phenyl isothiocyanate, MeOH, reflux, 4 h; (**vii**) 80% H_2_SO_4_, 0 °C to r.t., 24 h, 5% NH_4_OH, pH~7; (**viii**) 4% NaOH_(aq)_, reflux, 4 h, 20% HCl, pH~6.

In this work, all compounds **2**–**14** were synthesised starting from (±)-*β*-phenylalanine (**1**), which was used as a racemic mixture. The synthesis of compounds **2**–**9** is presented in Scheme 1.

The synthesis of 3-(4-methylbenzenesulfonamido)-3-phenylpropanoic acid (**2**) was carried out in a two-phase reaction system to optimise the interaction of the reagents and prevent premature hydrolysis of 4-methylbenzenesulfonyl chloride. Initially, (±)-*β*-phenylalanine (**1**) was dissolved in a 15% aqueous sodium carbonate solution, while 4-methylbenzenesulfonyl chloride was dissolved separately in diethyl ether. The ether solution was added dropwise to the alkaline aqueous phase under ice bath cooling to minimise side reactions and ensure controlled reagent addition. Following the completion of the addition, the reaction mixture was stirred at ambient temperature. 3-(4-Methylbenzene-1-sulfonamido)-3-phenylpropanoic acid (**2**) was precipitated by acidifying the aqueous layer to pH 2 with hydrochloric acid. A racemic mixture of compound **1** was used for the synthesis of compound **2**. Since the sp^3^-hybridised configuration at the chiral centre appears to be maintained during the reaction, it can be assumed that compound **2**, along with the subsequently synthesised derivatives **3**–**14** described in this study, were also obtained as racemic mixtures [15].

The ester **3** was synthesised via the classical Fischer esterification method. Methyl 3-(4-methylbenzene-1-sulfonamido)-3-phenylpropanoate (**3**) was synthesised from compound **2** by refluxing in methanol in the presence of concentrated sulphuric acid as a catalyst. Subsequent hydrazinolysis of ester **3** in propan-2-ol afforded *N*-[3-hydrazinyl-3-oxo-1-phenylpropyl]-4-methylbenzene-1-sulfonamide (**4**). All synthesised compounds were purified by recrystallisation from an appropriate solvent or solvent mixture as reported in Section 3.1. The introduction of additional polar functional groups, including hydrazine and amide moieties, increased the number of labile protons and potential hydrogen bonding sites within molecule **4**. In the ^1^H NMR spectrum of compound **4**, broad singlets were observed at 4.05 ppm and 7.94 ppm, corresponding to the NH_2_ and NH protons, respectively (Supplementary Materials, Figure S5). The signal broadening observed is most likely the result of intermolecular hydrogen bonding and specific solvent interactions—both of which appear to affect proton mobility and alter relaxation dynamics. These results indicate that such molecular interactions play a key role in modulating the exchange behaviour of labile protons, thereby contributing to the broadening of NMR signals [16]. The NMR and FTIR spectra of hydrazide **4** are supported in Supplementary Materials (Figures S5, S6 and S47).

The cyclocondensation of hydrazide **4** was carried out using either 2,5-pentanedione or 2,5-hexanedione in the presence of acetic acid as a catalyst. These reactions afforded heterocyclic derivatives containing pyrazole **5** and pyrrole **6** moieties, respectively. Formation of the heterocyclic cores was confirmed by the presence of characteristic signals in the ^1^H NMR spectra, consistent with those observed for structurally related compounds in earlier studies and other authors’ works [17,18,19]. The data of the ^1^H and ^13^C NMR and FTIR spectra of synthesised compounds pyrazole **5** and pyrrole **6** are included in the Supplementary Materials (Figures S7–S10, S48 and S49).

Compounds **2**–**5** have been previously reported in the literature [20,21,22,23,24], as referenced in Section 3.1 Chemistry in relation to their melting points. Moreover, their anticancer activity has not been evaluated against the specific cell lines examined in this study.

Hydrazide **4** was treated with phenyl isocyanate and phenyl isothiocyanate in methanol to yield the corresponding semicarbazide **7a** and thiosemicarbazide **7b** derivatives, respectively. The ^1^H and ^13^C NMR and FTIR spectral data for the synthesised compounds **7a** and **b** are presented in the Supplementary Materials (Figures S11–S14, S50 and S51). Subsequently, the cyclocondensation reactions of compounds **7a** and **7b** were carried out under acidic and alkaline conditions. The 5-amino-1,3,4-thiadiazole derivative **8** was subsequently synthesised via intramolecular cyclocondensation of compound **7b** upon treatment with 80% sulphuric acid at room temperature for 24 h. Furthermore, refluxing compounds **7a** or **7b** in 4% aqueous sodium hydroxide afforded the corresponding 1,2,4-triazole derivatives **9a** and **9b**, respectively. In the ^1^H NMR spectrum of compound **9a**, a singlet at 11.84 ppm was observed, which was attributed to the NH proton of the triazole ring. In the corresponding ^13^C NMR spectrum of compound **9a**, a signal at 153.72 ppm was detected, consistent with the presence of a carbonyl carbon (C=O) (Supplementary Materials, Figure S18). The molecular structure, which contains multiple nucleophilic centres, suggests the possibility of tautomeric equilibrium between keto and enol forms [25,26,27]. Based on the NMR data recorded in DMSO-d_6_, the carbonyl tautomer appears to be the predominant form under these conditions. An intense absorption band at 1708 cm^−1^ was observed in the FTIR spectrum of compound **9a**, providing unequivocal evidence that the keto tautomeric form is present in the crystalline state (Supplementary Materials, Figure S53). Similarly, for compound **9b**, the NMR spectra indicate that the thione tautomer is favoured in DMSO-d_6_ [28]. This observation is supported by characteristic chemical shifts and is consistent with previously reported behaviour of structurally related compounds under similar conditions [29]. The absorption band observed at 1306 cm^−1^ in the FTIR spectrum of compound **9b** was assigned to the C=S group, which indicates that the thione tautomeric form is present in the crystalline state (Supplementary Materials, Figure S54).

**Scheme 2:**
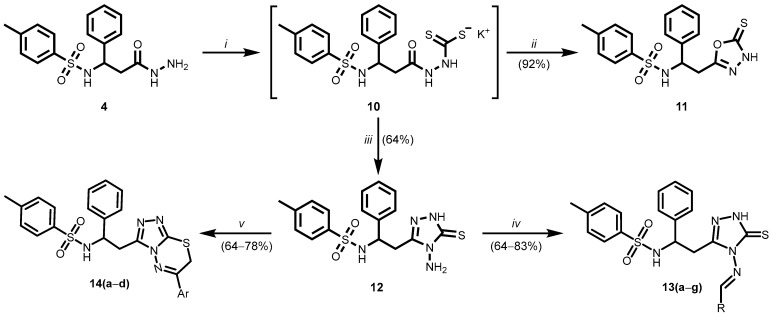
(**13a**) R = C_6_H_5_; (**13b**) R = 4-ClC_6_H_4_; (**13c**) R = 4-BrC_6_H_4_; (**13d**) R = 4-N(CH_3_)_2_C_6_H_4_; (**13e**) R = thiophen-2-yl; (**13f**) R = 3-indolyl; (**13g**) R = *N*-ethyl-9*H*-carbazol-3-yl; (**14a**) Ar = C_6_H_5_; (**14b**) Ar = 4-FC_6_H_4_; (**14c**) Ar = 4-ClC_6_H_4_; (**14d**) Ar = 4-BrC_6_H_4_. (**i**) CS_2_, KOH, butan-1-ol, reflux 48 h; (**ii**) glacial AcOH, reflux, H_2_O; (**iii**) NH_2_NH_2_ ^.^ H_2_O; butan-1-ol, reflux, 72 h, glacial AcOH, reflux, H_2_O; (**iv**) RCHO, 2-PrOH, reflux 8–24 h; (**v**) corresponding 2-bromoacetophenone, 1,4-dioxane, reflux, 8–18 h, 10% AcONa_(aq)_.

Compounds **11**–**14** were synthesised following the synthetic route depicted in Scheme 2.

Potassium dithiocarbamate **10** was synthesised by the method described in the literature [30,31,32]. Typically, the intermediate is not isolated from the reaction mixture and it is used without isolation or further purification. Potassium dithiocarbazate **10** was synthesised by reacting hydrazide **4** with carbon disulphide in an alcoholic solvent in the presence of potassium hydroxide. The 1,3,4-oxadiazole derivative **11** was obtained by acid-catalysed cyclisation of the dithiocarbazate moieties into a 1,3,4-oxadiazole ring [33]. In ^1^H NMR spectrum of compound **11** was observed a broad singlet at 14.25 ppm, which was assigned to the NH proton of the 1,3,4-oxadiazole, while the ^13^C NMR spectrum showed a signal at 177.53 ppm, corresponding to the thiocarbonyl carbon atom (C=S). NMR and FTIR data are supported in the Supplementary Materials (Figures S21, S22 and S55). However, the NMR data recorded in DMSO-d_6_ indicate the predominance of the thione tautomeric form under these conditions [30,34].

Compound **12** was synthesised by the reaction of the intermediate **10** with hydrazine monohydrate, the synthesis was carried out in butan-1-ol at reflux for 72 h. When the reaction was complete, the volatile components were removed under reduced pressure, and the resulting residue was treated with glacial acetic acid to afford the 4-amino-1,2,4-triazole derivative **12**. Butan-1-ol was chosen as the solvent for the reaction due to its higher boiling point, which results in more efficient conversion. However, when the reaction was carried out in methanol under similar conditions, no significant progress was observed. The NMR spectroscopic data of compound **12** support conclusions analogous to those drawn for compound **11**. The data of the ^1^H and ^13^C NMR, FTIR spectra of derivative **12** are included in the Supplementary Materials (Figures S23, S24 and S56). In the ^13^C NMR spectrum, the C=S carbon signal appears at 166 ppm, indicating a slightly more shielded environment compared to compound **11**. Nevertheless, this signal remains consistent with the predominance of the thione tautomeric form in DMSO-d_6_.

A series of Schiff base-type compounds **13a**–**g** was obtained by the condensation of the 4-amino-1,2,4-triazole derivative **12** with various aromatic and heterocyclic aldehydes in the presence of hydrochloric acid as a catalyst. Considering the literature describing structurally analogous moieties [35], it is reasonable to assume that the synthesised Schiff bases **13a**–**g** are the *E* isomer. The presence of a single imine proton signal in the ^1^H NMR spectra, typically appearing as a singlet around 9.5 ppm, supports the formation of a single geometric isomer. In the ^1^H NMR spectrum of compound **13a**, a singlet at 9.53 ppm was observed, corresponding to the imine proton, indicating the formation of a single structural isomer. Similar spectral features were observed for the remaining Schiff bases **13b**–**g**, supporting the formation of structurally analogous products [36]. The data of the ^1^H and ^13^C NMR, FTIR spectra of derivatives **13a**–**g** are included in the Supplementary Materials (Figures S25–S38, and S57–S63). In order to further substantiate the proposed structure of compound **13b**, high resolution mass spectrometry (HRMS) analysis was performed. The corresponding spectrum is presented in the Supplementary Materials (Figure S68).

The authors suggest that the thione moiety, due to its susceptibility to oxidation, may exhibit unfavourable biochemical properties that limit its efficacy as a pharmacophore [6]. Modification of the thione group may improve the structure–activity relationship of the compound and increase its lipophilicity [37,38], therefore potentially enhancing physical properties. A series of 1,2,4-triazolo [3,4-*b*][1,3,4]thiadiazine derivatives **14a**–**d** was synthesised by the reaction of compound **12** with various 2-bromoacetophenones. NMR and FTIR data are presented in the Supplementary Materials (Figures S39–S46, and S58–S67). Notably, these reactions were carried out without the use of a base catalyst. The HBr released in the reaction acted as a catalyst for the cyclocondensation reaction, facilitating the formation of the fused heterocyclic system in compounds **14a**–**d**. Accordingly, the reaction mixtures were diluted with a 10% aqueous sodium acetate solution, and the synthesised compounds **14a**–**d** were isolated as free organic bases.

### 2.2. In Vitro Anticancer Activity of β-Phenylalanine Derivatives **2**–**14**

Non-proteogenic amino acid derivatives have previously been investigated for their promising pharmacological activity [39]. The chemical versatility of the amino acid scaffold enables the incorporation of various heterocyclic and aromatic substituents—many of which are present in approved or investigational pharmaceuticals [40]. Pyrazole-containing molecules have been extensively studied as antiproliferative and anticancer candidates, targeting various cancer cell types through mechanisms such as CDK and EGFR inhibition [41,42,43].

**Figure 1:**
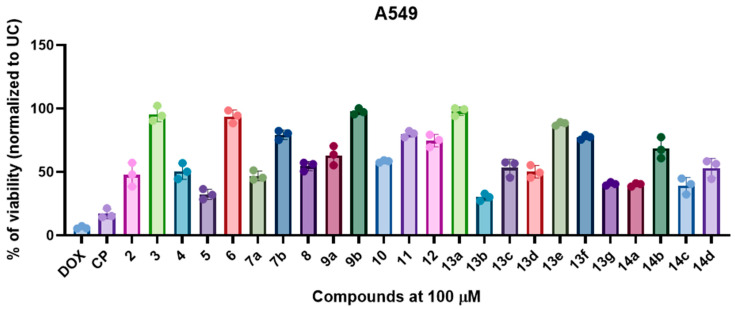
Non-proteogenic *β*-phenylalanine derivatives **2**–**14** demonstrated a structure-dependent antiproliferative activity in A549 cells. The cells were exposed to fixed 100 µM of compounds or doxorubicin (DOX), and cisplatin (CP) for 24 h, and the post-exposure viability was measured by MTT assay. The data is shown as mean ± SD of three experimental replicates.

After synthesising and characterising the non-proteogenic amino acid hybrids **2**–**14**, we evaluated their in vitro antiproliferative activity against A549 human lung adenocarcinoma cells (Figure 1). The antiproliferative effects of the synthesised compounds were assessed using the MTT assay, with results expressed as percentages of cell viability relative to untreated control cells. Doxorubicin (DOX) and cisplatin (CP), both FDA-approved chemotherapeutic agents commonly used in the treatment of lung neoplasms, served as positive controls. 

Among the tested compounds, *β*-phenylalanine derivatives exhibited substantial cytotoxic activity, significantly reducing (*p* < 0.05) A549 cell viability in a structure-dependent manner. Notably, compound **13b**, which features a 4-chlorophenyl substituent (R = 4-ClC_6_H_4_) on the Schiff base scaffold, demonstrated the most pronounced activity in the series, with a mean cell viability of 30.1%. This finding suggests a key role for electron-withdrawing halogen substituents in the para-position in enhancing antiproliferative efficacy. Similarly, compound **5**, a heterocyclic derivative containing a pyrazole moiety, also exhibited promising cytotoxicity, with cell viability reduced to 32.3% (Figure 1).

Compounds **14c** and **14a**, belonging to the series of 1,2,4-triazolo [3,4-*b*][1,3,4]thiadiazine derivatives, exhibited mean cell viability values of 39.3% and 40.1%, respectively. Compound **14c**, containing a 4-chlorophenyl substituent (R = 4-ClC_6_H_4_), further supports the observed trend that halogenation, particularly chlorine, enhances cytotoxic efficacy. Compound **13g**, featuring an *N*-ethyl-9*H*-carbazolyl moiety, yielded a similar viability (40.7%), suggesting that extended aromatic systems may also confer antiproliferative activity. Moderate antiproliferative activity was observed for semicarbazide **7a** (47.0%) and the precursor compound **2** (47.9%), indicating that early intermediates in the synthetic pathway retain biological activity. The hydrazide intermediate **4** (50.6%) and Schiff base **13d** (R = N(CH_3_)_2_C_6_H_4_, 50.2%) also displayed moderate activity, though less antiproliferative than their halogenated hybrids (Figure 1).

Compounds **8** (54.7%), **10** (58.4%), and **9a** (63.2%) demonstrated decreased antiproliferative activity in comparison to other compounds of this class. Interestingly, derivative **14b** (R = 4-FC_6_H_4_) displayed weaker antiproliferative activity (68.6%) compared to its chloro- and bromo-substituted derivatives, demonstrating that the electronegativity and steric effects of the halogen substituent significantly modulate biological activity (Figure 1). Interestingly, although the same pharmacophore scaffold was shared among the compounds, significant variation in antiproliferative activity was observed, depending on the nature of the attached side groups. High cell viability values (>90.0%) were exhibited by the pyrrole-containing compound **6**, methyl ester **3**, triazole-based compound **9b**, and Schiff base **13a** with an unsubstituted phenyl ring, indicating that minimal antiproliferative activity was induced under the tested conditions (Figure 1).

**Figure 2:**
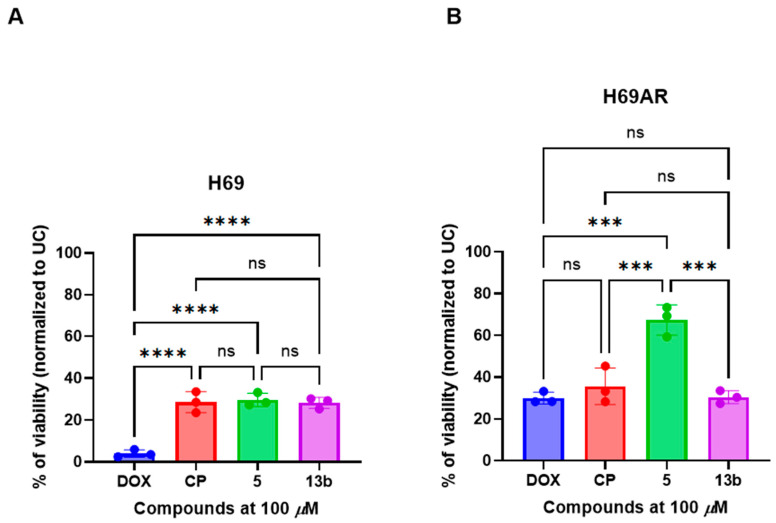
The non-proteogenic *β*-phenylalanine derivatives **5** and **13b** exhibited promising anticancer activity in both (**A**) drug-sensitive (H69) and (**B**) multidrug-resistant (H69AR) small cell lung cancer cell lines. Cells were treated with a fixed concentration (100 µM) of the compounds, doxorubicin (DOX), or cisplatin (CP) for 24 h, and cell viability was subsequently assessed using the MTT assay. Data are presented as the mean ± standard deviation (SD) of three independent experimental replicates. *** *p* < 0.002; **** *p* < 0.0001; ns—not significant.

After identifying the most promising compounds **5** and **13b**, we aimed to determine whether the observed antiproliferative activity in A549 cells was cell line-specific or extended to other lung-derived cancer cell models with defined multidrug resistance profiles. Compounds **5** and **13b** were, therefore, evaluated for their antiproliferative activity in H69 small cell lung cancer cells, including both anthracycline-sensitive and anthracycline-resistant variants (Figure 2A,B).

Compounds **5** and **13b** significantly reduced cell viability in drug-sensitive H69 cells compared to the untreated control (UC) (*p* < 0.05), indicating marked antiproliferative activity (Figure 2A). However, their efficacy was significantly lower than doxorubicin (*p* < 0.0001), yet statistically comparable to that of cisplatin (ns), suggesting a similar therapeutic potential to clinically employed platinum-based agents in this cell line.

In contrast, in multidrug-resistant H69AR cells (Figure 2B), compound **5** exhibited markedly reduced antiproliferative activity, with viability significantly higher than both doxorubicin and cisplatin (*p* < 0.002), indicating a loss of activity in the resistant phenotype. Notably, compound **13b** maintained its antiproliferative effect in H69AR cells, displaying activity comparable to doxorubicin (ns), and significantly higher than compound **5** (*p* < 0.002), suggesting its potential to overcome drug resistance.

In our study, the pyrazole derivative **5** demonstrated significant activity in drug-sensitive H69 cells, but this efficacy was diminished in the multidrug-resistant H69AR line. Triazole and thiadiazole analogues, such as compound **13b**, have also been reported to exhibit cytotoxic activity against lung cancer cell lines, including A549 [44].

**Figure 3:**
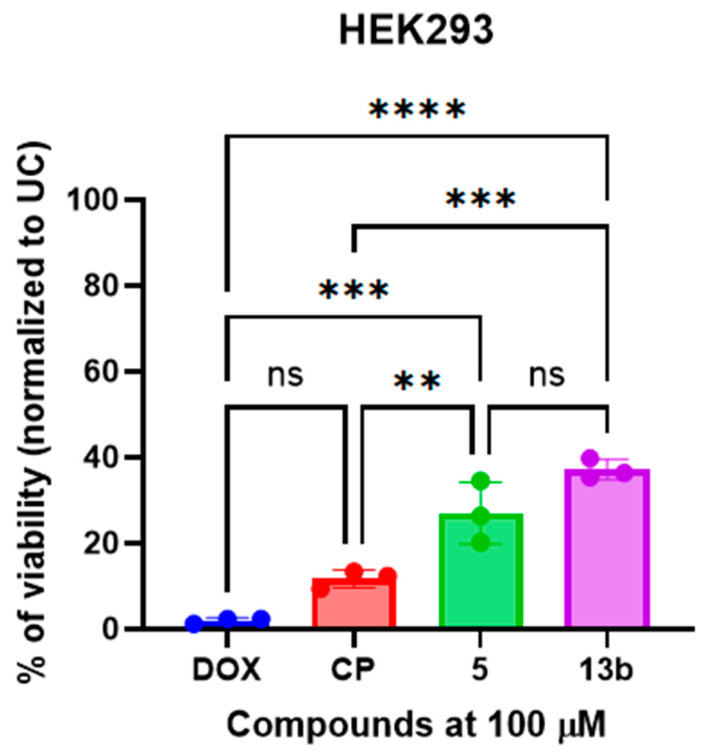
The non-proteogenic *β*-phenylalanine derivatives **5** and **13b** exhibited low cytotoxicity on non-cancerous HEK293 cells. Cells were treated with a fixed concentration (100 µM) of the compounds, doxorubicin (DOX), or cisplatin (CP) for 24 h, and cell viability was subsequently assessed using the MTT assay. Data are presented as the mean ± standard deviation (SD) of three independent experimental replicates. ** *p* < 0.01; *** *p* < 0.002; **** *p* < 0.0001; ns—not significant.

Following the identification of the most promising β-phenylalanine derivatives—compounds **5** and **13b**—based on their potent antiproliferative activity, we proceeded to evaluate whether this activity was selective for cancer cells. To assess selectivity, non-cancerous human embryonic kidney (HEK293) cells were treated with compounds **5** and **13b**, alongside cytotoxicity controls cisplatin (CP) and doxorubicin (DOX), and cell viability was subsequently assessed in vitro (Figure 3).

Compounds **5** and **13b** demonstrated low cytotoxicity in HEK293 cells, with viability levels comparable to or exceeding those observed following treatment with doxorubicin (DOX) and cisplatin (CP). Specifically, compounds **5** and **13b** exhibited significantly lower cytotoxicity (*p* < 0.05), as evidenced by increased cell viability following treatment, in comparison to doxorubicin (DOX) and cisplatin (CP) (Figure 3). No statistically significant difference in cytotoxicity was observed between compound **5** and compound **13b**.

Collectively, these results show the potential of non-proteogenic *β*-phenylalanine derivatives as a promising antiproliferative scaffold for targeting lung-derived neoplasms. Compound **13b** demonstrated consistent antiproliferative activity across both drug-sensitive and multidrug-resistant small cell lung cancer models, underscoring its potential to overcome chemoresistance and serve as a lead candidate for further development.

### 2.3. Compounds **5** and **13b** Induces Antiproliferative Activity in 3D A549 Spheroid Model

**Figure 4:**
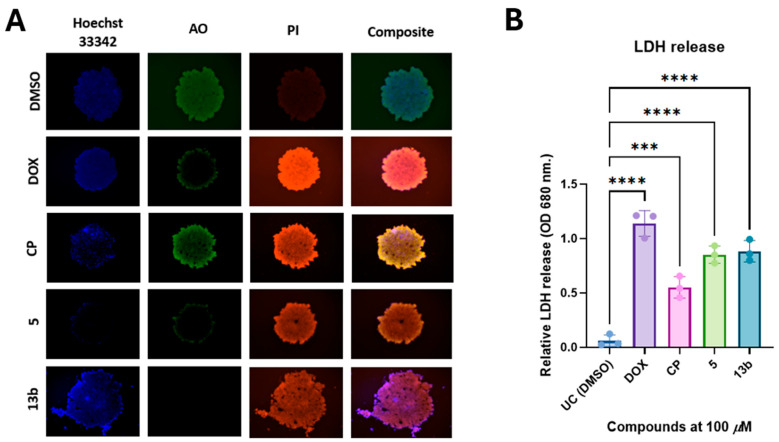
Non-proteogenic β-phenylalanine derivatives **5** and **13b** induce cell death in a 3D A549 spheroid model. (**A**) Representative images of AO/PI-stained A549 spheroids following 24 h treatment with test compounds, illustrating live (green) and dead (red) cells. (**B**) Quantification of compound-induced cytotoxicity measured by lactate dehydrogenase (LDH) release into the culture medium. Data are presented as mean ± standard deviation (SD) from three independent experiments. *** *p* < 0.002; **** *p* < 0.0001.

Upon identifying the most promising antiproliferative candidates and confirming their selective cytotoxicity towards malignant cells, subsequent investigations were undertaken to determine whether the antiproliferative effects observed in the 2D A549 cell culture model are preserved within a more complex, tumour-like 3D environment (Figure 4).

Treatment-induced cytotoxicity in A549 spheroids was assessed using acridine orange/propidium iodide (AO/PI) staining (Figure 4A). Exposure to doxorubicin (DOX) and cisplatin (CP) resulted in a pronounced decrease in acridine orange (AO) fluorescence, indicative of reduced cell viability, alongside an increase in propidium iodide (PI) staining, reflecting compromised membrane integrity and elevated levels of cell death. These results confirm that both DOX and CP induce significant cytotoxic effects in the 3D spheroid model. Consistent with the observed effects of DOX, treatment with compound **5**, and especially compound **13b**, resulted in marked PI uptake and a marked decrease in AO fluorescence, indicating significant cell death. Notably, spheroids treated with compound **13b** exhibited peripheral disintegration and significant morphological disruption, indicating a potent cytotoxic response that is comparable to or potentially exceeds that induced by DOX.

Quantitative validation of these observations was performed by measuring lactate dehydrogenase (LDH) release into the culture medium 24 h post-treatment (Figure 4B). Significantly elevated LDH levels were observed in spheroids treated with doxorubicin (DOX), compound **5**, and compound **13b**, in comparison to the untreated control (DMSO), thereby confirming treatment-induced disruption of membrane integrity. Among the test compounds **5** and **13b**, the highest level of LDH release was induced by Schiff base **13b**, comparable to that observed with DOX, while a significant increase was also elicited by pyrazole **5**. These results are consistent with the fluorescence microscopy findings and confirm that cytotoxic effects are exerted by both β-phenylalanine derivatives in 3D lung cancer spheroids, with compound **13b** exhibiting the greatest potency.

Collectively, these findings demonstrate that potent cytotoxic effects are exerted by the non-proteogenic β-phenylalanine derivatives against A549 lung cancer spheroids, with compound **13b** showing the highest efficacy. This suggests that Schiff base **13b** could be considered a hit compound for the development of further sub-libraries.

## 3. Materials and Methods

### 3.1. Chemistry

All reagents and solvents were obtained from Sigma-Aldrich (St. Louis, MO, USA) and used as received without further purification. The progress of reactions and the purity of synthesised compounds were monitored by thin-layer chromatography (TLC) on aluminium plates pre-coated with silica gel F_254_ (Merck KGaA, Darmstadt, Germany). ^1^H and ^13^C NMR spectra were recorded in DMSO-d_6_ at 25 °C using Bruker Avance III spectrometers (400 MHz for ^1^H, 101 MHz for ^13^C; and 700 MHz for ^1^H, 176 MHz for ^13^C; Bruker BioSpin AG, Fällanden, Switzerland). Chemical shifts (δ) are reported in ppm, referenced to DMSO-d_6_ (2.50 ppm for ^1^H and 39.43 ppm for ^13^C). The NMR spectrum of compound **2** was recorded in deuterated chloroform (CDCl_3_). Chemical shifts (δ) are reported in parts per million (ppm) and referenced to the residual solvent signal (7.26 ppm for ^1^H and 77.16 ppm for ^13^C). See Supplementary Materials, Figures S1–S46. Data are presented as chemical shift, multiplicity, coupling constant (Hz), integration, and signal assignment. Melting points were measured using a Büchi B-540 melting point apparatus (Büchi Corporation, New Castle, DE, USA) and are uncorrected. Mass spectrometric analyse for compound **13b** was performed using a Bruker maXis Ultra-High Resolution Time-of-Flight (UHR-TOF) mass spectrometer equipped with an electrospray ionisation (ESI) source (Bruker Corporation, Bremen, Germany). Elemental composition (C, H, N, S) of the synthesised compounds was determined using an EA3100 Series Elemental CHNSO Analyzer (EuroVector S.p.A., Pavia, Italy), operated with the dedicated Weaver software CFR 21 art.11. The instrument enables highly accurate quantification of carbon, hydrogen, nitrogen, and sulphur with results within ±0.3% of the theoretical values. Infrared (IR) spectra were recorded on a Perkin–Elmer Spectrum BX FT–IR spectrometer (Perkin–Elmer Inc., Waltham, MA, USA) using KBr pellets.

3-(4-Methylbenzene-1-sulfonamido)-3-phenylpropanoic acid (**2**)

(±)-*β*-Phenylalanine (**1**) (25.00 g, 0.15 mol) was dissolved in 200 mL of a 15% aqueous sodium carbonate solution and cooled to 0 °C in an ice bath. A solution of 4-methylbenzenesulfonyl chloride (35.00 g, 0.18 mol) in 50 mL of diethyl ether was prepared separately and added dropwise to the cooled, basic aqueous solution under vigorous stirring. Upon completion of the addition, the reaction mixture was allowed to warm to room temperature and stirred continuously for 24 h. Then the aqueous phase was separated and acidified to pH 2 with 20% hydrochloric acid. The resulting white crystalline precipitate was filtered, washed with water, and dried. Compound **2** was purified by dissolving it in 300 mL of a 10% aqueous sodium carbonate solution, then the solution was filtered, and acidified with 20% HCl to pH 2. This purification process was repeated twice. The final yield of compound **2** was 45.92 g (96%), m. p. 160–161 °C (ref. [20] 149–151 °C; [21] 159–157 °C). ^1^H NMR (400 MHz, CDCl_3_) *δ* (ppm): 2.39 (s, 3H, CH_3_), 2.99 (dd, 1H, *J* = 13.9, 6.5 Hz, CH_2_CH), 3.09 (dd, 1H, *J* = 13.9, 5.4 Hz, CH_2_CH), 4.05–4.31 (m, 1H, CHCH_2_), 5.10 (d, 1H, *J* = 8.9 Hz, SO_2_NH), 6.55 (br. s, 1H, OH), 6.99–7.12 (m, 2H, H_ar_), 7.15–7.30 (m, 5H, H_ar_), 7.59 (d, 2H, *J* = 7.9 Hz, H_ar_). ^13^C NMR (101 MHz, CDCl_3_): *δ* (ppm) 21.68 (CH_3_), 38.96 (CH_2_CH), 56.43 (CHNHSO_2_), 127.22, 127.51, 128.81, 129.58, 129.83, 134.76, 136.52, 143.96 (C_ar_), 175.46 (C=O). Anal. calcd for C_16_H_17_NO_4_S (319.38 g/mol) %: C, 60.17; H, 5.37; N, 4.39; S, 10.04. Found %: C, 60.02; H, 5.36; N, 4.67; S, 10.27.

Methyl 3-(4-methylbenzene-1-sulfonamido)-3-phenylpropanoate (**3**)

A mixture of compound **2** (45.00 g, 0.14 mol), methanol (200 mL), and concentrated sulfuric acid (5 mL) was refluxed for 8 h. Then the volatile components were removed under reduced pressure using a rotary evaporator. The residue was treated with 50 mL of a 10% aqueous sodium carbonate solution. The resulting crystals were collected by filtration, washed with water, and dried. The ester **3** was purified by recrystallisation from propan-2-ol. The yield of product **3** was 42.71 g (92%), m. p. 99–101 °C (ref. [22] 100–101 °C).

^1^H NMR (400 MHz, DMSO-d_6_) δ (ppm): 2.35 (s, 3H, CH_3_), 2.75 (dd, 1H, *J* = 13.7, 8.7 Hz, CH_2_CH), 2.90 (dd, 1H, *J* = 13.7, 6.5 Hz, CH_2_CH), 3.33 (s, 3H, OCH_3_), 3.93 (q, 1H, *J* = 8.0 Hz, CHNH), 7.10 (d, 2H, *J* = 7.4 Hz, H_ar_), 7.16–7.32 (m, 5H, H_ar_), 7.47 (d, 2H, *J* = 7.9 Hz, H_ar_), 8.42 (d, 1H, *J* = 8.4 Hz, SO_2_NH). ^13^C NMR (101 MHz, DMSO-d_6_) *δ* (ppm): 20.97 (CH_3_), 37.69 (CH_2_CH), 51.72 (CHNHSO_2_), 57.38 (OCH_3_), 126.35, 126.67, 128.27, 129.10, 129.35, 136.26, 137.82, 142.55 (C_ar_), 171.18 (C=O). Anal. calcd for C_17_H_19_NO_4_S (333.40 g/mol) %: C, 61.24; H, 5.74; N, 4.20; S, 9.62. Found %: C, 61.34; H, 5.72; N, 4.35; S, 9.45.

*N*-[3-Hydrazinyl-3-oxo-1-phenylpropyl]-4-methylbenzene-1-sulfonamide (**4**)

A mixture of the ester **3** (42.00 g, 0.13 mol), hydrazine monohydrate (20 mL, 19.50 g, 0.39 mol), and propan-2-ol (200 mL) was refluxed for 24 h. Then, the reaction solution was cooled down to room temperature. The precipitates were collected by filtration, washed with cold propan-2-ol, and dried. The hydrazide **4** was purified by recrystallisation from a mixture of propan-2-ol and water (1:5, *v*/*v*). The yield of product **4** was 31.64 g (73%), m. p. 145–147 °C (ref. [23] 148 °C). ^1^H NMR (400 MHz, DMSO-d_6_) *δ* (ppm): 2.34 (s, 3H, CH_3_), 2.59 (dd, 1H, *J* = 13.6, 8.7 Hz, CH_2_CH), 2.77 (dd, 1H, *J* = 13.6, 6.0 Hz, CH_2_CH), 3.86 (t, 1H, *J* = 7.4 Hz, CHNH), 4.05 (br. s, 2H, NH_2_), 6.99–7.25 (m, 7H, H_ar_), 7.44 (d, 2H, *J* = 7.9 Hz, H_ar_), 7.94 (s, 1H, NHNH_2_), 9.11 (s, 1H, SO_2_NH). ^13^C NMR (101 MHz, DMSO-d_6_) *δ* (ppm): 21.00 (CH_3_), 38.45 (CH_2_CH), 56.40 (CHNH), 126.30, 128.05, 129.16, 129.19, 137.05, 138.32, 142.16 (C_ar_), 169.51 (C=O). FTIR (KBr, ν, cm^−1^): 1154 (S=O), 1657 (C=O), 3171, 3282 (2NH), 3329, 3352 (NH_2_). Anal. calcd for C_16_H_19_N_3_O_3_S (333.41 g/mol) %: C, 57.64; H, 5.74; N, 12.60; S, 9.62. Found %: C, 57.51; H, 5.82; N, 12.57; S, 9.54.

*N*-[3-(3,5-Dimethyl-1*H*-pyrazol-1-yl)-3-oxo-1-phenylpropyl]-4-methylbenzenesulfonamide (**5**)

A mixture of hydrazide **4** (0.50 g, 1.50 mmol), 2,4-pentanedione (0.18 g, 1.80 mmol), and propan-2-ol (10 mL) was refluxed for 2 h in the presence of a few drops of acetic acid. Upon completion of the reaction (monitored by TLC, R*_f_* = 0.59 by eluent acetone:hexane 1:2), the mixture was allowed to cool to room temperature. The precipitate was filtered, washed with water, and dried. Product **5** was crystallised from a mixture of propan-2-ol and water (1:5, *v*/*v*) with a yield of 0.44 g (74%), m. p. 155–157 °C (ref. [23] 145–148 °C, [24] 120 °C).

^1^H NMR (400 MHz, DMSO-d_6_) *δ* (ppm): 2.19 (s, 3H, CH_3_), 2.25 (s, 3H, CH_3_), 2.29 (s, 3H, CH_3_), 2.74 (dd, 1H, *J* = 13.7, 9.9 Hz, CH_2_CH), 3.02 (dd, 1H, *J* = 13.7, 4.0 Hz, CH_2_CH), 5.20–5.40 (m, 1H, CHCH_2_), 6.17 (s, 0.92H, CH_Pyra_), 6.21 (s, 0.03H, CH_Pyra_), 6.26 (s, 0.05H, CH_Pyra_), 7.05–7.35 (m, 9H, H_ar_), 8.47 (d, 1H, *J* = 9.7 Hz, SO_2_NH). ^13^C NMR (101 MHz, DMSO-d_6_) *δ* (ppm): 13.60 (CH_3_), 13.66 (CH_3_), 20.84 (CH_3_), 37.56 (CH_2_CH), 56.21 (CHNHSO_2_), 111.70, 126.10, 126.51, 128.13, 129.07, 129.20, 136.80, 137.60, 142.33, 143.64, 152.35 (C_ar+Pyra_), 171.08 (C=O). FTIR (KBr, ν, cm^−1^): 1158 (S=O), 1675 (C=O), 3282 (NH). Anal. calcd for C_21_H_23_N_3_O_3_S (397.49 g/mol) %: C, 63.46; H, 5.83; N, 10.57; S, 8.07. Found %: C, 63.26; H, 5.85; N, 10.14; S, 7.94.

*N*-(2,5-Dimethyl-1*H*-pyrrol-1-yl)-3-[(4-methylphenyl)sulfonamido]-3-phenylpropanamide (**6**)

A mixture of hydrazide **4** (0.50 g, 1.50 mmol), 2,5-hexanedione (0.20 g, 1.80 mmol), and 1,4-dioxane (15 mL) was refluxed for 2 h in the presence of a catalytic amount of acetic acid. Upon completion of the reaction (monitored by TLC, R*_f_* = 0.54 by eluent acetone:hexane 1:2), volatile components were removed by rotary evaporation. The remaining residue was crystallised from a mixture of propan-2-ol and water (1:3, *v*/*v*). The compound **6** yield was 0.51 g (82%), m. p. 108–110 °C.

^1^H NMR (400 MHz, DMSO-d_6_) *δ* (ppm): 1.64 (s, 3H, CH_3_CN), 1.76 (s, 3H, CH_3_CN), 2.35 (s, 3H, CH_3_), 2.72 (dd, 1H, *J* = 13.7, 8.6 Hz, CH_2_CH), 2.96 (dd, 1H, *J* = 13.6, 6.5 Hz, CH_2_CH), 4.08 (q, 1H, *J* = 7.8 Hz, CHCH_2_), 5.56 (s, 2H, H_Pyr_), 6.63–7.32 (m, 7H, H_ar_), 7.52 (d, 2H, *J* = 7.9 Hz, H_ar_) 8.43 (d, 1H, *J* = 8.2 Hz, SO_2_NH), 10.83 (s, 1H, CONH**).** ^13^C NMR (101 MHz, DMSO-d_6_) *δ* (ppm): 10.56 (CH_3 Pyr_), 10.59 (CH_3 Pyr_), 20.96 (CH_3_), 38.53 (CH_2_CH), 55.97 (CHCH_2_), 102.90, 102.99, 126.27, 126.48, 126.60, 127.09, 128.22, 129.29, 129.40, 136.47, 138.40, 142.42 (C_ar+Pyr_), 169.96 (C=O). FTIR (KBr, ν, cm^−1^): 1154 (S=O), 1691 (C=N), 1713 (C=O), 3281, 3364 (2NH).Anal. calcd for C_22_H_25_N_3_O_3_S (411.52 g/mol) %: C, 64.21; H, 6.12; N, 10.21; S, 7.79. Found %: C, 64.04; H, 5.92; N, 10.05; S, 7.86.

General procedure for the synthesis of compounds **7a, b**

A mixture of compound **4** (2.50 g, 6.41 mmol), phenyl isothiocyanate (0.95 g, 7 mmol), or phenyl isocyanate (0.83 g, 7 mmol), and methanol (50 mL) was refluxed for 4 h. Then, the reaction mixture was cooled down to room temperature, and the resulting white crystals were filtered and dried. The obtained compounds **7a, b** were purified by recrystallisation from 1,4-dioxane.

2-[3-[(4-Methylphenyl)sulfonamido]-3-phenylpropanoyl]-*N*-phenylhydrazine-1-carboxamide (**7a**)

White solid, yield 2.08 g (72%), m. p. 177–179 °C. ^1^H NMR (400 MHz, DMSO-d_6_) *δ* (ppm): 2.32 (s, 3H, CH_3_), 2.68 (dd, 1H, *J* = 13.8, 9.7 Hz, CH_2_CH), 2.92 (dd, 1H, *J* = 13.8, 4.7 Hz, CH_2_CH), 3.89–4.07 (m, 1H, CHCH_2_), 6.90–7.52 (m, 14H, H_ar_), 8.12 (s, 1H, NH), 8.23 (d, 1H, *J* = 8.6 Hz, SO_2_NH), 8.51 (s, 1H, NH), 10.08 (s, 1H, NH). ^13^C NMR (101 MHz, DMSO-d_6_) *δ* (ppm): 20.96 (CH_3_), 37.88 (CH_2_CH), 56.17 (CHCH_2_), 118.20, 121.99, 126.24, 126.31, 128.03, 128.74, 129.21, 136.90, 137.96, 139.43, 142.19 (C_ar_), 154.91 (C=O), 170.53 (C=O). FTIR (KBr, ν, cm^−1^): 1153 (S=O), 1306 (C=S), 1690 (C=N), 3178, 3361 (2NH). Anal. calcd for C_23_H_24_N_4_O_4_S (452.53 g/mol) %: C, 61.05; H, 5.35; N, 12.38; S, 7.08. Found %: C, 61.01; H, 5.43; N, 12.67; S, 7.17.

2-[3-[(4-Methylphenyl)sulfonamido]-3-phenylpropanoyl]-*N*-phenylhydrazine-1-carbothioamide (**7b**)

White solid, yield 1.95 g (65%), m. p. 168–170 °C. ^1^H NMR (400 MHz, DMSO-d_6_) *δ* (ppm): 2.32 (s, 3H, CH_3_), 2.63–3.15 (m, 2H, CH_2_CH), 3.90 (br. s, 1H, CHCH_2_), 7.08–7.60 (m, 14H, H_ar_), 8.38 (br. s, 1H, 1 NH), 8.90 (br. s, 1H, 1 NH), 9.77 (br. s, 1H, 1 NH), 10.41 (s, 1H, 1 NH). ^13^C NMR (101 MHz, DMSO-d_6_) *δ* (ppm): 20.95 (CH_3_), 37.25 (CH_2_CH), 56.30 (CHCH_2_), 124.10, 124.87, 126.27, 126.36, 128.06, 128.28, 129.19, 129.28, 136.72, 137.49, 138.81, 142.40 (aromatic C), 170.59 (CH_2_CONH), 180.56 (C=S). FTIR (KBr, ν, cm^−1^): 1153 (S=O), 1306 (C=S), 1712 (C=O), 3027, 3060, 3178, 3262 (4NH). Anal. calcd for C_23_H_24_N_4_O_3_S_2_ (468.59 g/mol) %: C, 58.95; H, 5.16; N, 11.96; S, 13.68. Found %: C, 59.15; H, 5.11; N, 12.01; S, 13.62.

4-Methyl-*N*-[1-phenyl-2-[5-(phenylamino)-1,3,4-thiadiazol-2-yl]ethyl]benzenesulfonamide (**8**)

Compound **7b** (0.47 g, 1 mmol) was added to 5 mL of 80% sulphuric acid cooled in an ice bath (0–5 °C) with constant stirring for 5 min. The mixture was removed from the ice bath, allowed to warm to room temperature, and stirred for 24 h. The reaction progress was monitored by TLC (R*_f_* = 0.56 by eluent acetone:hexane 1:1.5). The reaction mixture was poured onto 50 g of crushed ice and stirred for 30 min, then the acidic solution was neutralised with 5% aqueous ammonia solution to pH 7. The precipitate was filtered, washed with cold water, and dried. The product was purified by recrystallisation from propan-2-ol to give compound **8** as white solid with a yield of 0.35 g (77%), m. p. 211–213 °C.

^1^H NMR (400 MHz, DMSO-d_6_) *δ* (ppm): 2.21 (s, 3H, CH_3_), 2.99 (dd, 1H, *J* = 13.9, 8.3 Hz, CH_2_CH), 3.10 (dd, 1H, *J* = 13.9, 7.0 Hz, CH_2_CH), 4.77 (q, 1H, *J* = 7.8 Hz, Hz, CHCH_2_), 6.99 (t, 1H, *J* = 7.4 Hz, H_ar_), 7.02–7.23 (m, 7H, H_ar_), 7.33 (t, 2H, *J* = 7.7 Hz, H_ar_), 7.41 (d, 2H *J* = 7.9 Hz, H_ar_), 7.51 (d, 2H, *J* = 8.0 Hz, H_ar_), 8.57 (d, 1H, *J* = 8.4 Hz, SO_2_NH), 10.20 (s, 1H, NH). ^13^C NMR (101 MHz, DMSO-d_6_) *δ* (ppm): 20.87 (CH_3_), 40.8 (CH_2_CH), 50.92 (CHCH_2_), 117.26, 121.86, 126.39, 126.51, 128.22, 129.09, 129.23, 129.26, 136.56, 137.87, 140.60, 142.34 (C_ar_), 161.08 (C=N), 164.93 (C=N-NH). FTIR (KBr, ν, cm^−1^): 1149 (S=O), 1552, 1605 (2C=N), 3135, 3263 (2NH). Anal. calcd for C_23_H_22_N_4_O_2_S_2_ (450.58 g/mol) %: C, 61.31; H, 4.92; N, 12.43; S, 14.23. Found %: C, 61.25; H, 4.74; N, 12.41; S, 14.36.

General procedure for the synthesis of compounds **9a, b**

A mixture of the corresponding semicarbazide **7a** or semithiocarbazide **7b** derivative and 20 mL of 4% aqueous sodium hydroxide solution was refluxed for 4 h. The reaction progress was monitored by TLC (R*_f_* = 0.31 (**9a**) and R*_f_* = 0.47 (**9b**) by eluent acetone:hexane 1:1.5). Then, the reaction mixture was cooled to room temperature and acidified with 20% hydrochloric acid to pH 6. The acidic mixture was heated to a boil and subsequently allowed to cool. The precipitate was filtered, washed with water, and dried. Products **9a** and **9b** were purified by recrystallisation from 1,4-dioxane.

4-Methyl-*N*-[2-(5-oxo-4-phenyl-4,5-dihydro-1*H*-1,2,4-triazol-3-yl)-1-phenylethyl]benzenesulfonamide (**9a**)

White solid, yield 0.29 g (67%), m. p. 204–206 °C**.** ^1^H NMR (700 MHz, DMSO-d_6_) *δ* (ppm): 2.34 (s, 3H, CH_3_), 2.79 (dd, 1H, *J* = 13.5, 7.4 Hz, CH_2_CH), 2.95 (dd, 1H, *J* = 13.5, 7.7 Hz, CH_2_CH), 3.99 (q, 1H, *J* = 7.1 Hz, CHNHSO_2_), 6.76–6.82 (m, 4H, H_ar_), 7.13–7.19 (m, 3H, H_ar_), 7.22 (d, 2H, *J* = 8.0 Hz, H_ar_), 7.37 (d, 2H, *J* = 8.2 Hz, H_ar_), 7.40–7.46 (m, 3H, H_ar_), 8.57 (d, 1H, *J* = 6.5 Hz, SO_2_NH), 11.84 (s, 1H, NH or OH). ^13^C NMR (176 MHz, DMSO-d_6_) *δ* (ppm): 20.99 (CH_3_), 40.02 (CH_2_CH), 50.59 (CHNHSO_2_), 126.24, 126.70, 127.48, 128.35, 128.82, 128.95, 129.30, 129.32, 131.92, 136.29, 137.53, 142.56 (C_ar_), 146.31 (C=N), 153.72 (NHC=O). FTIR (KBr, ν, cm^−1^): 1153 (S=O), 1586 (C=N), 1708 (C=O), 3091, 3167 (2NH). Anal. calcd for C_23_H_22_N_4_O_3_S (434.51 g/mol) %: C, 63.58; H, 5.10; N, 12.89; S, 7.38. Found %: C, 63.49; H, 5.07; N, 12.81; S, 7.23.

4-Methyl-*N*-[2-(5-thioxo-4-phenyl-4,5-dihydro-1*H*-1,2,4-triazol-3-yl)-1-phenylethyl]benzenesulfonamide (**9b**)

White solid, yield 0.31 g (69%), m. p. 224–226 °C. ^1^H NMR (400 MHz, DMSO-d_6_) *δ* (ppm): 2.33 (s, 3H, CH_3_), 2.82 (dd, 1H, *J* = 13.6, 7.9 Hz, CH_2_CH), 2.98 (dd, 1H, *J* = 13.6, 7.1 Hz, CH_2_CH), 3.90 (q, 1H, *J* = 7.5 Hz, CHNHSO_2_), 6.72 (d, 3H, *J* = 7.2 Hz, H_ar_), 6.92–7.22 (m, 6H, H_ar_), 7.31 (d, 2H, *J* = 7.9 Hz, H_ar_), 7.36–7.65 (m, 3H, H_ar_), 8.72 (d, 1H, *J* = 7.5 Hz, SO_2_NH), 13.90 (s, 1H, NH). ^13^C NMR (101 MHz, DMSO-d_6_) *δ* (ppm): 21.02 (CH_3_), 40.19 (CH_2_CH), 50.72 (CHNHSO_2_), 126.23, 126.83, 128.24, 128.42, 128.92, 129.37, 129.71, 132.63, 136.00, 137.05, 142.74 (C_ar_), 151.95 (C=N), 167.36 (C=S). FTIR (KBr, ν, cm^−1^): 1155 (S=O), 1306 (C=S), 1683 (C=N), 3247, 3281 (2NH). Anal. calcd for C_23_H_22_N_4_O_2_S_2_ (450.58 g/mol) %: C, 61.31; H, 4.92; N, 12.43; S, 14.23. Found %: C, 61.28; H, 4.89; N, 12.28; S, 14.15.

4-Methyl-*N*-[1-phenyl-2-(5-thioxo-4,5-dihydro-1,3,4-oxadiazol-2-yl)ethyl]benzenesulfonamide (**11**)

A mixture of potassium hydroxide (0.90 g, 16 mmol), carbon disulphide (0.61 g, 8 mmol), and 1-butanol (20 mL) was stirred at room temperature for 30 min. Afterwards, compound **4** (1.33 g, 4 mmol) was added, and the reaction mixture was refluxed for 48 h. Subsequently, the volatile components were removed under reduced pressure using a rotary evaporator. The resulting residue was dissolved in 10 mL of glacial acetic acid and brought to a boil. After cooling to room temperature, the solution was diluted with 20 mL of water. The precipitate was filtered, washed with water, and dried. Product **11** was purified by recrystallisation from methanol to afford white crystals with a yield of 1.38 g (92%), m. p. 182–184 °C. ^1^H NMR (400 MHz, DMSO-d_6_) *δ* (ppm): 2.34 (s, 3H, CH_3_), 3.03 (d, 2H, *J* = 7.2 Hz, CH_2_CH), 4.55 (q, 1H, *J* = 8.1 Hz, CHCH_2_), 7.09–7.28 (m, 7H, H_ar_), 7.46 (d, 2H, *J* = 8.0 Hz, H_ar_), 8.75 (d, 1H, *J* = 8.5 Hz, SO_2_NH), 14.25 (s, 1H, NH). ^13^C NMR (101 MHz, DMSO-d_6_) *δ* (ppm): 21.07 (CH_3_), 37.73 (CH_2_CH), 50.68 (CHCH_2_), 126.15, 126.89, 128.38, 129.11, 129.38, 135.54, 137.16, 142.90 (C_ar_), 161.24 (O-C=N), 177.53 (C=S). FTIR (KBr, ν, cm^−1^): 1159 (S=O), 1320 (C=S), 1682 (C=N), 3248, 3281 (2NH). Anal. calcd for C_17_H_17_N_3_O_3_S_2_ (375.46 g/mol) %: C, 54.38; H, 4.56; N, 11.19; S, 17.08. Found %: C, 54.23; H, 4.67; N, 11.22; S, 17.11.

*N*-[2-(4-Amino-5-thioxo-4,5-dihydro-1*H*-1,2,4-triazol-3-yl)-1-phenylethyl]-4-methylbenzenesulfonamide (**12**)

A mixture of potassium hydroxide (13.50 g, 0.24 mol), carbon disulphide (9.00 g, 0.12 mol), and 1-butanol (100 mL) was stirred at room temperature for 30 min. Afterwards, compound **4** (25.00 g, 0.06 mol) was added, and the reaction mixture was refluxed for 48 h. Then, hydrazine monohydrate (4 mL, 3.60 g, 0.07 mol) was added to the reaction mixture, and the reaction was continued under reflux for an additional 72 h. The volatile components were subsequently removed under reduced pressure via rotary evaporation. The residue was dissolved in 10 mL of glacial acetic acid and heated to boil, then the solution was allowed to cool slowly. Precipitate was formed, which was collected by filtration, washed with water, and dried. Product **12** was purified by recrystallisation from methanol to afford white crystals with a yield of 15.00 g (64%), m. p. 196–198 °C**.** ^1^H NMR (400 MHz, DMSO-d_6_) *δ* (ppm): 2.32 (s, 3H, CH_3_), 2.86 (dd, 1H, *J* = 13.7, 8.8 Hz, CH_2_CH), 3.11 (dd, 1H, *J* = 13.8, 6.3 Hz, CH_2_CH), 4.62 (q, 1H, *J* = 8.2 Hz, CHCH_2_), 5.38 (s, 2H, NH_2_), 7.05–7.26 (m, 7H, H_ar_), 7.41 (d, 2H, *J* = 8.1 Hz, H_ar_), 8.44 (d, 1H, *J* = 8.8 Hz, SO_2_NH), 13.40 (s, 1H, NH). ^13^C NMR (101 MHz, DMSO-d_6_) *δ* (ppm): 21.08 (CH_3_), 38.63 (CH_2_CH), 50.38 (CHCH_2_), 126.18, 126.59, 128.25, 129.08, 129.21, 136.73, 137.42, 142.57 (C_ar_), 151.04 (N-C=N), 166.37 (C=S). FTIR (KBr, ν, cm^−1^): 1154 (S=O), 1317 (C=S), 1598 (C=N), 3061, 3272, (2NH), 3362 (NH_2_). Anal. calcd for C_17_H_19_N_5_O_2_S_2_ (389.49 g/mol) %: C, 52.42; H, 4.92; N, 17.98; S, 16.46. Found %: C, 52.47; H, 4.77; N, 17.80; S, 16.18.

General procedure for the synthesis of compounds **13a**–**g**

A mixture of compound **12** (0.40 g, 1 mmol), the corresponding heterocyclic or aromatic aldehyde (1.2 mmol), and propan-2-ol (30 mL) was heated under reflux in the presence of a catalytic amount of hydrochloric acid for 8–24 h. After the reaction was finished, the solution was cooled to room temperature. The precipitate was filtered, washed with propan-2-ol, and dried. Products **13a**–**g** were purified by recrystallisation from propan-2-ol.

(*E*)-*N*-[2-[4-(Benzylideneamino)-5-thioxo-4,5-dihydro-1*H*-1,2,4-triazol-3-yl]-1-phenylethyl]-4-methylbenzenesulfonamide (**13a**)

White solid, yield 0.35 g (74%), m. p. 162–164 °C. ^1^H NMR (400 MHz, DMSO-d_6_) *δ* (ppm): 2.32 (s, 3H, CH_3_), 3.03–3.20 (m, 2H, CH_2_CH), 4.74 (q, 1H, *J* = 8.0 Hz, CHNHSO_2_), 7.00–7.23 (m, 7H, H_ar_), 7.41 (d, 2H, *J* = 8.0 Hz, H_ar_), 7.51–7.73 (m, 3H, H_ar_), 7.86 (d, 2H, *J* = 7.3 Hz, H_ar_), 8.67 (d, 1H, *J* = 8.2 Hz, SO_2_NH), 9.53 (s, 1H, N=CH), 13.76 (s, 1H, NHCS). ^13^C NMR (101 MHz, DMSO-d_6_) *δ* (ppm): 21.04 (CH_3_), 38.65 (CH_2_CH), 50.17 (CHNHSO_2_), 126.12, 126.75, 128.28, 128.71, 129.01, 129.10, 129.19, 131.97, 132.67, 136.23, 137.35, 142.74 (C_ar_), 149.43 (N=CH), 160.99 (C=N), 162.5 1 (C=S). FTIR (KBr, ν, cm^−1^): 1159 (S=O), 1336 (C=S), 1580, 1599 (C=N), 3032, 3250 (2NH). Anal. calcd for C_24_H_23_N_5_O_2_S_2_ (477.60 g/mol) %: C, 60.36; H, 4.85; N, 14.66; S, 13.43. Found %: C, 60.47; H, 4.54; N, 14.51; S, 13.34.

(*E*)-*N*-{2-[4-[(4-Chlorobenzylidene)amino]-5-thioxo-4,5-dihydro-1*H*-1,2,4-triazol-3-yl]-1-phenylethyl}-4-methylbenzenesulfonamide (**13b**)

White solid, yield 0.37 g (72%), m. p. 144–146 °C. ^1^H NMR (700 MHz, DMSO-d_6_) *δ* (ppm): 2.31 (s, 3H, CH_3_), 3.06–3.17 (m, 2H, CH_2_CH), 4.74 (q, 1H, *J* = 8.1 Hz, CHNHSO_2_), 7.05 (d, 2H, *J* = 6.5 Hz, H_ar_), 7.11–7.19 (m, 5H, H_ar_), 7.40 (d, 2H *J* = 8.2 Hz, H_ar_), 7.66 (d, 2H *J* = 8.5 Hz, H_ar_), 7.89 (d, 2H, *J* = 8.5 Hz, H_ar_), 8.67 (d, 1H, *J* = 8.2 Hz, SO_2_NH), 9.59 (s, 1H, N=CH), 13.78 (s, 1H, NH**).** ^13^C NMR (176 MHz, DMSO-d_6_) *δ* (ppm): 21.04 (CH_3_), 38.61 (CH_2_CH), 50.20 (CHNHSO_2_), 126.09, 126.76, 128.29, 129.03, 129.19, 129.27, 130.38, 130.90, 136.23, 137.32, 137.37, 142.76 (C_ar_), 149.35 (N=CH), 160.87 (C=N), 161.02 (C=S). FTIR (KBr, ν, cm^−1^): 1156 (S=O), 1330 (C=S), 1596, 1683 (2C=N), 3030, 3279 (2NH). Anal. calcd for C_24_H_22_ClN_5_O_2_S_2_ (512.04 g/mol) %: C, 56.30; H, 4.33; N, 13.68; S, 12.52. Found %: C, 56.31; H, 4.20; N, 13.52; S, 12.33. HRMS (ESI) Calcd. for C_24_H_22_ClN_5_O_2_S_2_ [M + H]^+^ 512.0976. Found 512.0978.

(*E*)-*N*-{2-[4-[(4-Bromobenzylidene)amino]-5-thioxo-4,5-dihydro-1*H*-1,2,4-triazol-3-yl]-1-phenylethyl}-4-methylbenzenesulfonamide (**13c**)

White solid, yield 0.45 g (82%), m. p. 128–130 °C. ^1^H NMR (400 MHz, DMSO-d_6_) *δ* (ppm): 2.32 (s, 3H, CH_3_), 3.02–3.21 (m, 2H, CH_2_CH), 4.73 (q, 1H, *J* = 8.0 Hz, CHNHSO_2_), 6.94–7.24 (m, 7H, H_ar_), 7.40 (d, 2H, *J* = 8.0 Hz, H_ar_), 7.80 (s, 4H, H_ar_), 8.66 (d, 1H, *J* = 8.2 Hz, SO_2_NH), 9.59 (s, 1H, N=CH), 13.78 (s, 1H, NH). ^13^C NMR (101 MHz, DMSO-d_6_) *δ* (ppm): 21.03 (CH_3_), 38.61 (CH_2_CH), 50.18 (CHNHSO_2_), 126.08, 126.34, 126.75, 128.28, 129.02, 129.18, 130.49, 131.22, 132.19, 136.23, 137.36, 142.74 (C_ar_), 149.34 (N=CH), 160.94 (C=N), 161.00 (C=S). FTIR (KBr, ν, cm^−1^): 1157 (S=O), 1325 (C=S), 1589 (2C=N), 3030, 3260 (2NH). Anal. calcd for C_24_H_22_BrN_5_O_2_S_2_ (556.50 g/mol) %: C, 51.80; H, 3.98; N, 12.58; S, 11.52. Found %: C, 52.00; H, 3.99; N, 12.39; S, 11.40.

(*E*)-*N*-{2-[4-[[4-(Dimethylamino)benzylidene]amino]-5-thioxo-4,5-dihydro-1*H*-1,2,4-triazol-3-yl]-1-phenylethyl}-4-methylbenzenesulfonamide (**13d**)

Yellow solid, yield 0.40 g (78%), m. p. 205–207 °C. ^1^H NMR (400 MHz, DMSO-d_6_) *δ* (ppm): 2.32 (s, 3H, CH_3_), 2.98–3.17 (m, 2H, CH_2_CH), 3.05 (s, 6H, N(CH_3_)_2_), 4.68 (q, 1H, *J* = 8.0 Hz, CHNHSO_2_), 6.83 (d, 2H, *J* = 8.7 Hz, H_ar_), 6.97–7.05 (m, 2H, H_ar_), 7.11–7.22 (m, 5H, H_ar_), 7.41 (d, 2H, *J* = 8.0 Hz, H_ar_), 7.64 (d, 2H, *J* = 8.6 Hz, H_ar_), 8.60 (d, 1H, *J* = 8.2 Hz, SO_2_NH), 8.96 (s, 1H, N=CH), 13.62 (s, 1H, NH). ^13^C NMR (101 MHz, DMSO-d_6_) *δ* (ppm): 21.04 (CH_3_), 38.82 (CH_2_CH), 39.68 (N(CH_3_)_2_), 50.17 (CHNHSO_2_), 111.52, 118.55, 126.16, 126.68, 128.26, 128.97, 129.19, 130.45, 136.27, 137.41, 142.63, 149.36 (C_ar_), 153.17 (N=CH), 160.93 (C=N), 164.06 (C=S). FTIR (KBr, ν, cm^−1^): 1157 (S=O), 1323 (C=S), 1586, 1613 (2C=N), 3048, 3278 (2NH). Anal. calcd for C_26_H_28_N_6_O_2_S_2_ (520.67 g/mol) %: C, 59.98; H, 5.42; N, 16.14; S, 12.31. Found %: C, 60.12; H, 5.57; N, 16.24; S, 12.37.

(*E*)-4-Methyl-*N*-{1-phenyl-2-[4-[(thiophen-2-ylmethylene)amino]-5-thioxo-4,5-dihydro-1*H*-1,2,4-triazol-3-yl]ethyl}benzenesulfonamide (**13e**)

Light yellowish solid, yield 0.33 g (69%), m. p. 176–178 °C. ^1^H NMR (400 MHz, DMSO-d_6_) δ (ppm): 2.33 (s, 3H, CH_3_), 2.95–3.15 (m, 2H, CH_2_CH), 4.69 (q, 1H, *J* = 8.0 Hz, CHNHSO_2_), 6.99–7.21 (m, 7H, H_ar_), 7.33–7.24 (m, 1H, H_Thioph_), 7.40 (d, 2H, *J* = 8.1 Hz, H_ar_), 7.77 (d, 1H, *J* = 3.5 Hz, H_Thioph_), 7.99 (d, 1H, *J* = 5.0 Hz, H_Thioph_), 8.65 (d, 1H, *J* = 8.3 Hz, SO_2_NH), 9.73 (s, 1H, N=CH), 13.77 (s, 1H, NH). ^13^C NMR (101 MHz, DMSO-d_6_) *δ* (ppm): 21.05 (CH_3_), 40.19 (CH_2_CH), 49.96 (CHNHSO_2_), 126.16, 126.74, 128.24, 128.53, 128.97, 129.20, 133.09, 135.72, 136.15, 136.40, 137.26, 142.71 (C_ar+Thioph_), 149.80 (N=CH), 156.51 (C=N), 160.94 (C=S). FTIR (KBr, ν, cm^−1^): 1155 (S=O), 1326 (C=S), 1574, 1592 (2C=N), 3061, 3291 (2NH). Anal. calcd for C_22_H_21_N_5_O_2_S_3_ (483.62 g/mol) %: C, 54.64; H, 4.38; N, 14.48; S, 19.89. Found %: C, 54.40; H, 4.17; N, 14.26; S, 19.75.

(*E*)-*N*-[2-{4-[[(1*H*-Indol-3-yl)methylene]amino]-5-thioxo-4,5-dihydro-1*H*-1,2,4-triazol-3-yl}-1-phenylethyl]-4-methylbenzenesulfonamide (**13f**)

Greyish solid, yield 0.33 g (64%), m. p. 136–138 °C. ^1^H NMR (400 MHz, DMSO-d_6_) *δ* (ppm): 2.29 (s, 3H, CH_3_), 3.02 (dd, 1H, *J* = 13.5, 7.5 Hz, CH_2_CH), 3.14 (dd, 1H, *J* = 13.4, 8.2 Hz, CH_2_CH), 4.76 (q, 1H, *J* = 7.9 Hz, CHNHSO_2_), 7.01 (d, 2H, *J* = 6.8 Hz, H_ar_), 7.07–7.21 (m, 6H, H_ar_), 7.24 (t, 1H, *J* = 7.5 Hz, H_ar_), 7.31 (t, 1H, *J* = 7.6 Hz, H_ar_), 7.41 (d, 2H, *J* = 7.9 Hz, H_ar_), 7.55 (d, 1H, *J* = 8.0 Hz, H_ar_), 8.10 (d, 2H, *J* = 7.6 Hz, H_ar_), 8.67 (s, 1H, SO_2_NH), 9.12 (s, 1H, N=CH), 12.07 (s, 1H, NH_Indol_), 13.63 (s, 1H, NH). ^13^C NMR (101 MHz, DMSO-d_6_) *δ* (ppm): 21.03 (CH_3_), 40.19 (CH_2_CH), 49.94 (CHNHSO_2_), 103.29, 109.91, 112.39, 121.50, 122.13, 123.33, 124.21, 126.25, 126.75, 128.25, 128.97, 129.23, 135.53, 136.20, 137.18, 137.43, 142.65, 149.82 (C_ar+Indol_), 160.76 (N=CH), 161.42 (C=N), 166.37 (C=S). FTIR (KBr, ν, cm^−1^): 1156 (S=O), 1320 (C=S), 1574, 1593 (2C=N), 3030, 3092, 3337 (3NH). Anal. calcd for C_26_H_24_N_6_O_2_S_2_ (516.64 g/mol) %: C, 60.45; H, 4.68; N, 16.27; S, 12.41. Found %: C, 60.58; H, 4.75; N, 16.15; S, 12.19.

(*E*)-*N*-[2-{4-[[(9-Ethyl-9*H*-carbazol-3-yl)methylene]amino]-5-thioxo-4,5-dihydro-1*H*-1,2,4-triazol-3-yl}-1-phenylethyl]-4-methylbenzenesulfonamide (**13g**)

Brown solid, yield 0.49 g (83%), m. p. 189–191 °C. ^1^H NMR (400 MHz, DMSO-d_6_) δ (ppm): 1.37 (t, *J* = 7.0 Hz, 3H, CH_3_CH_2_), 2.31 (s, 3H, CH_3_), 3.01–3.28 (m, 2H, CH_2_CH), 4.54 (q, *J* = 7.1 Hz, 2H, CH_3_CH_2_), 4.79 (q, 1H, *J* = 8.0 Hz, CHNHSO_2_), 6.96–7.25 (m, 7H, H_ar_), 7.32 (t, 1H, *J* = 7.5 Hz, H_Carb_), 7.45 (d, 2H, *J* = 8.0 Hz, H_ar_), 7.55 (t, 1H, *J* = 7.7 Hz, H_Carb_), 7.71 (d, 1H, *J* = 8.2 Hz, H_Carb_), 7.82 (d, 1H, *J* = 8.7 Hz, H_Carb_), 8.05 (d, 1H, *J* = 8.6 Hz, H_Carb_), 8.31 (d, 1H, *J* = 7.8 Hz, H_Carb_), 8.61 (s, 1H, *J* = 1.5 Hz, H_Carb_), 8.68 (d, 1H *J* = 8.1 Hz, SO_2_NH), 9.52 (s, 1H, N=CH), 13.72 (s, 1H, NH). ^13^C NMR (101 MHz, DMSO-d_6_) *δ* (ppm): 13.78 (CH_3_CH_2_), 21.03 (CH_3_), 37.34 (CH_3_CH_2_), 38.73 (CH_2_CH), 50.25 (CHNHSO_2_), 109.78, 109.93, 119.89, 120.82, 122.16, 122.36, 122.67, 123.15, 125.39, 126.18, 126.58, 126.74, 128.29, 129.05, 129.20, 136.34, 137.40, 140.12, 142.07, 142.69 (C_ar+Carb_), 149.45 (N=CH), 161.03 (C=N), 164.15 (C=S). FTIR (KBr, ν, cm^−1^): 1158 (S=O), 1327 (C=S), 1586, 1630 (2C=N), 3031, 3264 (2NH). Anal. calcd for C_32_H_30_N_6_O_2_S_2_ (594.75 g/mol) %: C, 64.62; H, 5.08; N, 14.13; S, 10.78. Found %: C, 64.54; H, 5.05; N, 14.27; S, 10.54.

General procedure for the synthesis of compounds **14a–d**

A mixture of compound **12** (0.40 g, 1.00 mmol), the corresponding aromatic α-haloketone (1.20 mmol), and 1,4-dioxane (30 mL) was heated under reflux for 8–18 h. The progress of the reaction was monitored by TLC by eluent acetone:hexane 1:2. Afterwards, the reaction mixture was allowed to cool to room temperature, and the solution was diluted with 30 mL of a 10% aqueous sodium acetate solution. The precipitate was filtered, washed with propan-2-ol, and dried. Products **14**(**a**–**d**) were purified by recrystallisation from 1,4-dioxane.

4-Methyl-*N*-[1-phenyl-2-(6-phenyl-7*H*-[1,2,4]triazolo[3,4-*b*][1,3,4]thiadiazin-3-yl)ethyl]benzenesulfonamide (**14a**)

Light brownish solid, yield 0.31 g (64%), m. p. 207–209 °C. ^1^H NMR (400 MHz, DMSO-d_6_) *δ* (ppm): 2.36 (s, 3H, CH_3_), 3.17 (p, 2H, *J* = 13.5 Hz, CH_2_CH), 3.92–4.26 (m, 2H, CH_2_S), 4.88 (q, 1H, *J* = 8.0 Hz, CHNHSO_2_), 6.99–7.24 (m, 7H, H_ar_), 7.42 (d, 2H, *J* = 7.9 Hz, H_ar_), 7.52–7.72 (m, 3H, H_ar_), 7.97 (d, 2H, *J* = 7.5 Hz, H_ar_), 8.72 (d, 1H, *J* = 8.1 Hz, SO_2_NH). ^13^C NMR (101 MHz, DMSO-d_6_) *δ* (ppm): 20.93 (CH_3_), 22.59 (CH_2_S), 40.19 (CH_2_CH), 49.72 (CHNHSO_2_), 126.20, 126.62, 127.62, 128.28, 129.01, 129.11, 132.03, 133.18, 136.59, 137.76, 140.00 (C_ar_), 142.39 (C=N), 151.92 (C=N), 154.31 (C=N). FTIR (KBr, ν, cm^−1^): 1159 (S=O), 1569, 1597, 1640 (3C=N), 3085 (NH). Anal. calcd for C_25_H_23_N_5_O_2_S_2_ (489.61 g/mol) %: C, 61.33; H, 4.74; N, 14.30; S, 13.10. Found %: C, 61.18; H, 4.91; N, 14.27; S, 13.08.

*N*-{2-[6-(4-Fluorophenyl)-7*H*-[1,2,4]triazolo[3,4-*b*][1,3,4]thiadiazin-3-yl]-1-phenylethyl}-4-methylbenzenesulfonamide (**14b**)

Light yellowish solid, yield 0.40 g (78%), m. p. 174–176 °C. ^1^H NMR (700 MHz, DMSO-d_6_) *δ* (ppm): 2.26 (s, 3H, CH_3_), 3.12–3.24 (m, 2H, CH_2_CH), 3.99–4.13 (m, 2H, CH_2_S), 4.83–4.91 (m, 1H, CHNHSO_2_), 7.05 (d, 2H *J* = 7.1 Hz, H_ar_), 7.08–7.14 (m, 3H, H_ar_), 7.17 (t, 2H, *J* = 7.4 Hz, H_ar_), 7.40 (d, 2H, *J* = 8.2 Hz, H_ar_), 7.46 (t, 2H, *J* = 8.8 Hz, H_ar_), 8.00–8.09 (m, 2H, H_ar_), 8.71 (d, 1H, *J* = 8.5 Hz, SO_2_NH). ^13^C NMR (176 MHz, DMSO-d_6_) *δ* (ppm): 20.89 (CH_3_), 22.51 (CH_2_S), 40.02 (CH_2_CH), 49.73 (CHNHSO_2_), 116.03, 116.15, 126.14, 126.59, 128.25, 129.09, 129.64, 129.65, 130.26, 130.31, 136.57, 137.77, 139.89, 142.34, 151.79 (C_ar_), 153.28 (C=N), 163.62 (C=N), 165.05 (C=N). FTIR (KBr, ν, cm^−1^): 1160 (S=O), 1599, 1654 (3C=N), 3255 (NH). Anal. calcd for C_25_H_22_FN_5_O_2_S_2_ (507.60 g/mol) %: C, 59.16; H, 4.37; N, 13.80; S, 12.63. Found %: C, 59.17; H, 4.32; N, 13.89; S, 12.68.

*N*-{2-[6-(4-Chlorophenyl)-7*H*-[1,2,4]triazolo[3,4-*b*][1,3,4]thiadiazin-3-yl]-1-phenylethyl}-4-methylbenzenesulfonamide (**14c**)

White solid, yield 0.39 g (75%), m. p. 162–164 °C. ^1^H NMR (700 MHz, DMSO-d_6_) *δ* (ppm): 2.26 (s, 3H, CH_3_), 3.10–3.24 (m, 2H, CH_2_CH), 3.98–4.15 (m, 2H, CH_2_S), 4.87 (q, 1H, *J* = 8.2 Hz, CHNHSO_2_), 7.05 (d, 2H, *J* = 6.8 Hz, H_ar_), 7.09–7.14 (m, 3H, H_ar_), 7.17 (t, 2H, *J* = 7.5 Hz, H_ar_), 7.40 (d, 2H, *J* = 8.2 Hz, H_ar_), 7.68 (d, 2H, *J* = 8.7 Hz, H_ar_), 7.99 (d, 2H, *J* = 8.7 Hz, H_ar_), 8.71 (d, 1H, *J* = 8.5 Hz, SO_2_NH). ^13^C NMR (176 MHz, DMSO-d_6_) *δ* (ppm): 20.89 (CH_3_), 22.42 (CH_2_S), 40.02 (CH_2_CH), 49.73 (CHNHSO_2_), 126.14, 126.59, 128.26, 129.07, 129.09, 129.09, 129.40, 131.96, 136.55, 136.88, 137.74, 139.91 (C_ar_), 142.34 (C=N), 151.82 (C=N), 153.24 (C=N). FTIR (KBr, ν, cm^−1^): 1159 (S=O), 1560, 1591, 1663 (3C=N), 3257 (NH). Anal. calcd for C_25_H_22_ClN_5_O_2_S_2_ (524.05 g/mol) %: C, 57.30; H, 4.23; N, 13.36; S, 12.24. Found %: C, 57.19; H, 4.05; N, 13.48; S, 12.09.

*N*-{2-[6-(4-Bromophenyl)-7*H*-[1,2,4]triazolo[3,4-*b*][1,3,4]thiadiazin-3-yl]-1-phenylethyl}-4-methylbenzenesulfonamide (**14d**)

Light yellowish solid, yield 0.41 g (72%), m. p. 140–142 °C. ^1^H NMR (700 MHz, DMSO-d_6_) *δ* (ppm): 2.26 (s, 3H, CH_3_), 3.12–3.21 (m, 2H, CH_2_CH), 3.99–4.13 (m, 2H, CH_2_S), 4.78–4.92 (m, 1H, CHNHSO_2_), 7.05 (d, 2H, *J* = 6.8 Hz, H_ar_), 7.08–7.14 (m, 3H, H_ar_), 7.17 (t, 2H, *J* = 7.4 Hz, H_ar_), 7.40 (d, 2H, *J* = 8.2 Hz, H_ar_), 7.82 (d, 2H, *J* = 8.6 Hz, H_ar_), 7.91 (d, 2H, *J* = 8.6 Hz, H_ar_), 8.71 (d, 1H, *J* = 8.5 Hz, SO_2_NH). ^13^C NMR (176 MHz, DMSO-d_6_) *δ* (ppm): 20.89 (CH_3_), 22.39 (CH_2_S), 40.02 (CH_2_CH), 49.74 (CHNHSO_2_), 125.89, 126.11, 126.15, 126.59, 128.26, 128.31, 129.08, 129.10, 129.13, 129.54, 130.43, 132.00, 132.33, 136.55, 137.74, 139.92 (C_ar_), 142.35 (C=N), 151.83 (C=N), 153.38 (C=N). FTIR (KBr, ν, cm^−1^): 1159 (S=O), 1558, 1586, 1681 (3C=N), 3259 (NH). Anal. calcd for C_25_H_22_BrN_5_O_2_S_2_ (568.51 g/mol) %: C, 52.82; H, 3.90; N, 12.32; S, 11.28. Found %: C, 52.87; H, 3.93; N, 12.30; S, 11.05.

### 3.2. Cell Lines and Culture Conditions

The A549 non-small cell human lung carcinoma cells (ATCC CCL-185), H69 (HTB-19), H69AR (CRL-11351), and HEK293 were obtained from the American Type Culture Collection (Rockville, MD, USA). H69 and H69AR RPMI-1640 media 10% foetal bovine serum (FBS) (Gibco, Waltham, MA, USA), 100 U/mL penicillin, and 100 μg/mL streptomycin (P/S) (Gibco, Waltham, MA, USA). A549 and Vero were cultivated in Dulbecco’s Modified Eagle Medium/Nutrient Mixture F-12 (DMEM/F-12) (Gibco, Waltham, MA, USA), 10% foetal bovine serum (FBS) (Gibco, Waltham, MA, USA), 100 U/mL penicillin, and 100 μg/mL streptomycin (P/S) (Gibco, Waltham, MA, USA). Culturing conditions were maintained at 37 °C with a humidified atmosphere containing 5% CO_2_. The culture medium was refreshed every 2–3 days, and cells were passaged when they reached 70–80% confluence.

### 3.3. Cell Viability Assay

The in vitro inhibitory effects of the compounds were measured by MTT assay. Cells were plated in 96-well plates at a density of 1 × 10^4^ cells/well [45,46]. For HEK293 experiments, tissue culture plate were coated with poly-D-lysine (Gibco, Thermo Fisher Scientific) prior seeding the cells. After overnight attachment at 37 °C, 5% CO_2_, cells were treated with compounds (100 µM) in triplicate. After 20 h of treatment, the MTT reagent was added, and cells were further incubated for 4 h. The formazan was then extracted with anhydrous DMSO. The samples were measured using a microplate reader at a wavelength of 570 nm. The following formula was used to calculate the percentage of A549 viability: ([AE−AB]/[AC−AB]) × 100%. AE, AC, and AB were defined as the absorbance of experimental samples, untreated samples, and blank controls, respectively. The data were analysed using GraphPad Prism version 10.0 or QuickCalcs.

### 3.4. Generation of A549 3D Spheroids

Briefly, 96-well plates were pre-coated with 50 µL of 1% agarose prepared in Dulbecco’s Phosphate-Buffered Saline (DPBS). A549 cells were then seeded at a density of 2.5 × 10^5^ cells per well and incubated for 48 h to allow spheroid formation. Following spheroid establishment, cells were treated with 100 µM of the test compounds, prepared in DMEM/F12 medium supplemented with 10% foetal bovine serum (FBS) and 0.25% DMSO, and incubated for an additional 24 h.

### 3.5. Acridine Orange/Propidium Iodide (AO/PI) Staining of A549 Spheroids

Following compound treatment, A549 spheroids were incubated with 5 µg/mL acridine orange, 5 µg/mL propidium iodide, and 2.5 µg/mL of Hoechst for 30 min at 37 °C in a humidified incubator. After staining, spheroids were washed twice with Dulbecco’s Phosphate-Buffered Saline (DPBS) to remove excess dye. Fluorescent imaging was performed using the EVOS Cell Imaging System (Thermo Fisher Scientific, Waltham, MA, USA). Spheroid viability was evaluated based on differential fluorescence: viable cells emitted green fluorescence due to acridine orange uptake, whereas non-viable cells exhibited red fluorescence from propidium iodide, indicating loss of membrane integrity.

### 3.6. Statistical Analysis

The data are expressed as mean ± SD values from three separate experiments unless stated otherwise. The statistical significance was determined using a one-way ANOVA test. Data were considered significant when *p* < 0.05.

## 4. Conclusions

In this study, a series of novel non-proteogenic *β*-phenylalanine derivatives were synthesised and structurally modified to incorporate various heterocyclic moieties, including pyrazole, pyrrole, triazole, thiadiazole, oxadiazole, and Schiff bases. All synthesised compounds **2**–**14** were subjected to biological evaluation for their anticancer activity against lung cancer cell lines. Initial screening against the A549 human lung adenocarcinoma cell line led to the identification of pyrazole derivative **5** and Schiff base **13b** as the most active compounds. These were subsequently evaluated in H69 and H69AR small cell lung cancer models to determine their efficacy in both drug-sensitive and multidrug-resistant phenotypes.

Potent antiproliferative activity was retained by compound **13b**, which contains a 4-chlorophenyl substituent, in both cell lines, with performance comparable to that of cisplatin. This suggests that resistance mechanisms commonly associated with standard chemotherapeutics may be overcome. In contrast, compound **5** exhibited reduced efficacy in the resistant H69AR line. Furthermore, compounds **5** and **13b** demonstrated favourably low cytotoxicity towards non-cancerous HEK293 cells.

Overall, the *β*-phenylalanine scaffold was demonstrated to be a versatile platform for the development of azole-containing sulphonamide derivatives with significant anticancer potential. The specific activity of compound **13b** in drug-resistant cancer models underscores its potential as a promising lead for further optimisation and advancement in preclinical studies, particularly targeting lung cancer and chemotherapy-resistant tumours. Further studies are needed to better understand the in vivo activity, cellular targets and molecular activity mechanisms induced by antiproliferative *β*-phenylalanine derivatives and hit compounds **5** and **13b.**

## Data Availability

The original contributions presented in this study are included in the article; further inquiries can be directed to the corresponding authors.

## Supplementary Materials

The following supporting information can be downloaded at: https://www.mdpi.com/article/10.3390/molecules30153303/s1, Figures S1–S46: NMR spectra; Figures S47–S67: FTIR spectra; Figure S68: HRMS spectrum of compound **13b**; References [47,48].
