# Supplementary material for: Synthesis and Biological Evaluation of β-Phenylalanine Derivatives Containing Sulphonamide and Azole Moieties as Antiproliferative Candidates in Lung Cancer Models
Source: Molecules. 2025 Aug 7;30(15):3303. doi: 10.3390/molecules30153303 (PMC12348707; doi:10.3390/molecules30153303)
Supplement: Supplementary file 1 [file molecules-30-03303-s001.zip › molecules-3777669-supplementary.pdf]

Synthesis and Biological Evaluation of β-Phenylalanine Derivatives Containing Sulphonamide and Azole Moieties as Antiproliferative Candidates in Lung Cancer Models

Vytautas Mickevičius [1], Kazimieras Anusevičius [1,*], Birutė Sapijanskaitė-Banevič [1], Ilona Jonuškienė [1], Linas Kapočius [1], Birutė Grybaitė [1], Ramunė Grigalevičiūtė [2,3] , and Povilas Kavaliauskas [1,2,4,5]

[1] Department of Organic Chemistry, Kaunas University of Technology, Radvilenu˛ Rd. 19, LT-50254 Kaunas, Lithuania; vytautas.mickevicius@ktu.lt (V.M.); birute.sapijanskaite@ktu.lt (B.S.-B.); ilona.jonuskiene@ktu.lt (I.J.); l.kapocius@ktu.edu (L.K.); birute.grybaite@ktu.lt (B.G.); povilas.kavaliauskas@som.umaryland.edu (P.K.)

[2] Biological Research Centre, Lithuanian University of Health Sciences, Tilžės g. 18, LT-47181 Kaunas, Lithuania; ramune.grigaleviciute@lsmu.lt

[3] Department of Animal Nutrition, Lithuanian University of Health Sciences, Tilžės g. 18, LT-47181 Kaunas, Lithuania

[4] Department of Microbiology and Immunology, University of Maryland School of Medicine, Baltimore, MD 21201, USA

[5] Institute of Infectious Diseases and Pathogenic Microbiology, Birštono Str. 38A, LT-59116 Prienai, Lithuania

* Correspondence: kazimieras.anusevicius@ktu.lt; Tel.: +37-064-621-841

Supplementary materials

In the spectral data of some of the synthesized compounds **2–14**, the observed impurities were marked and identified, based on literature references [1,2], as residual solvent or water.

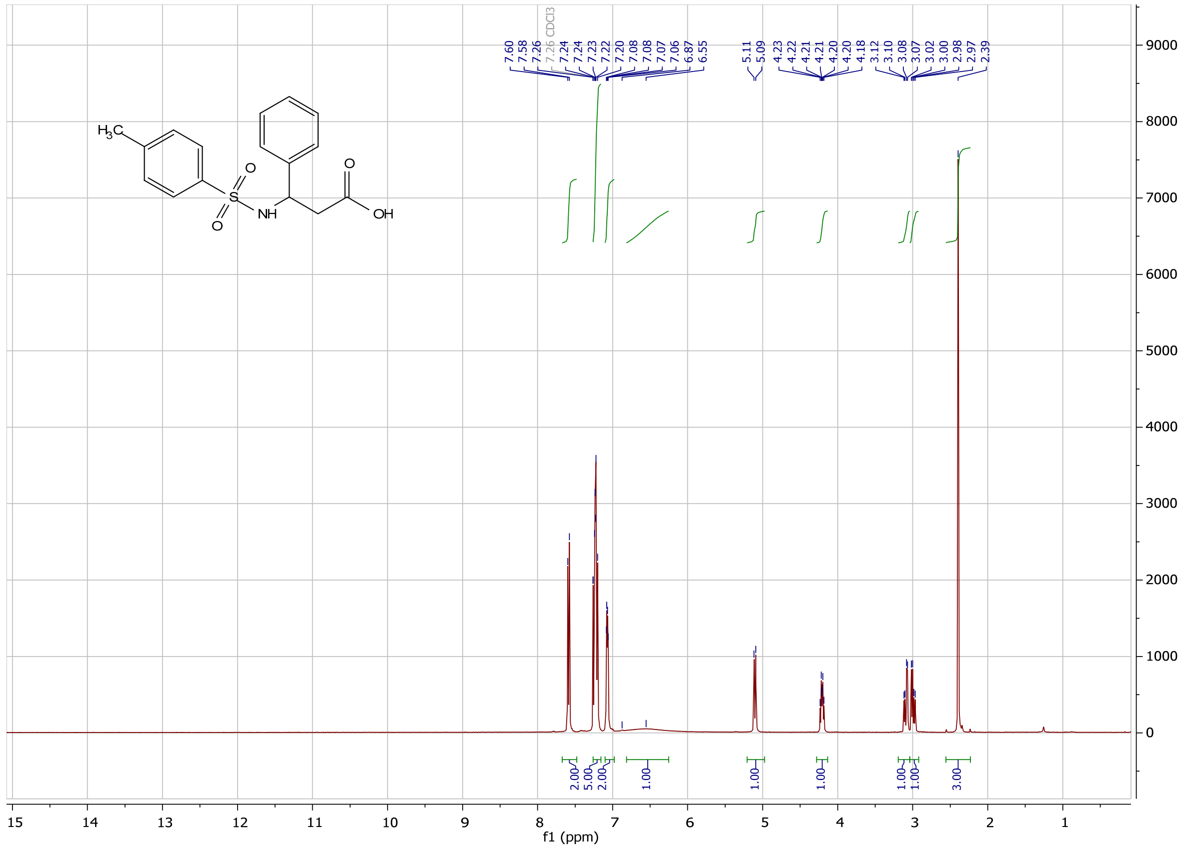

S1. ^{1}H NMR spectra of 3-(4-methylbenzene-1-sulfonamido)-3-phenylpropanoic acid (**2**)

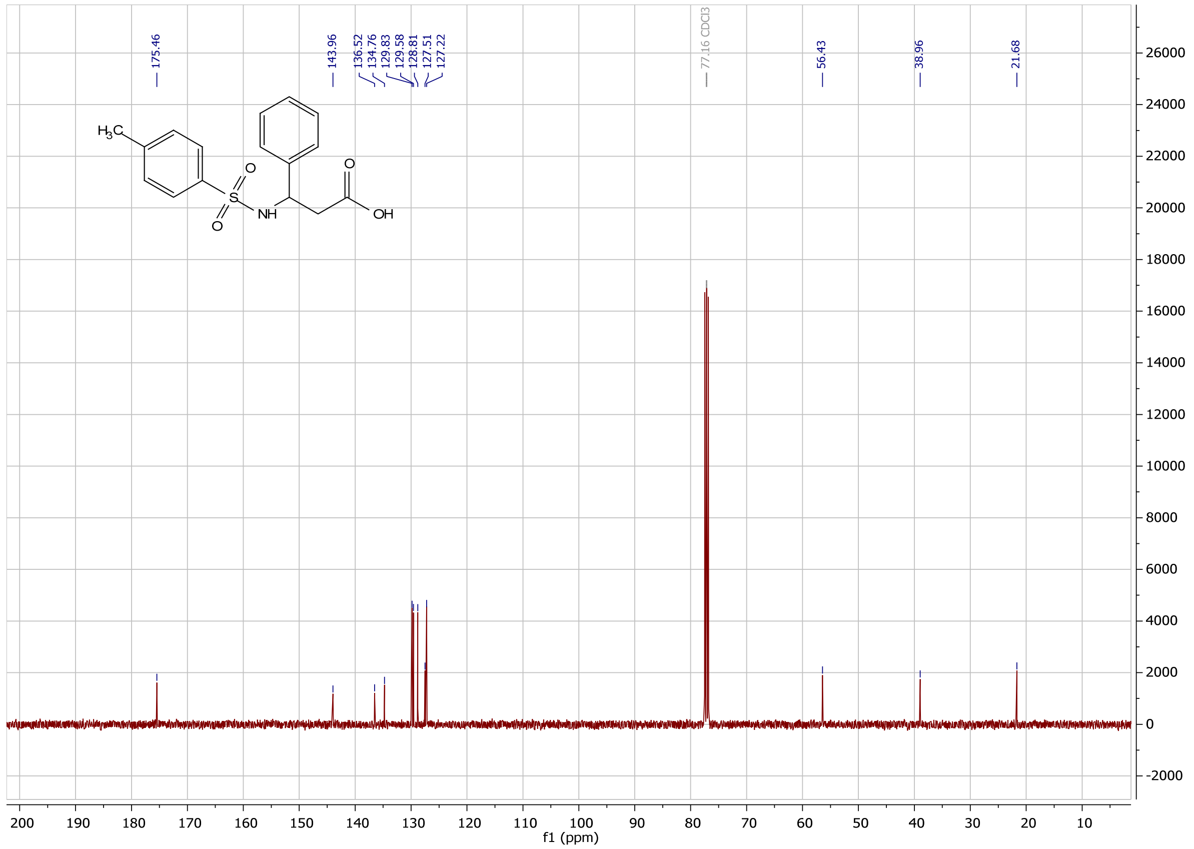

S2.^{13}C NMR spectra of 3-(4-methylbenzene-1-sulfonamido)-3-phenylpropanoic acid (**2**)

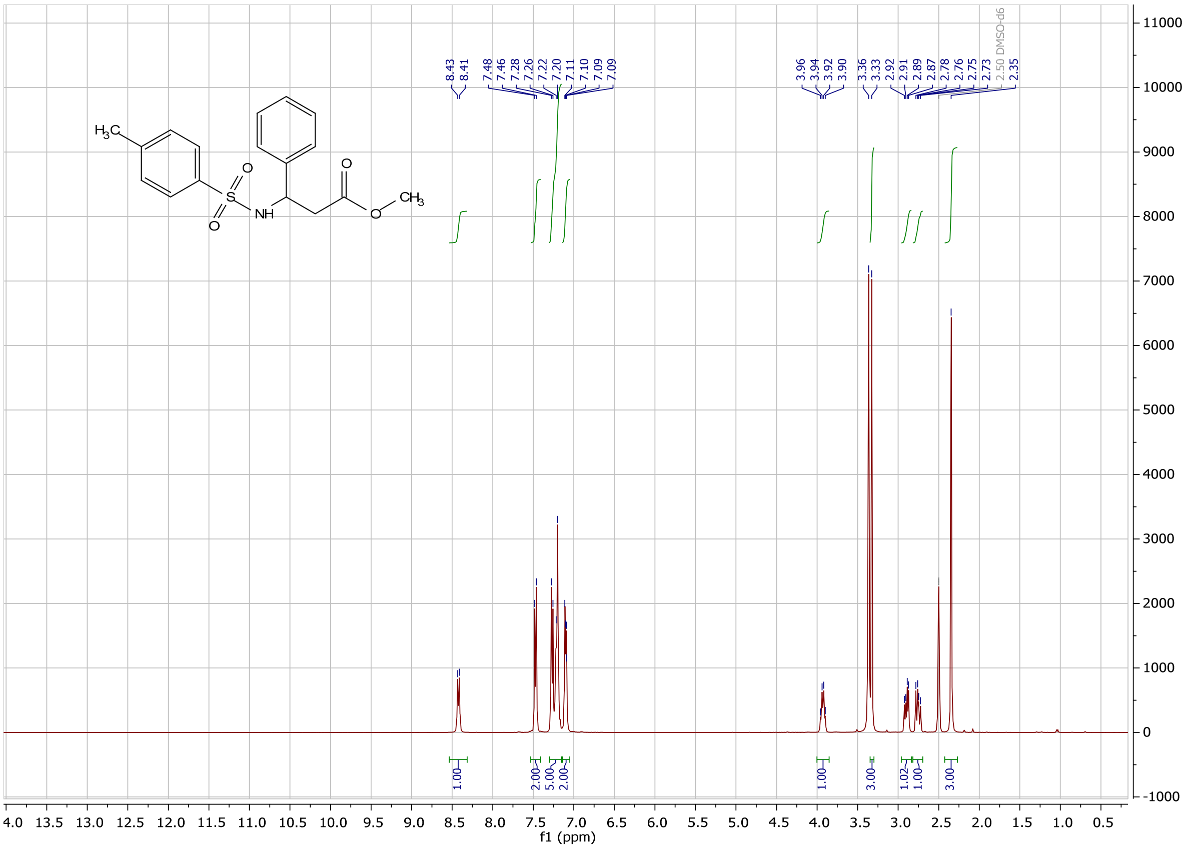

S3. ^{1}H NMR spectra of methyl 3-(4-methylbenzene-1-sulfonamido)-3-phenylpropanoate (**3**)

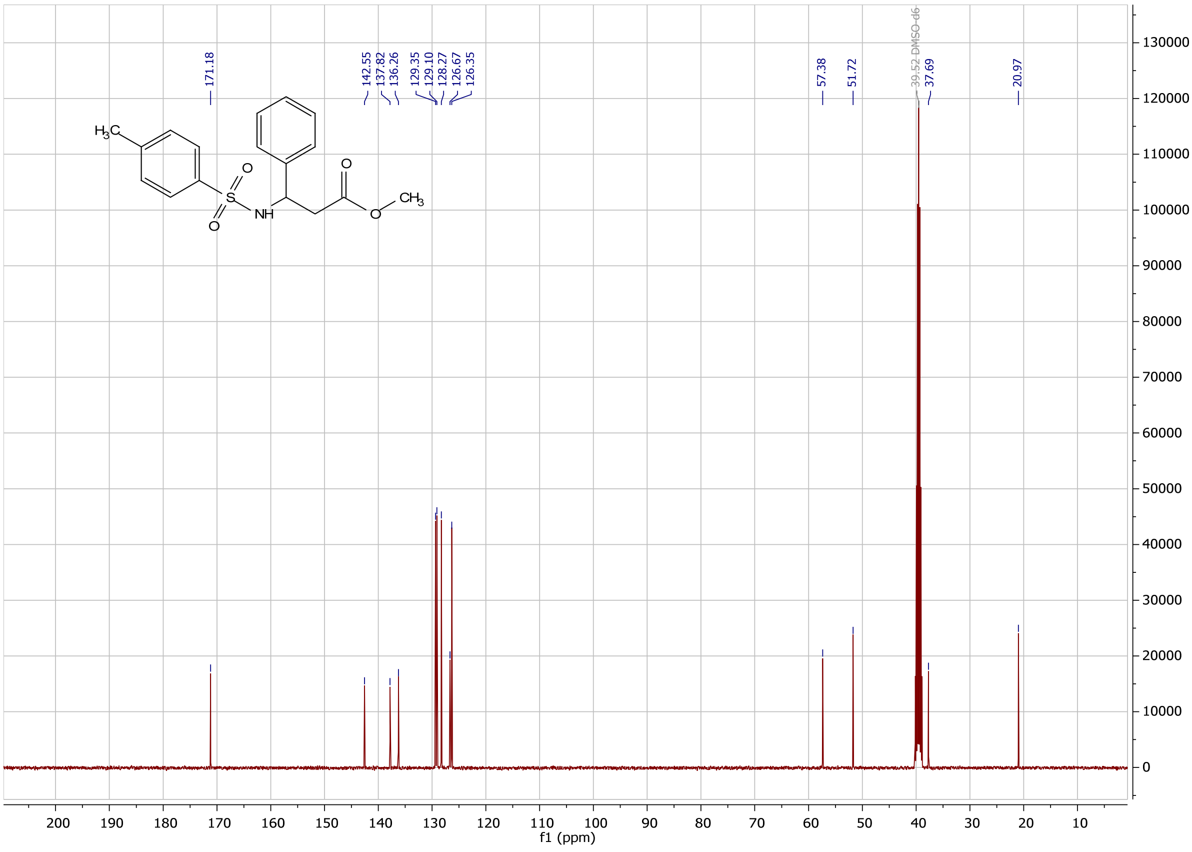

S4.^{13}C NMR spectra of methyl 3-(4-methylbenzene-1-sulfonamido)-3-phenylpropanoate (**3**)

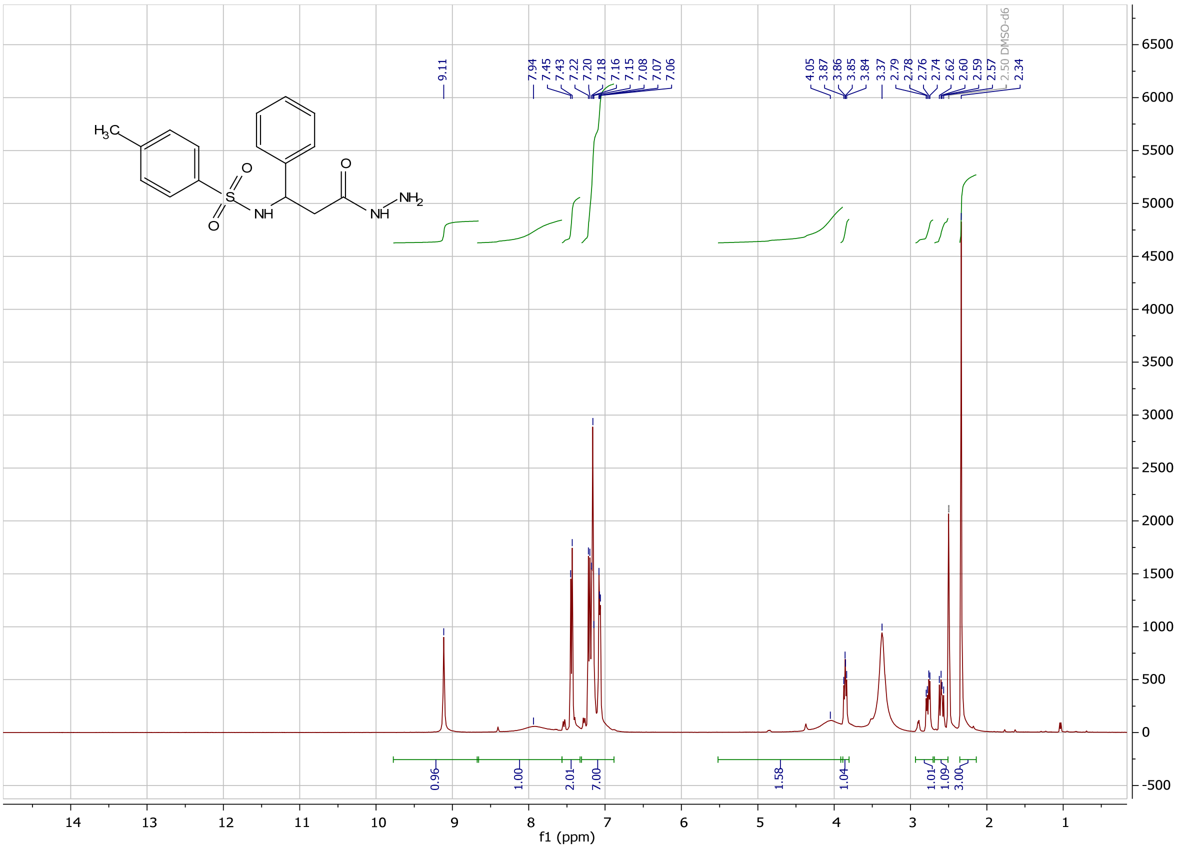

S5. ^{1}H NMR spectra of *N*-[3-hydrazinyl-3-oxo-1-phenylpropyl]-4-methylbenzene-1-sulfonamide (**4**)

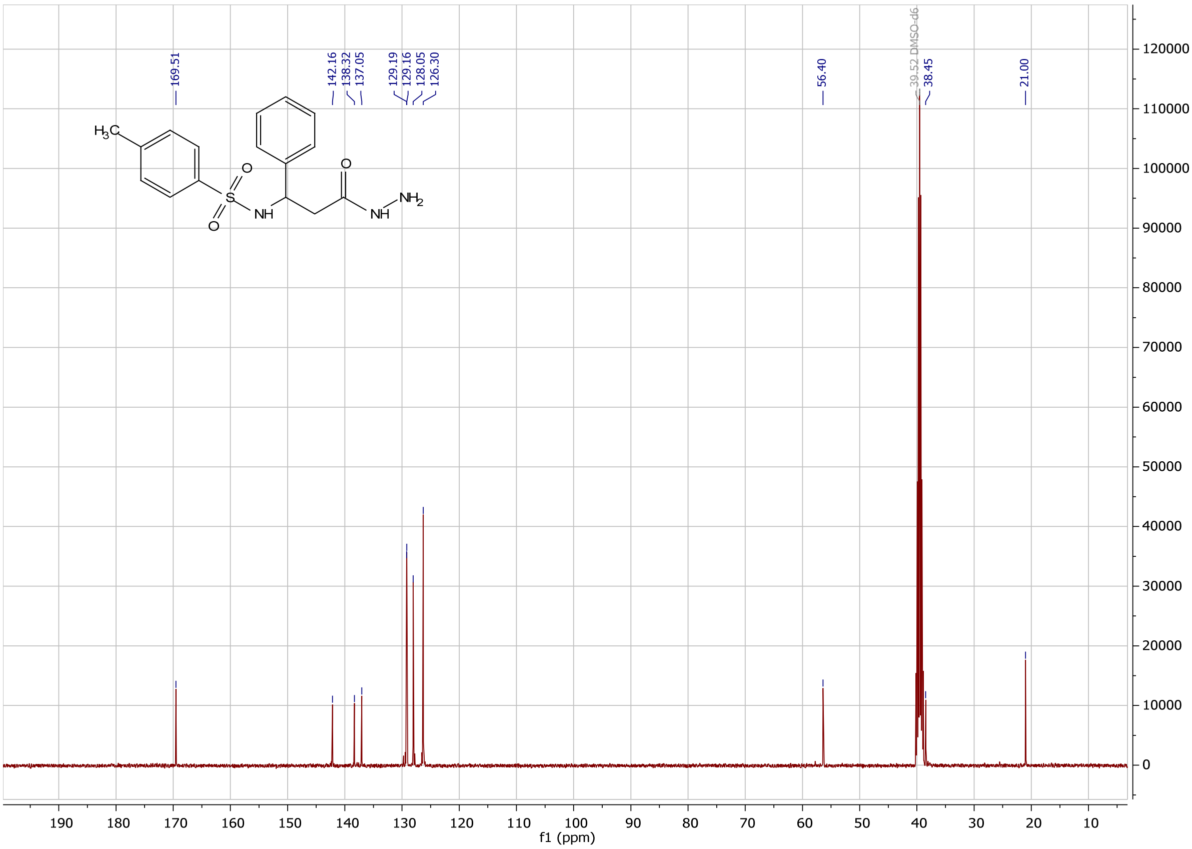

S6.^{13}C NMR spectra of *N*-[3-hydrazinyl-3-oxo-1-phenylpropyl]-4-methylbenzene-1-sulfonamide (**4**)

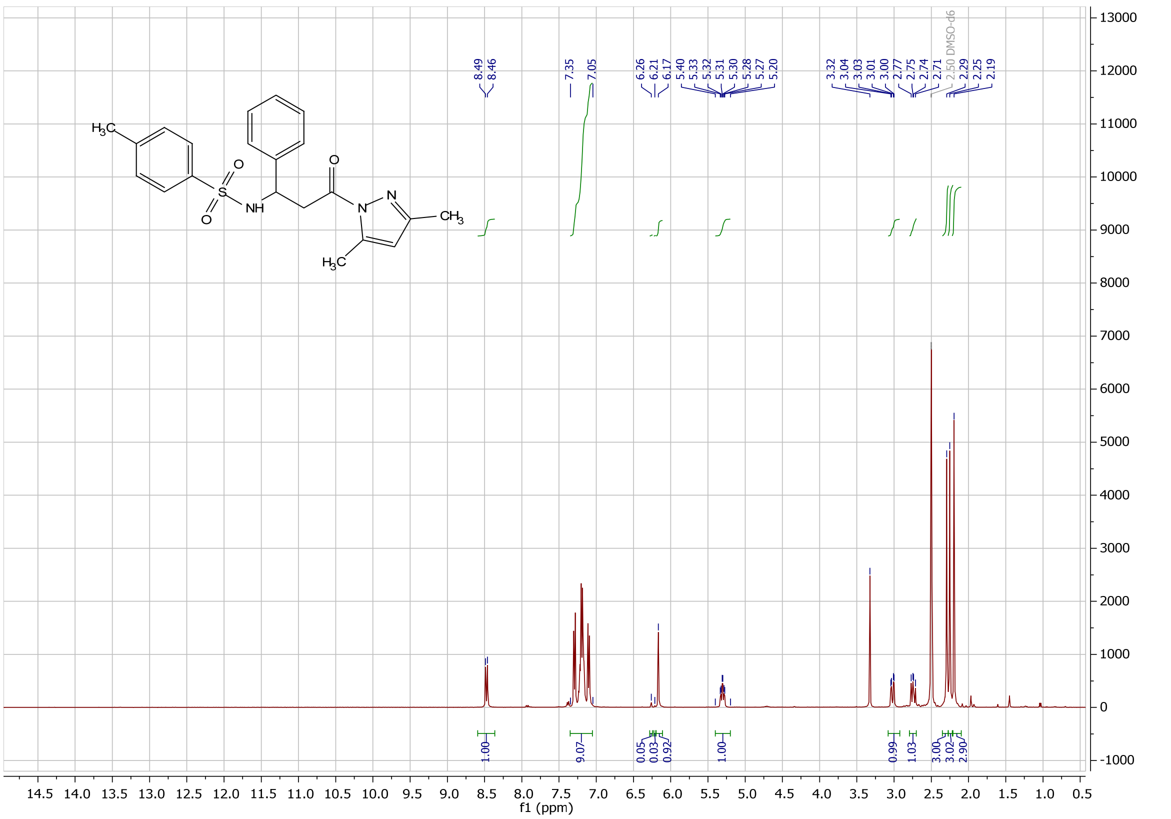

S7. ^{1}H NMR spectra of *N*-[3-(3,5-dimethyl-1*H*-pyrazol-1-yl)-3-oxo-1-phenylpropyl]-4-methylbenzenesulfonamide (**5**)

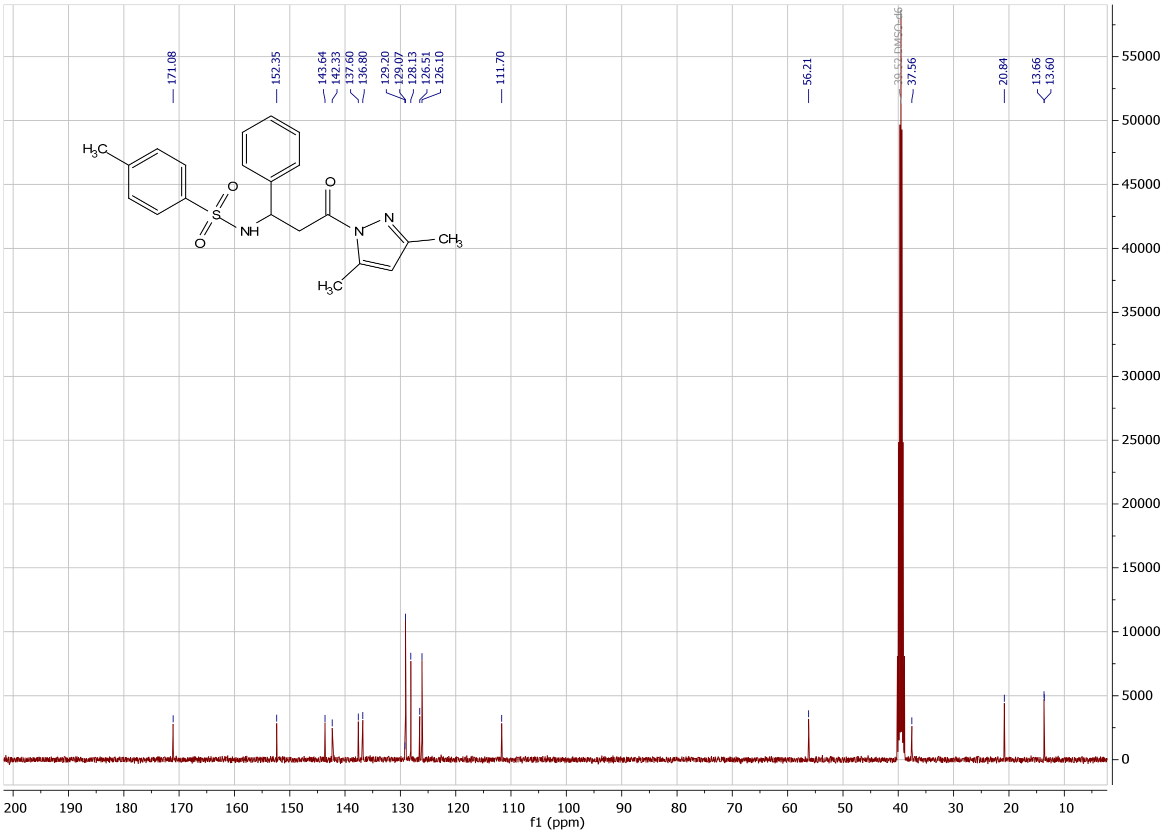

S8. ^{13}C NMR spectra of *N*-[3-(3,5-dimethyl-1*H*-pyrazol-1-yl)-3-oxo-1-phenylpropyl]-4-methylbenzenesulfonamide (**5**)

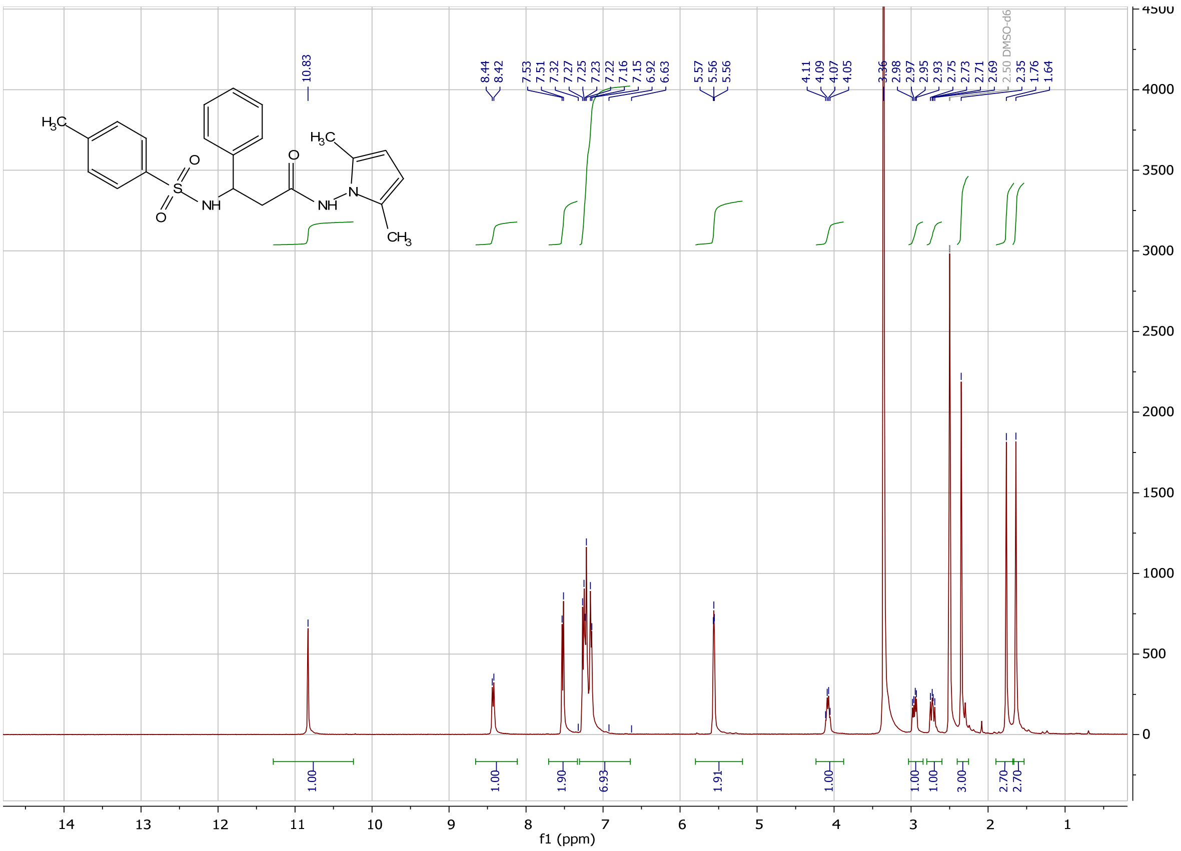

S9. ^{1}H NMR spectra of *N*-(2,5-dimethyl-1*H*-pyrrol-1-yl)-3-[(4-methylphenyl)sulfonamido]-3-phenylpropanamide (**6**)

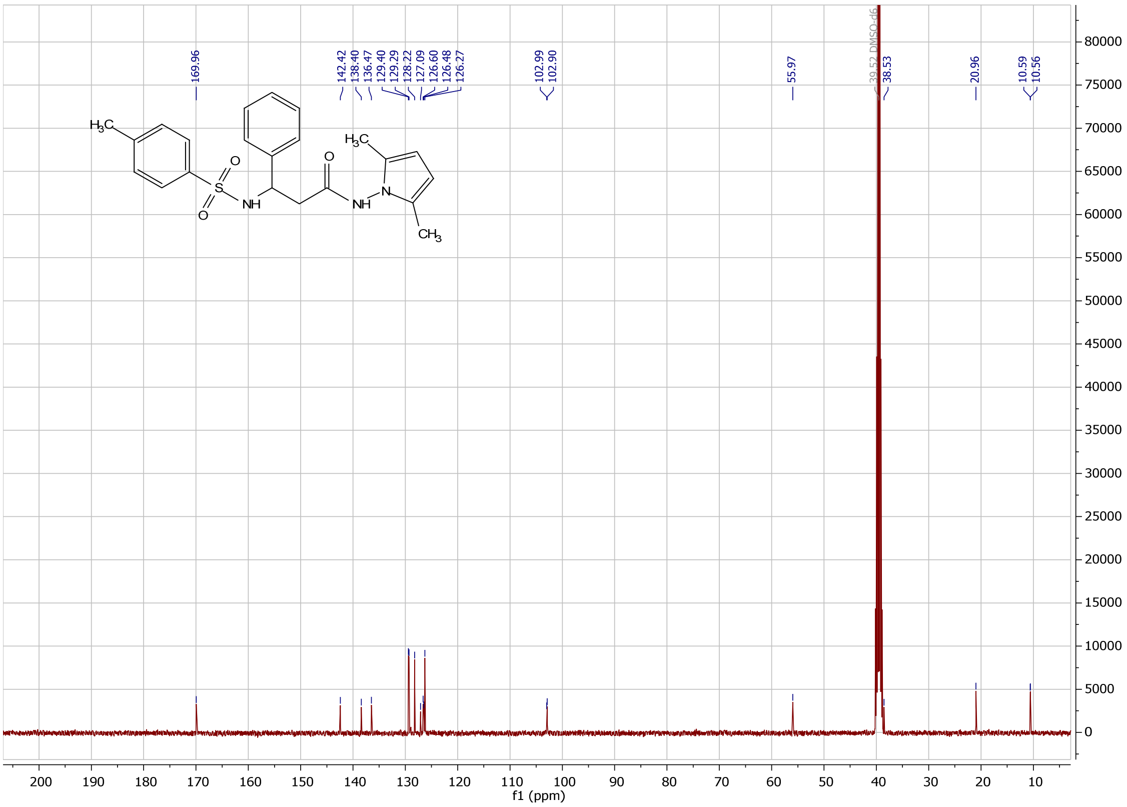

S10.^{13}C NMR spectra of *N*-(2,5-dimethyl-1*H*-pyrrol-1-yl)-3-[(4-methylphenyl)sulfonamido]-3-phenylpropanamide (**6**)

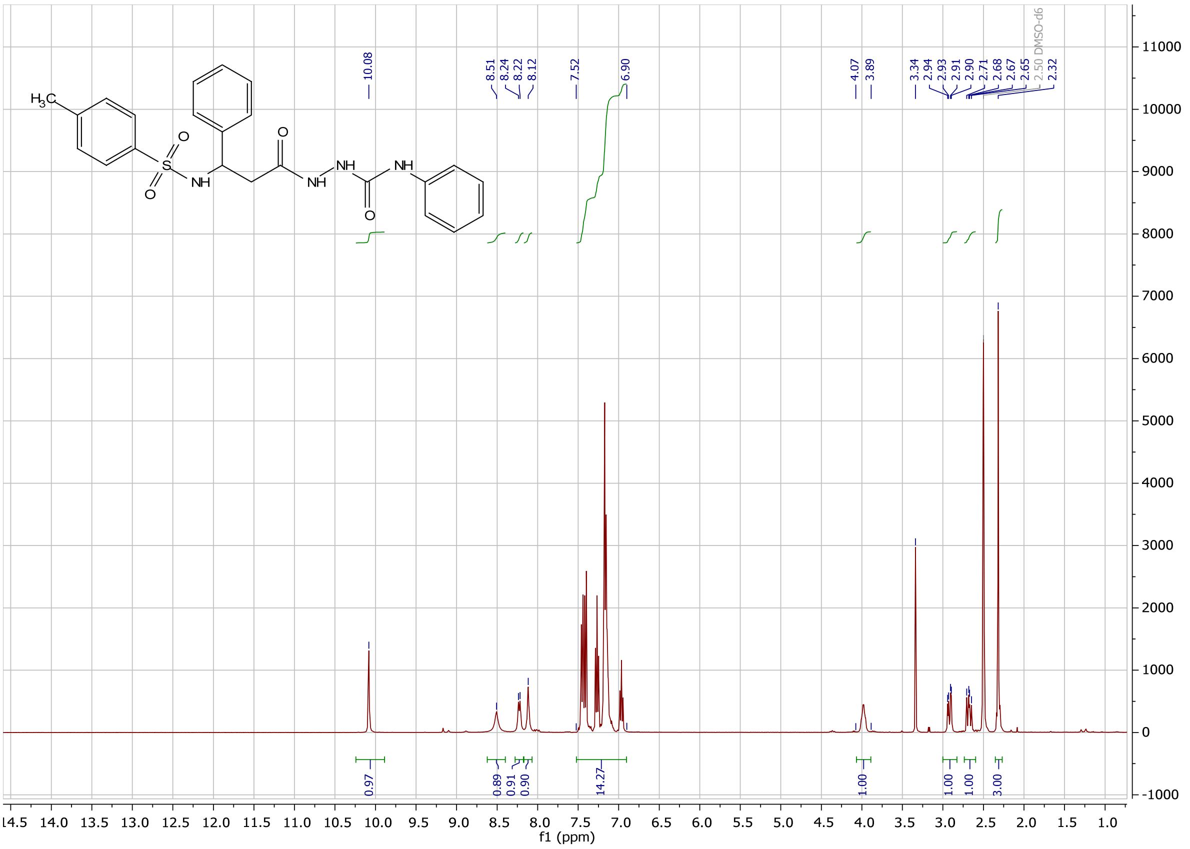

S11. ^{1}H NMR spectra of 2-[3-[(4-methylphenyl)sulfonamido]-3-phenylpropanoyl]-*N*-phenylhydrazine-1-carboxamide (**7a**)

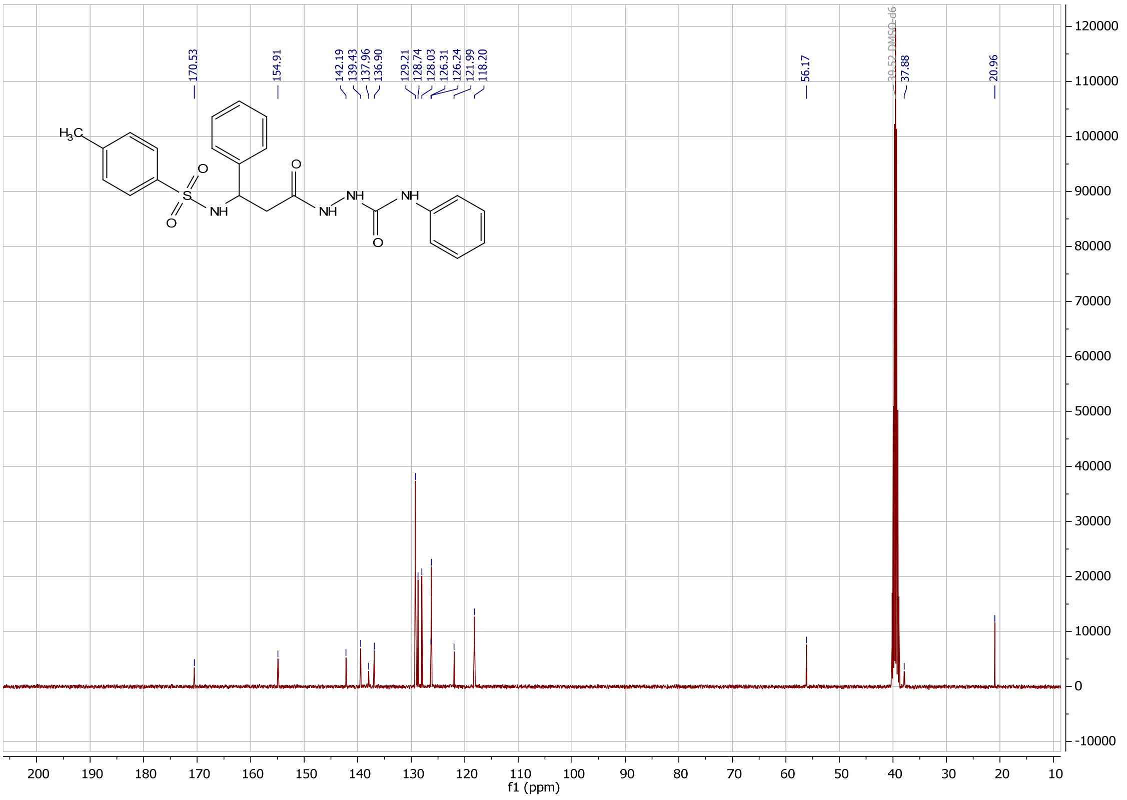

S12. ^{13}C NMR spectra of 2-[3-[(4-methylphenyl)sulfonamido]-3-phenylpropanoyl]-*N*-phenylhydrazine-1-carboxamide (**7a**)

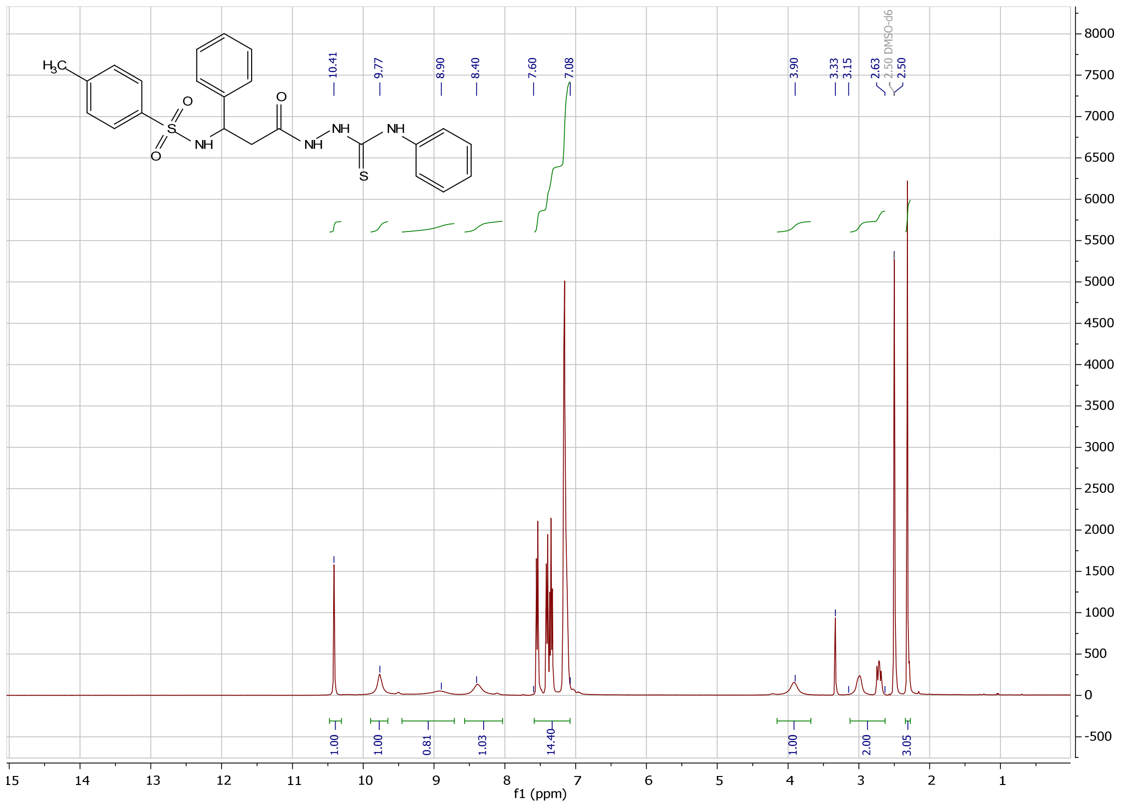

S13. ^{1}H NMR spectra of 2-[3-[(4-methylphenyl)sulfonamido]-3-phenylpropanoyl]-*N*-phenylhydrazine-1-carbothioamide (**7b**)

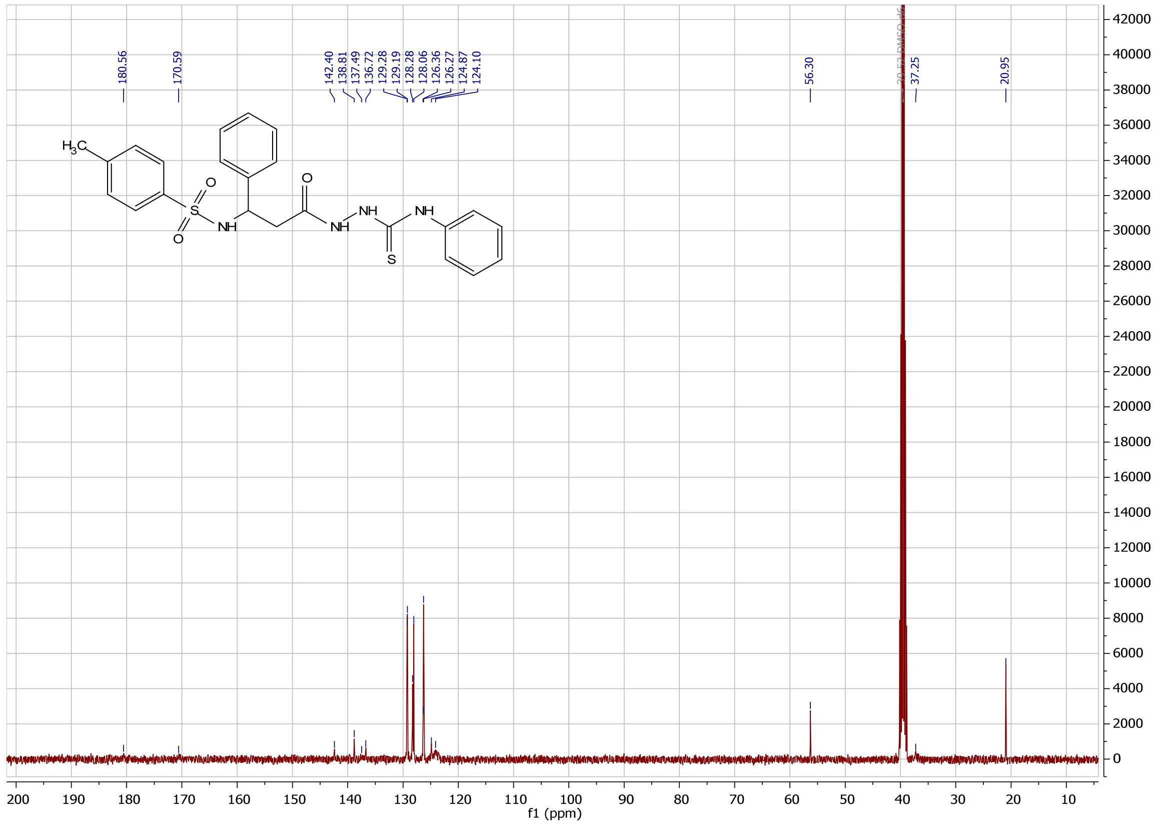

S14. ^{13}C NMR spectra of 2-[3-[(4-methylphenyl)sulfonamido]-3-phenylpropanoyl]-*N*-phenylhydrazine-1-carbothioamide (**7b**)

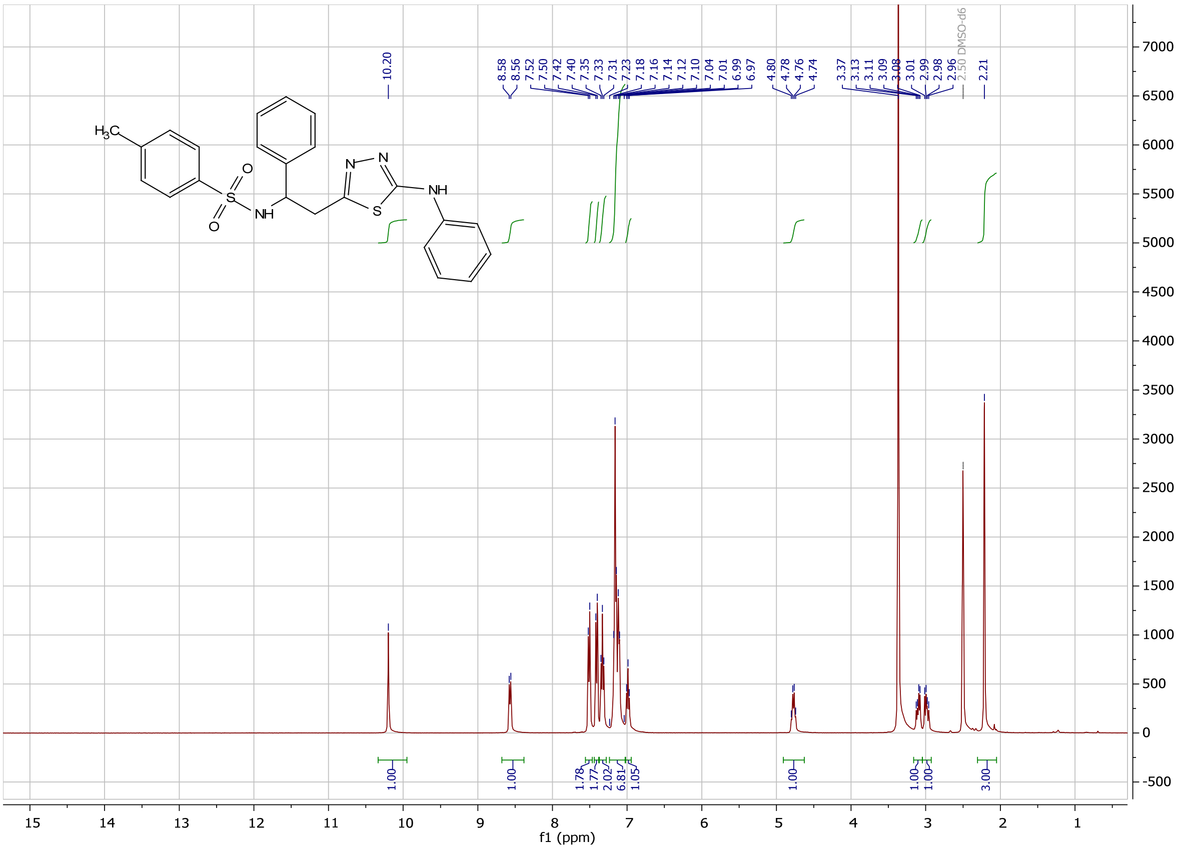

S15. ^{1}H NMR spectra of 4-methyl-*N*-[1-phenyl-2-[5-(phenylamino)-1,3,4-thiadiazol-2-yl]ethyl]benzenesulfonamide (**8**)

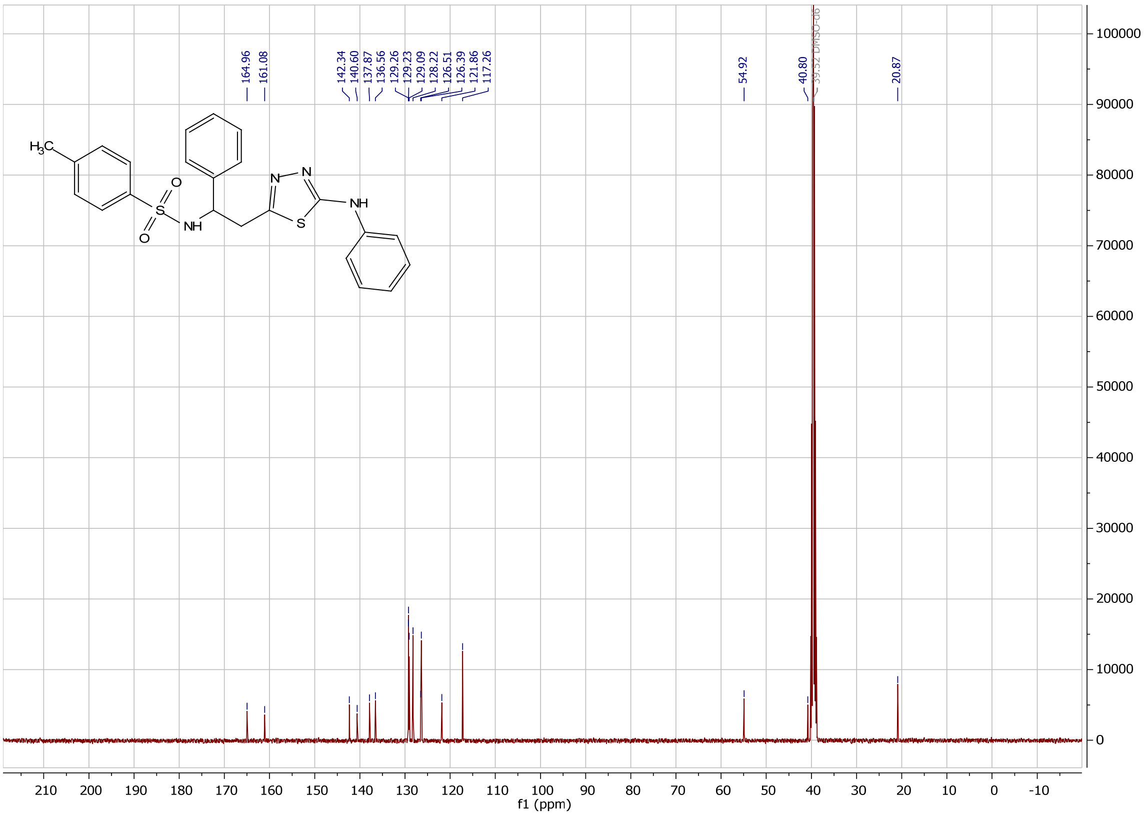

S16. ^{13}C NMR spectra of 4-methyl-*N*-[1-phenyl-2-[5-(phenylamino)-1,3,4-thiadiazol-2-yl]ethyl]benzenesulfonamide (**8**)

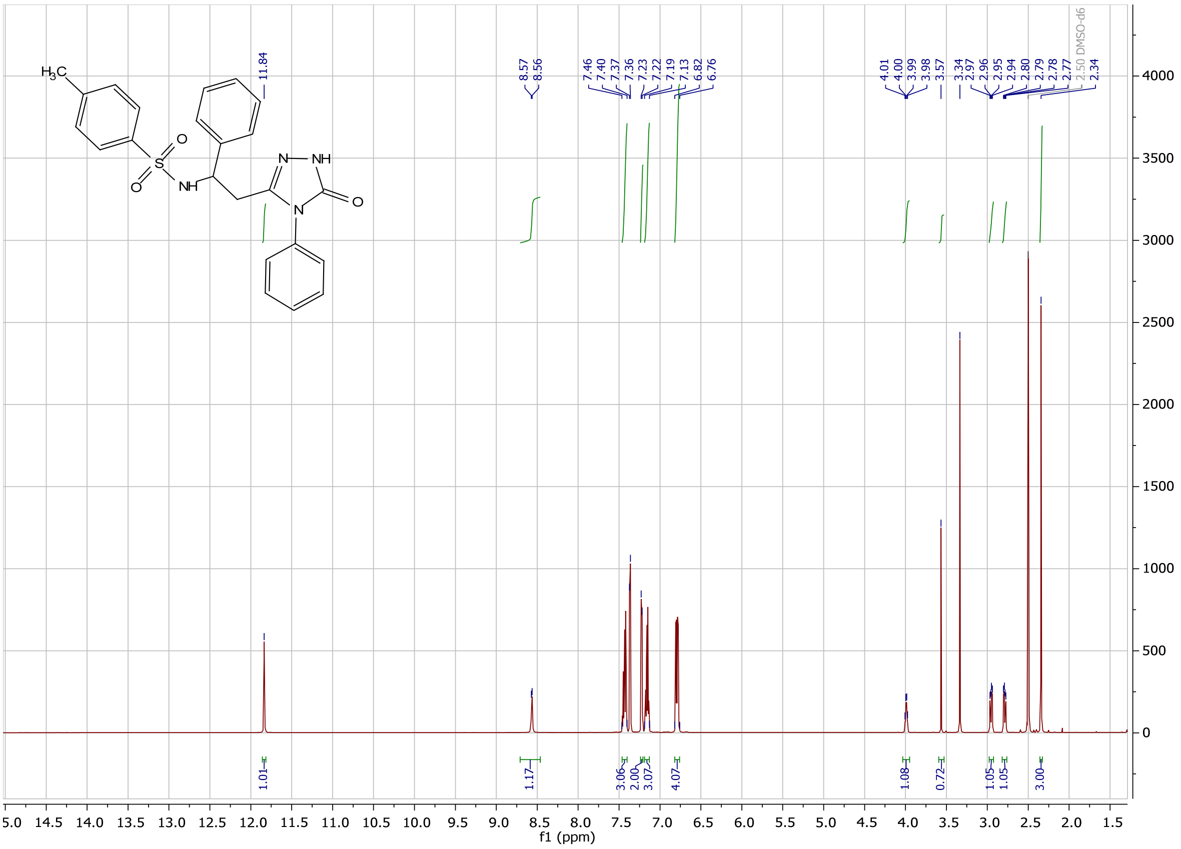

S17. ^{1}H NMR spectra of 4-methyl-*N*-[2-(5-oxo-4-phenyl-4,5-dihydro-1*H*-1,2,4-triazol-3-yl)-1-phenylethyl]benzenesulfonamide (**9a**)

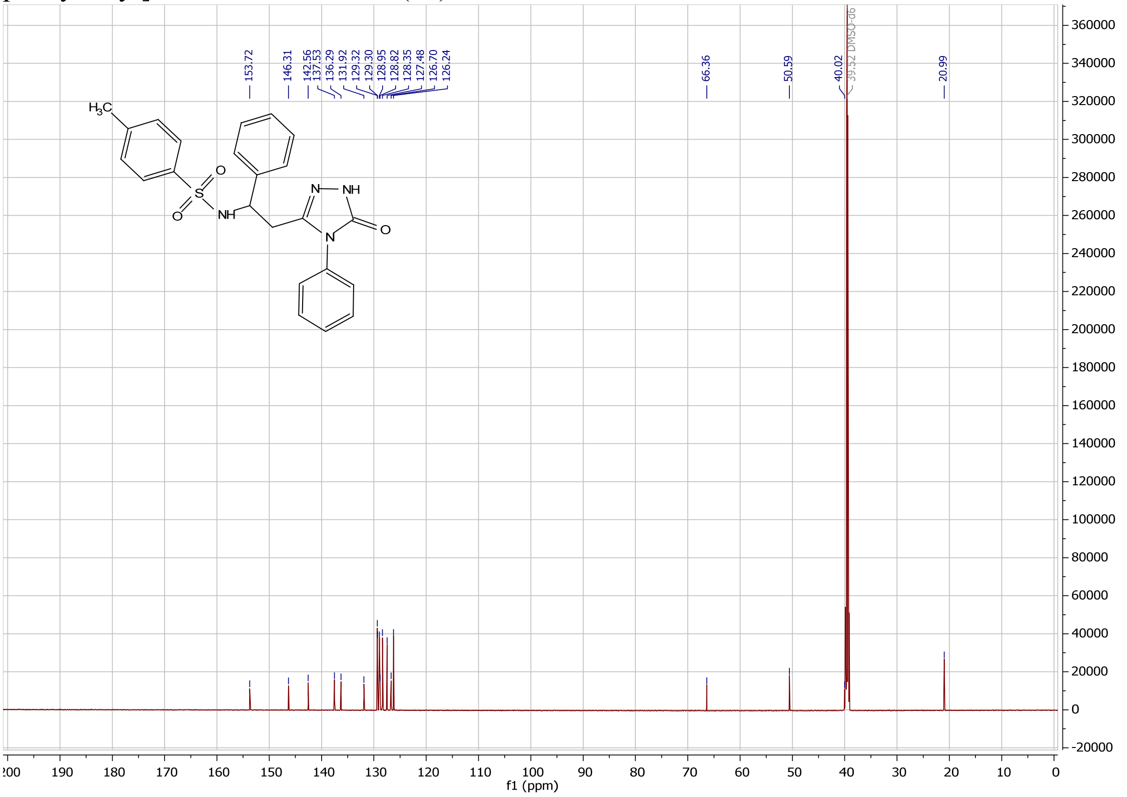

S18. ^{13}C NMR spectra of 4-methyl-*N*-[2-(5-oxo-4-phenyl-4,5-dihydro-1*H*-1,2,4-triazol-3-yl)-1-phenylethyl]benzenesulfonamide (**9a**)

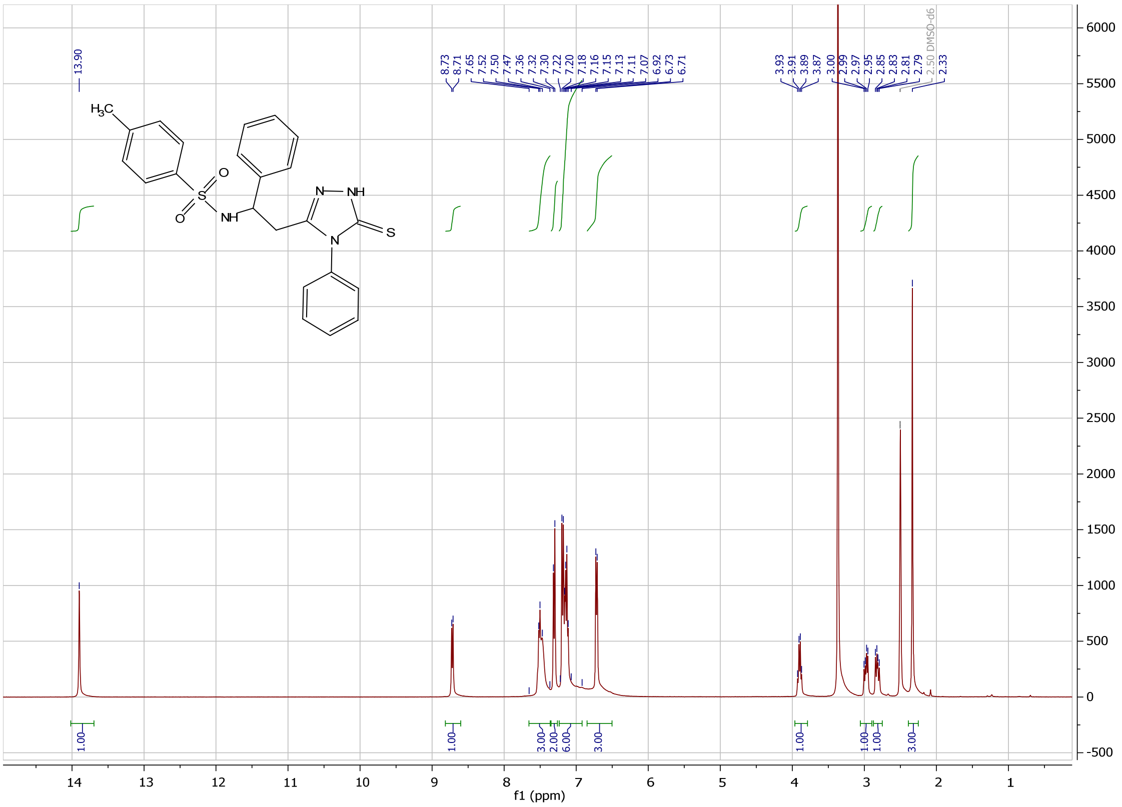

S19. ^{1}H NMR spectra of 4-methyl-*N*-[2-(5-thioxo-4-phenyl-4,5-dihydro-1*H*-1,2,4-triazol-3-yl)-1-phenylethyl]benzenesulfonamide (**9b**)

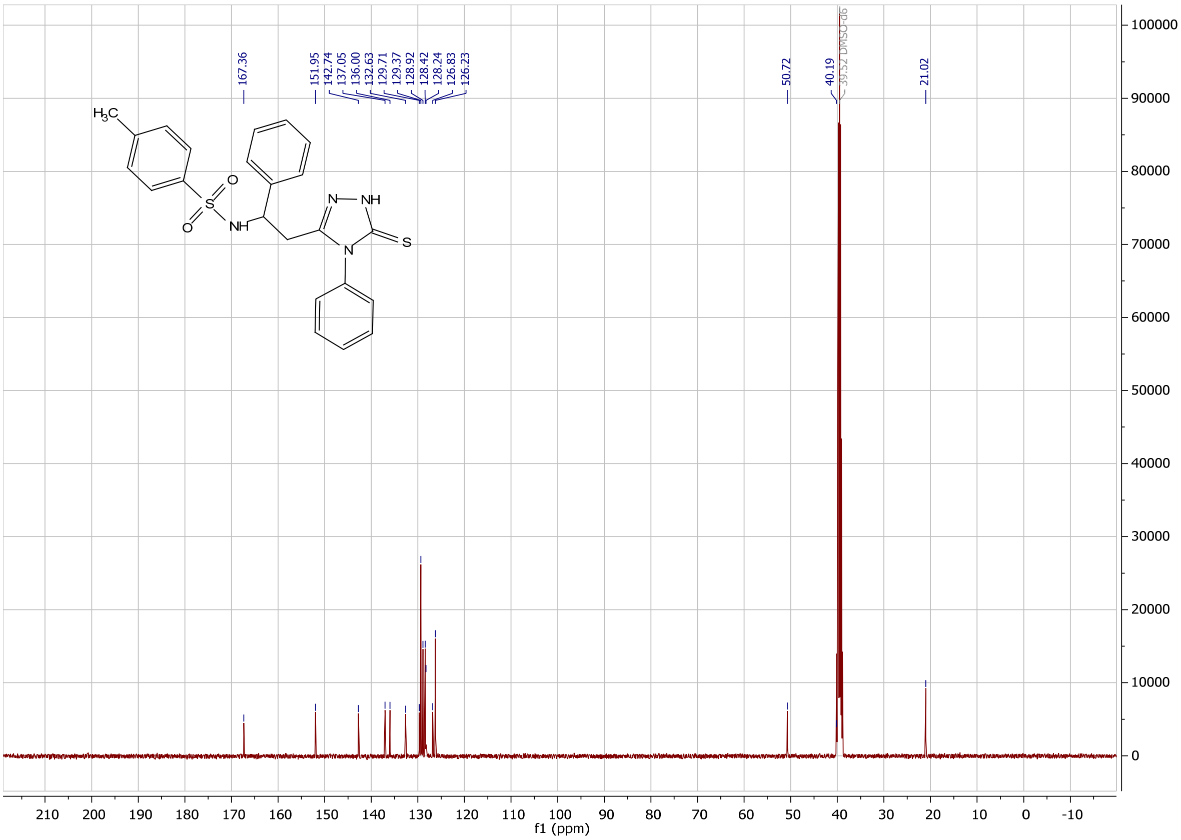

S20. ^{13}C NMR spectra of 4-methyl-*N*-[2-(5-thioxo-4-phenyl-4,5-dihydro-1*H*-1,2,4-triazol-3-yl)-1-phenylethyl]benzenesulfonamide (**9b**)

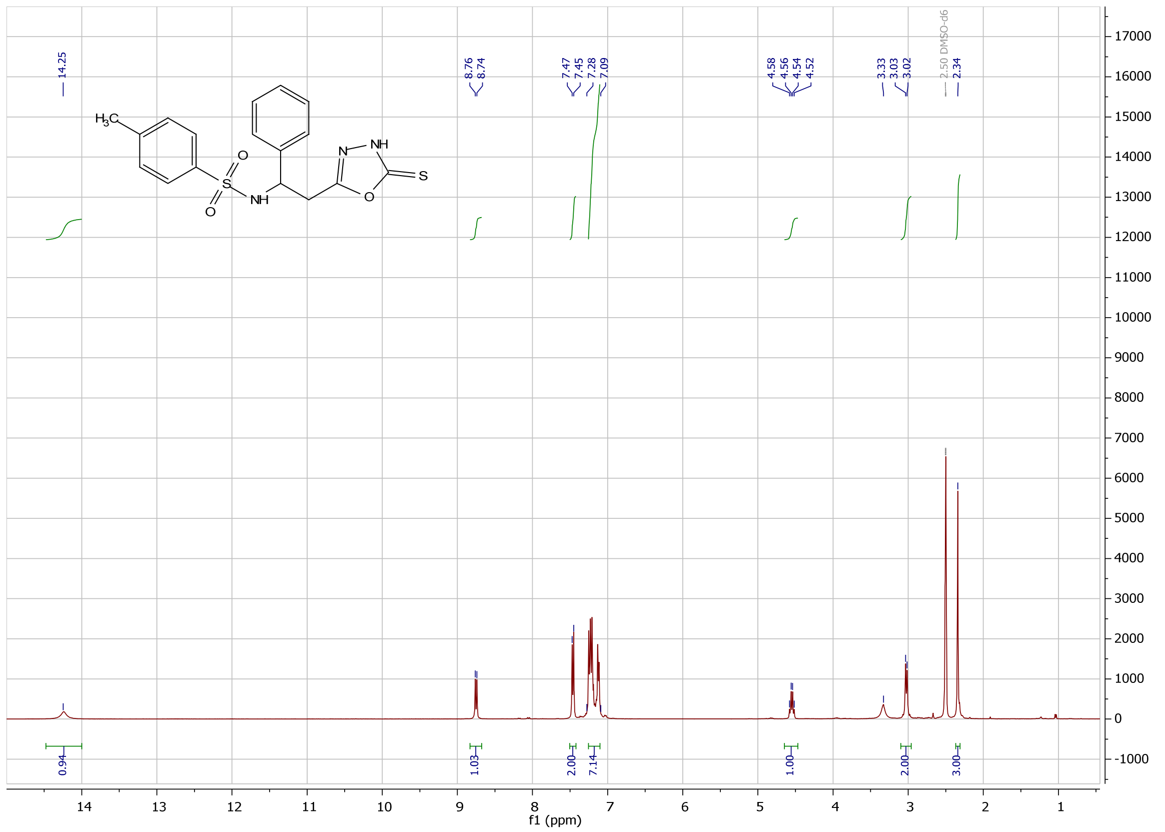

S21. ^{1}H NMR spectra of 4-methyl-*N*-[1-phenyl-2-(5-thioxo-4,5-dihydro-1,3,4-oxadiazol-2-yl)ethyl]benzenesulfonamide (**11**)

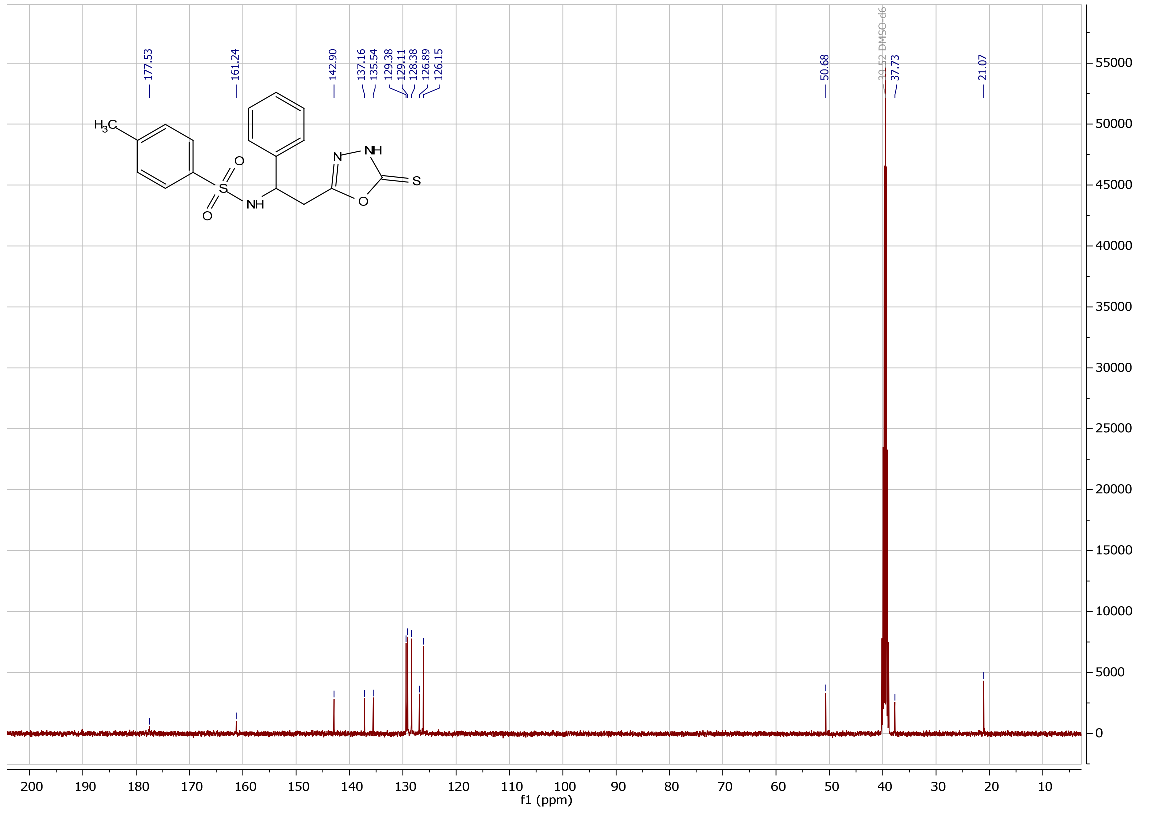

S22. ^{13}C NMR spectra of 4-methyl-*N*-[1-phenyl-2-(5-thioxo-4,5-dihydro-1,3,4-oxadiazol-2-yl)ethyl]benzenesulfonamide (**11**)

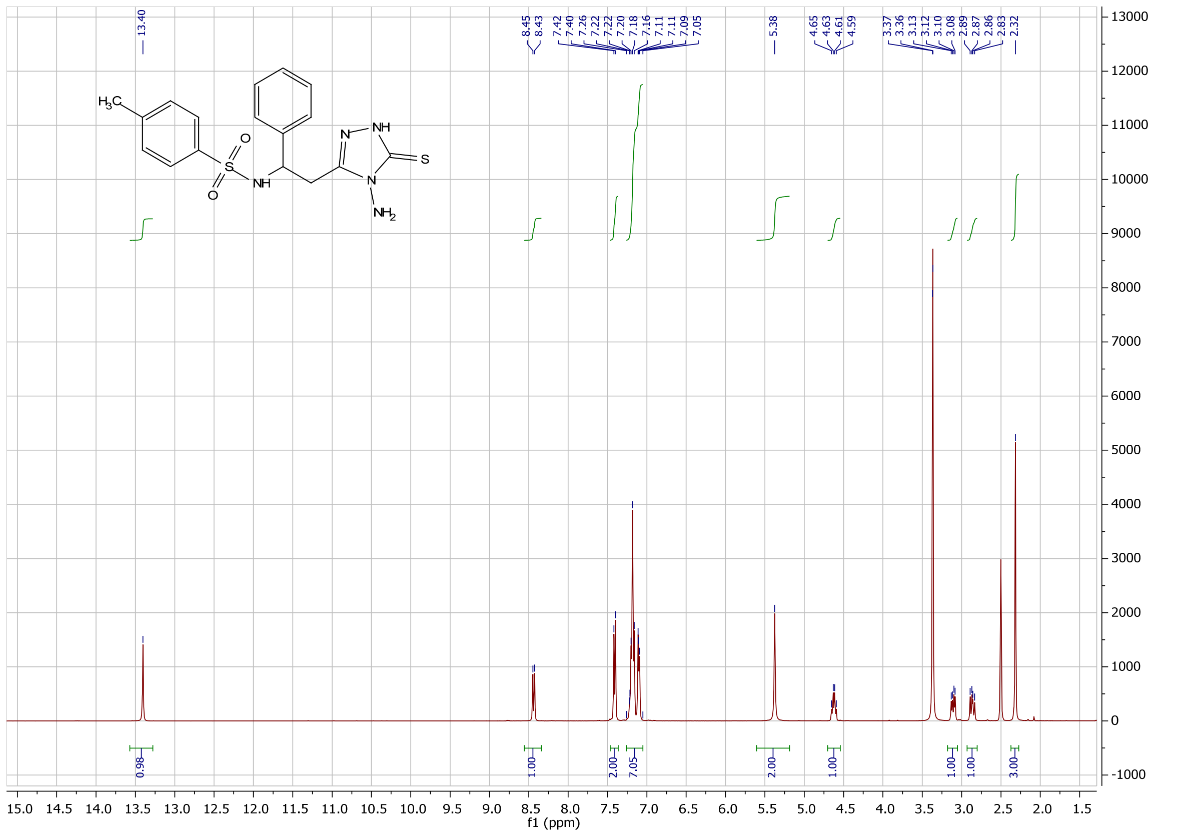

S23. ^{1}H NMR spectra of *N*-[2-(4-amino-5-thioxo-4,5-dihydro-1*H*-1,2,4-triazol-3-yl)-1-phenylethyl]-4-methylbenzenesulfonamide (**12**)

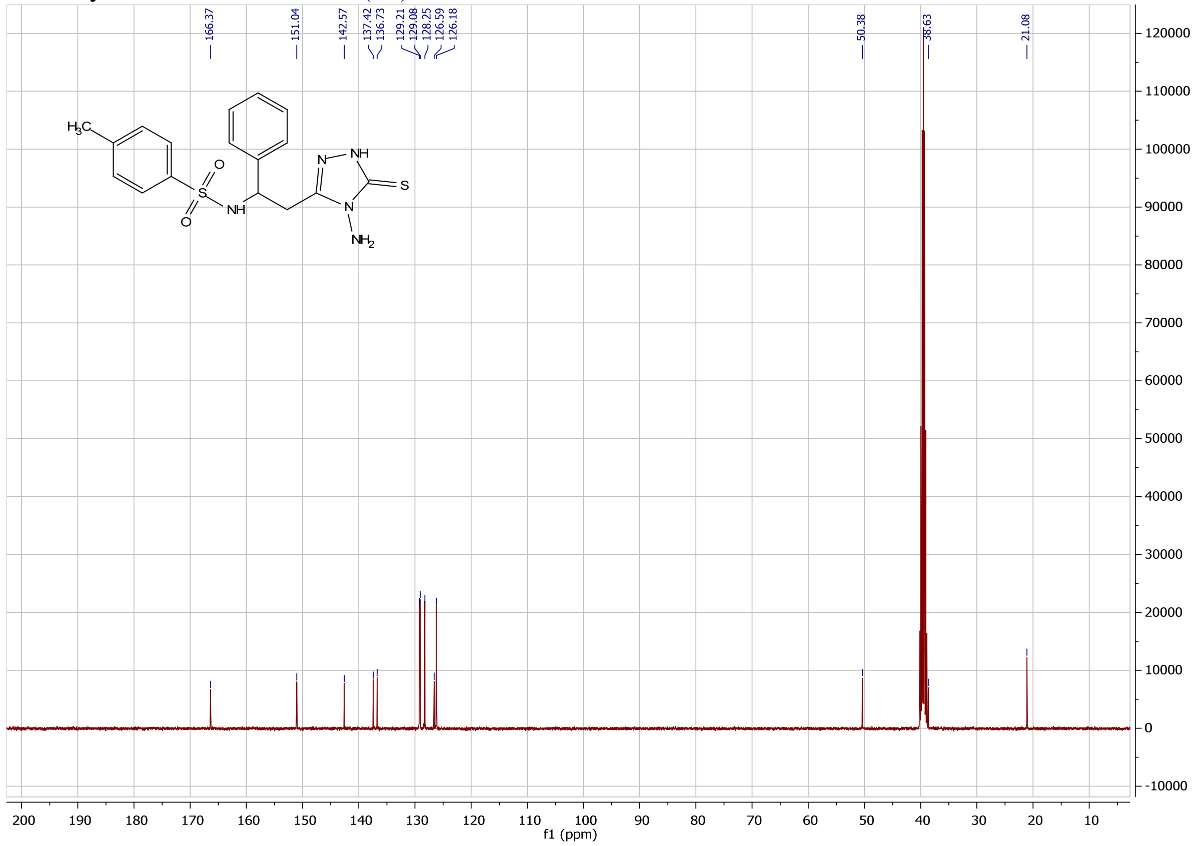

S24. ^{13}C NMR spectra of *N*-[2-(4-amino-5-thioxo-4,5-dihydro-1*H*-1,2,4-triazol-3-yl)-1-phenylethyl]-4-methylbenzenesulfonamide (**12**)

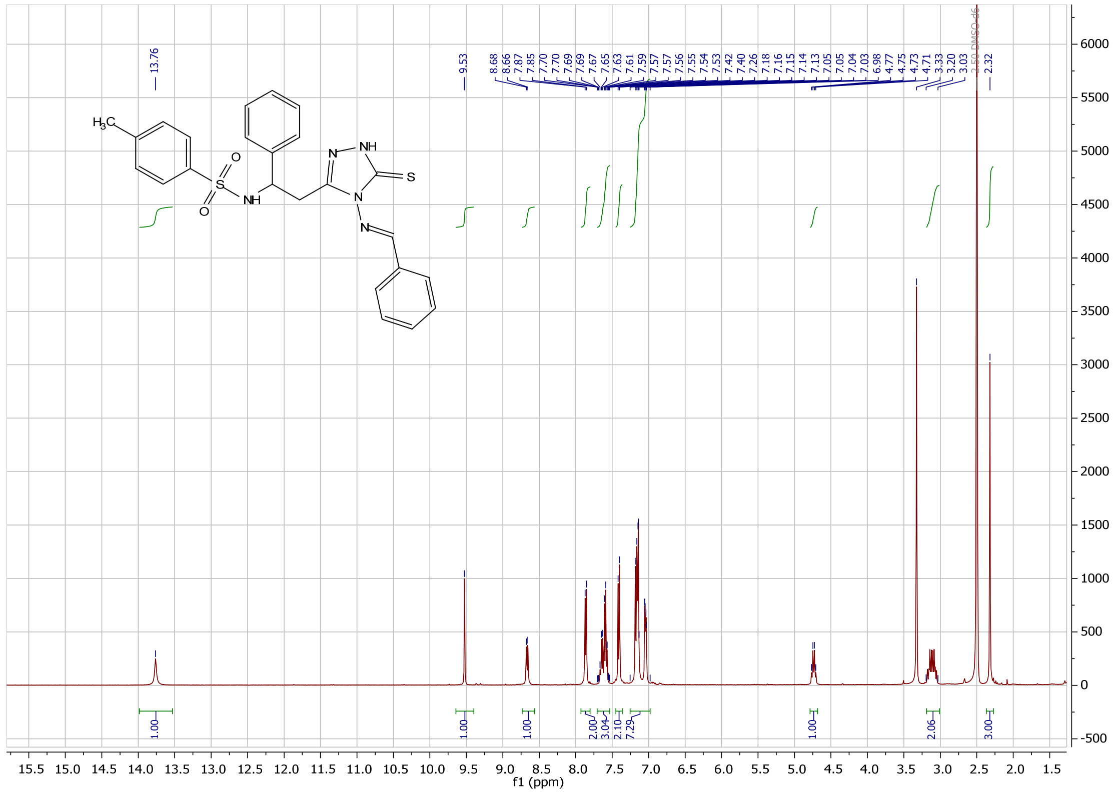

S25. ^{1}H NMR spectra of (*E*)-*N*-[2-[4-(benzylideneamino)-5-thioxo-4,5-dihydro-1*H*-1,2,4-triazol-3-yl]-1-phenylethyl]-4-methylbenzenesulfonamide (**13a**)

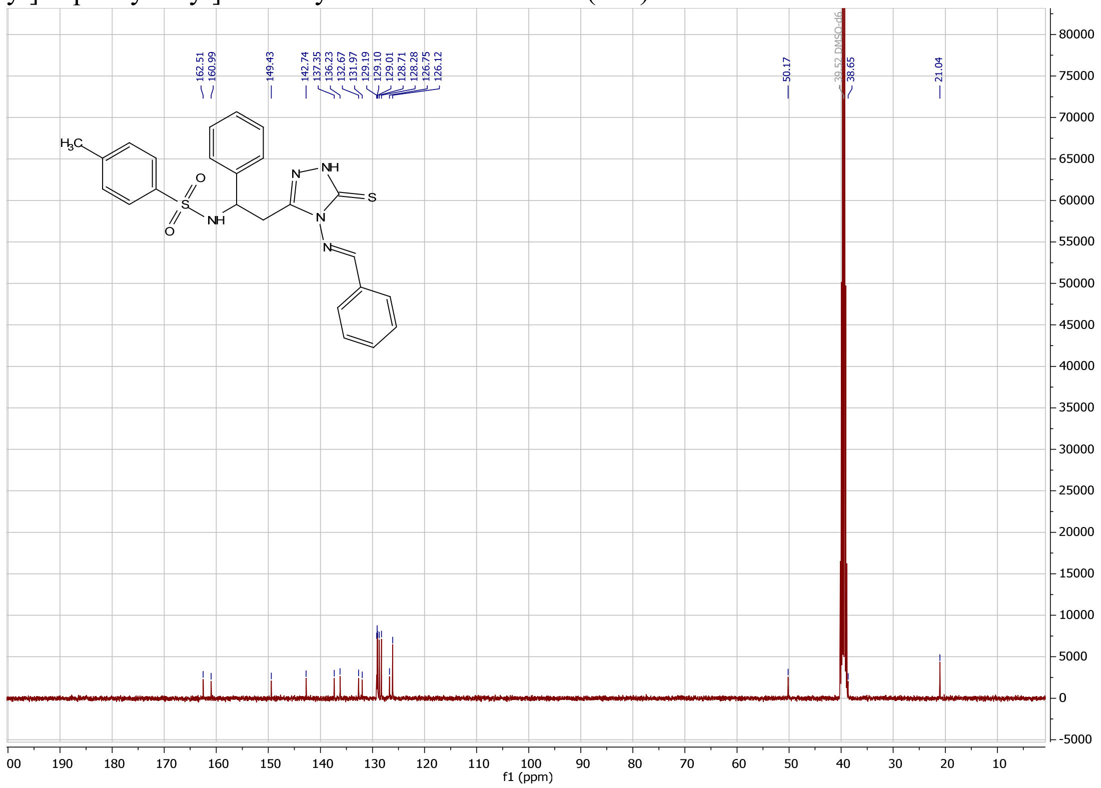

S26. ^{13}C NMR spectra of (*E*)-*N*-[2-[4-(benzylideneamino)-5-thioxo-4,5-dihydro-1*H*-1,2,4-triazol-3-yl]-1-phenylethyl]-4-methylbenzenesulfonamide (**13a**)

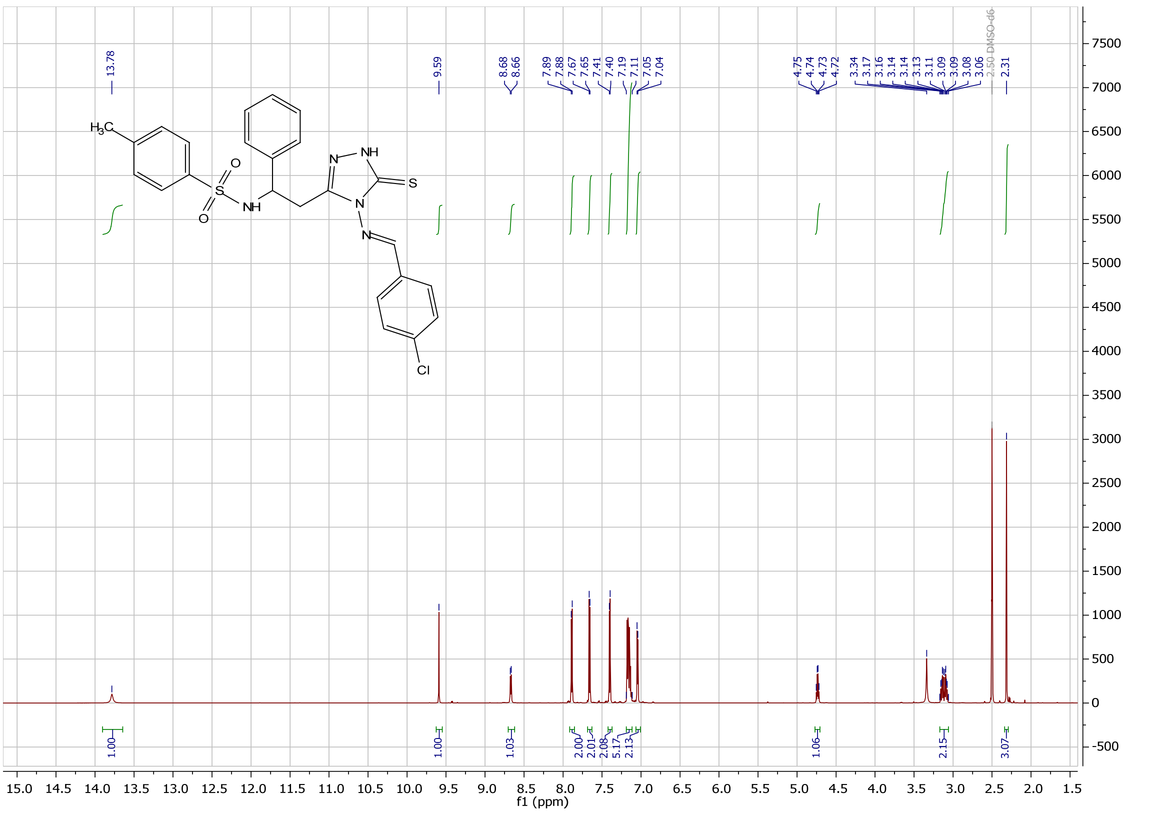

S27. ^{1}H NMR spectra of (*E*)-*N*-{2-[4-[(4-chlorobenzylidene)amino]-5-thioxo-4,5-dihydro-1*H*-1,2,4-triazol-3-yl]-1-phenylethyl}-4-methylbenzenesulfonamide (**13b**)

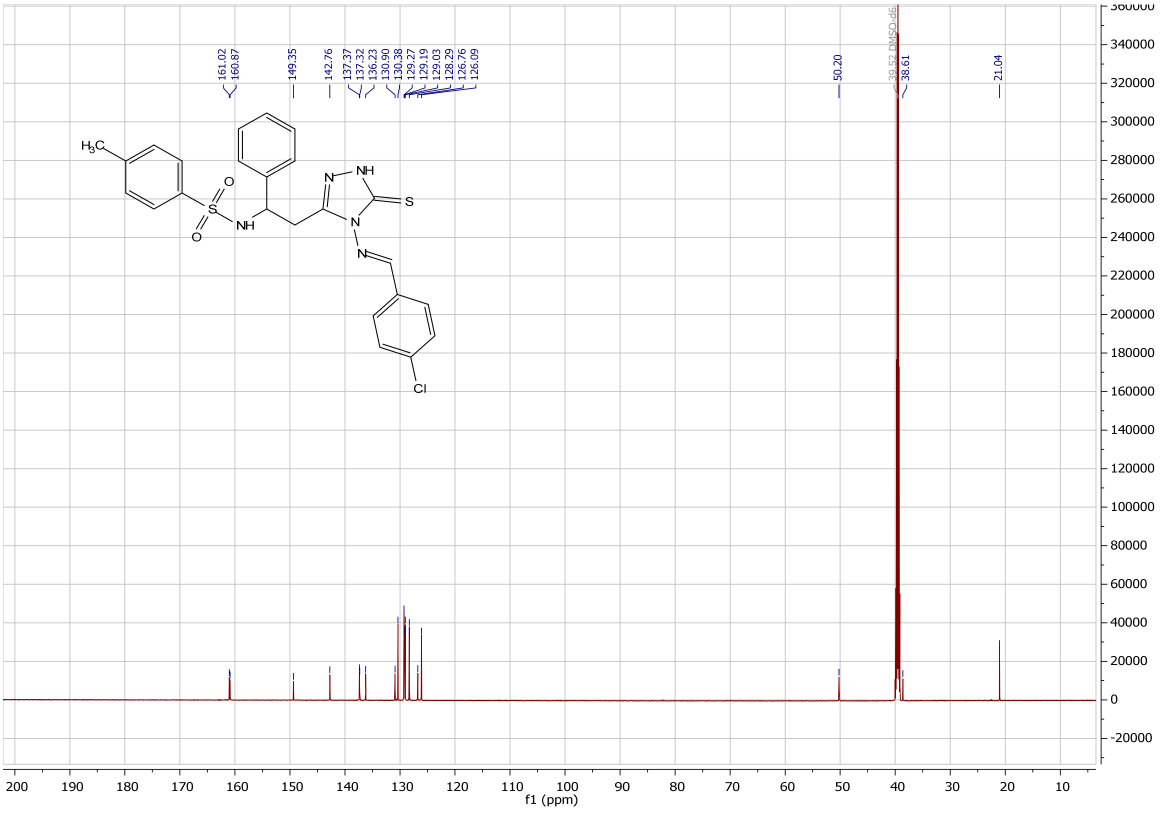

S28. ^{13}C NMR spectra of (*E*)-*N*-{2-[4-[(4-chlorobenzylidene)amino]-5-thioxo-4,5-dihydro-1*H*-1,2,4-triazol-3-yl]-1-phenylethyl}-4-methylbenzenesulfonamide (**13b**)

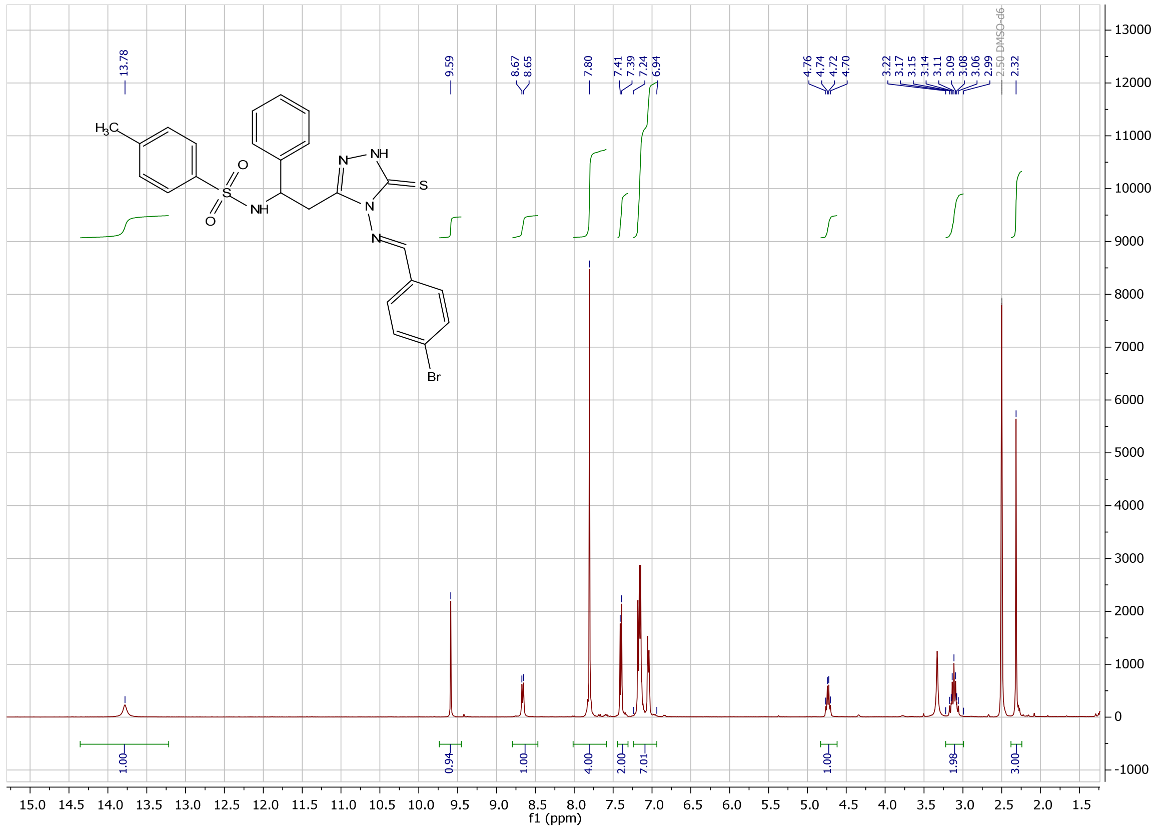

S29. ^{1}H NMR spectra of (*E*)-*N*-{2-[4-[(4-bromobenzylidene)amino]-5-thioxo-4,5-dihydro-1*H*-1,2,4-triazol-3-yl]-1-phenylethyl}-4-methylbenzenesulfonamide (**13c**)

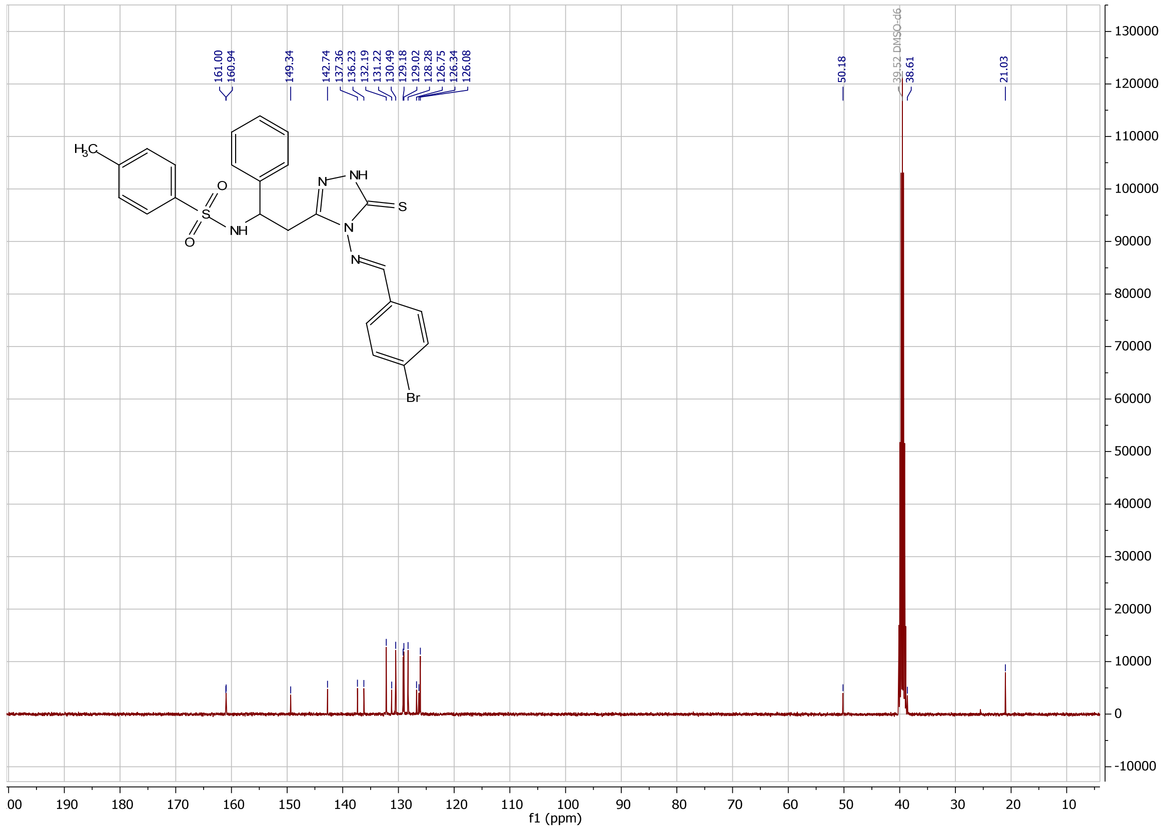

S30. ^{1}H NMR spectra of (*E*)-*N*-{2-[4-[(4-bromobenzylidene)amino]-5-thioxo-4,5-dihydro-1*H*-1,2,4-triazol-3-yl]-1-phenylethyl}-4-methylbenzenesulfonamide (**13c**)

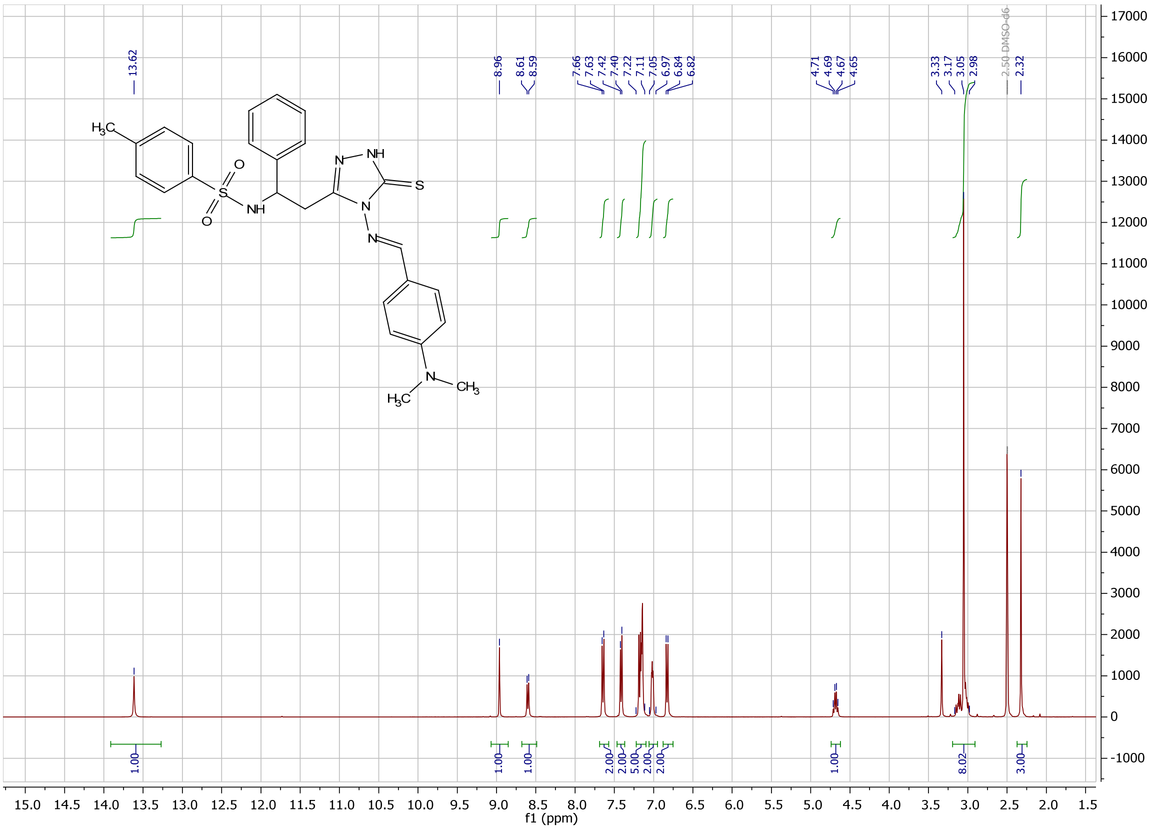

S31. ^{1}H NMR spectra of (*E*)-*N*-{2-[4-[[4-(dimethylamino)benzylidene]amino]-5-thioxo-4,5-dihydro-1*H*-1,2,4-triazol-3-yl]-1-phenylethyl}-4-methylbenzenesulfonamide (**13d**)

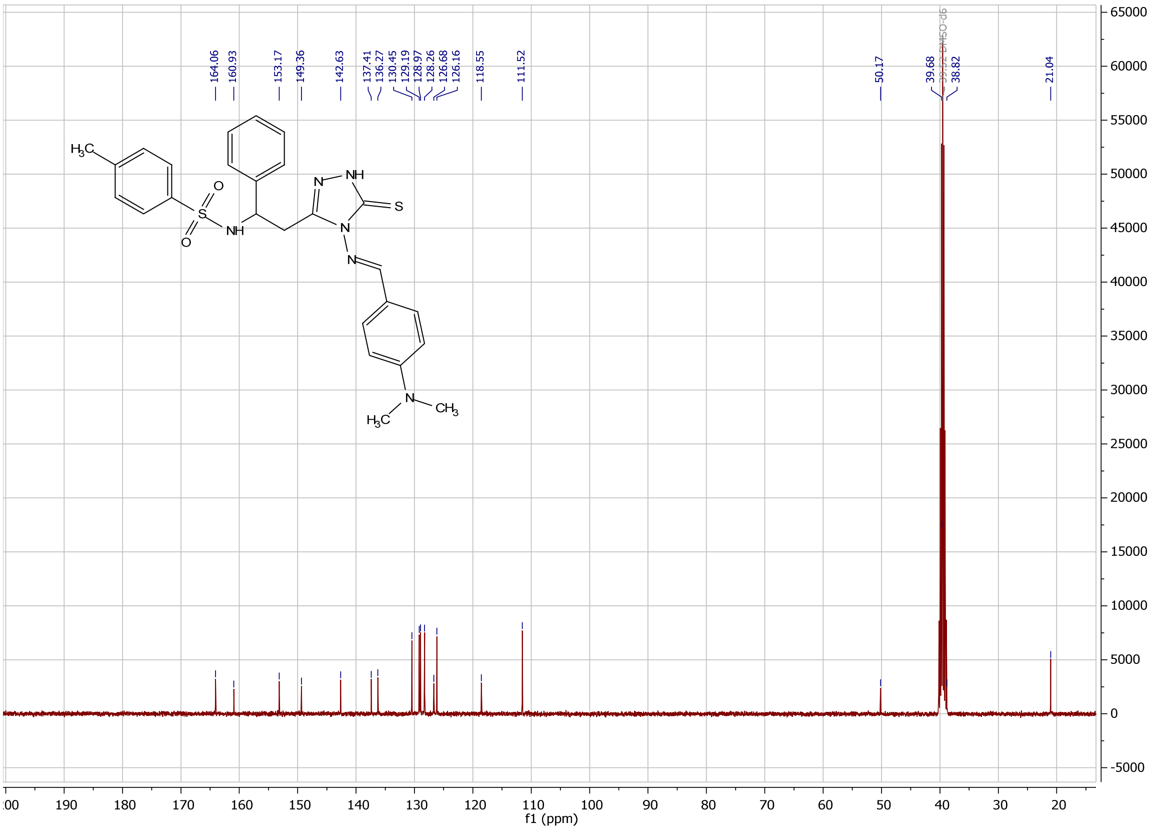

S32. ^{13}C NMR spectra of (*E*)-*N*-{2-[4-[[4-(dimethylamino)benzylidene]amino]-5-thioxo-4,5-dihydro-1*H*-1,2,4-triazol-3-yl]-1-phenylethyl}-4-methylbenzenesulfonamide (**13d**)

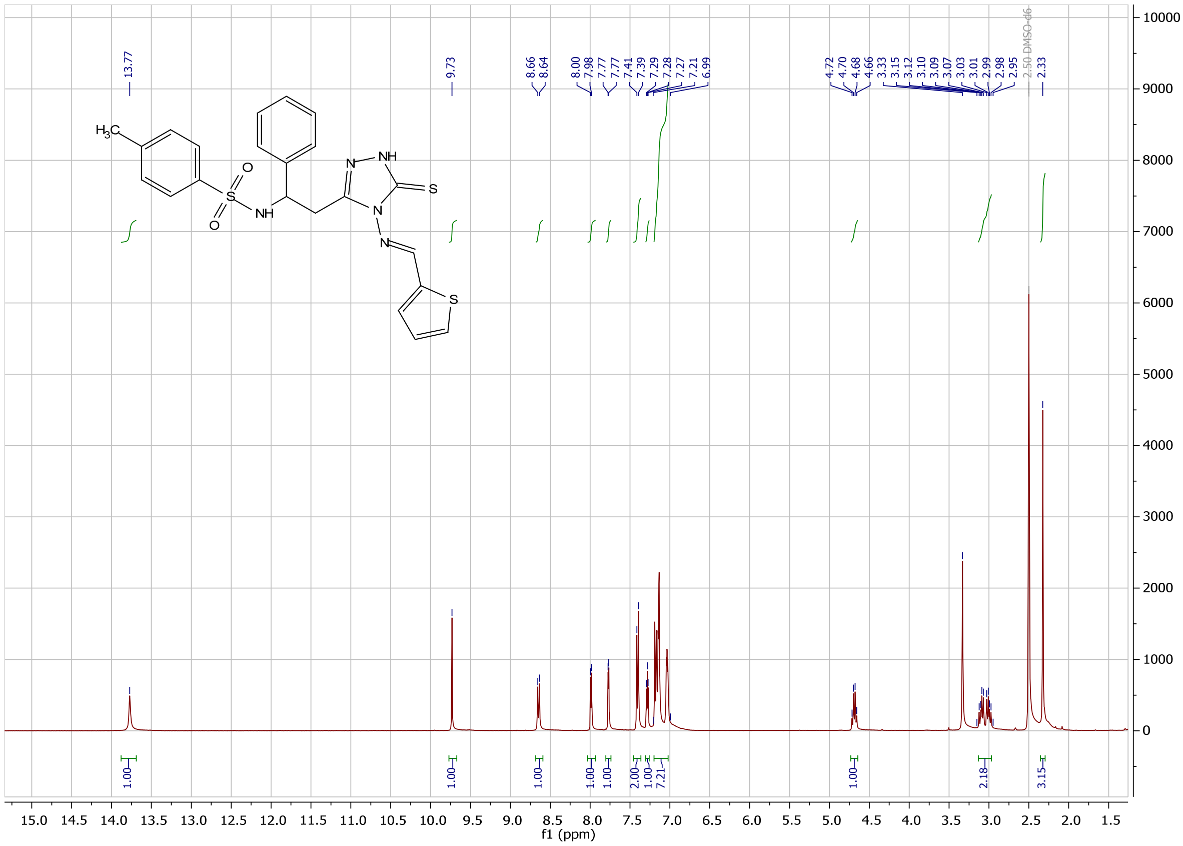

S33. ^{1}H NMR spectra of (*E*)-4-methyl-*N*-{1-phenyl-2-[4-[(thiophen-2-ylmethylene)amino]-5-thioxo-4,5-dihydro-1*H*-1,2,4-triazol-3-yl]ethyl}benzenesulfonamide (**13e**)

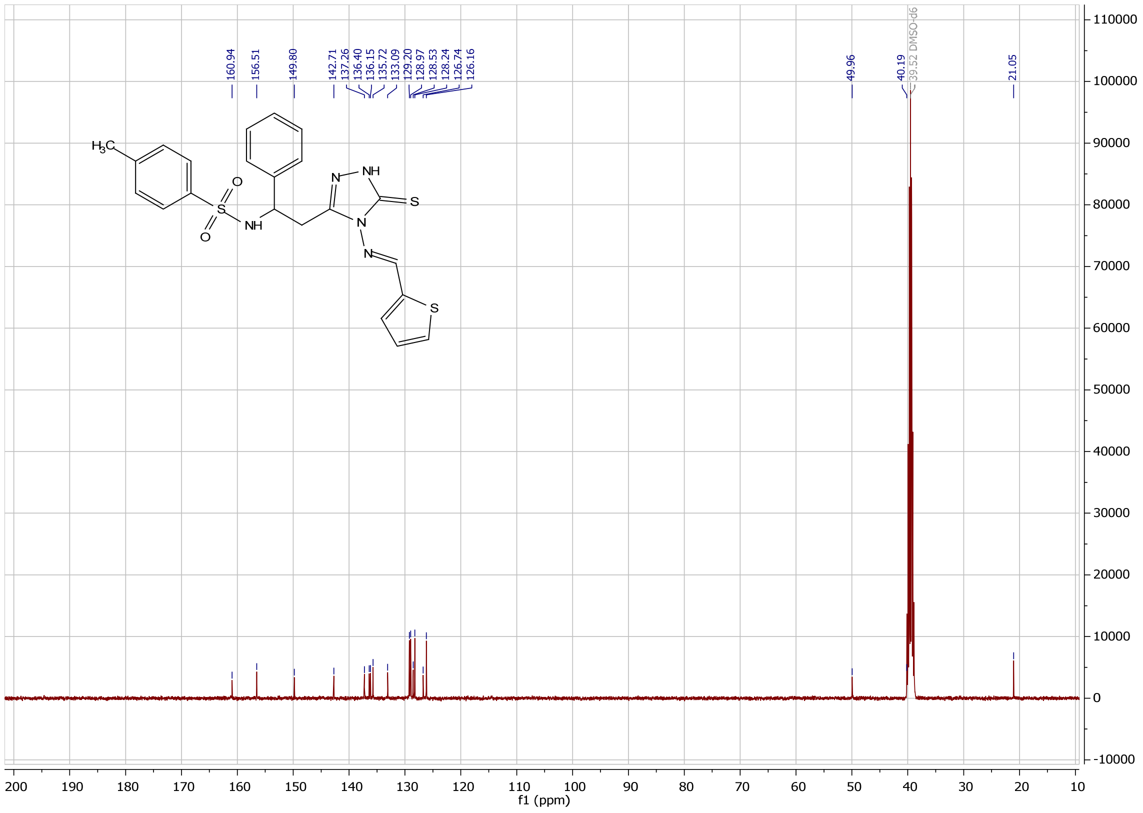

S34. ^{13}C NMR spectra of (*E*)-4-methyl-*N*-{1-phenyl-2-[4-[(thiophen-2-ylmethylene)amino]-5-thioxo-4,5-dihydro-1*H*-1,2,4-triazol-3-yl]ethyl}benzenesulfonamide (**13e**)

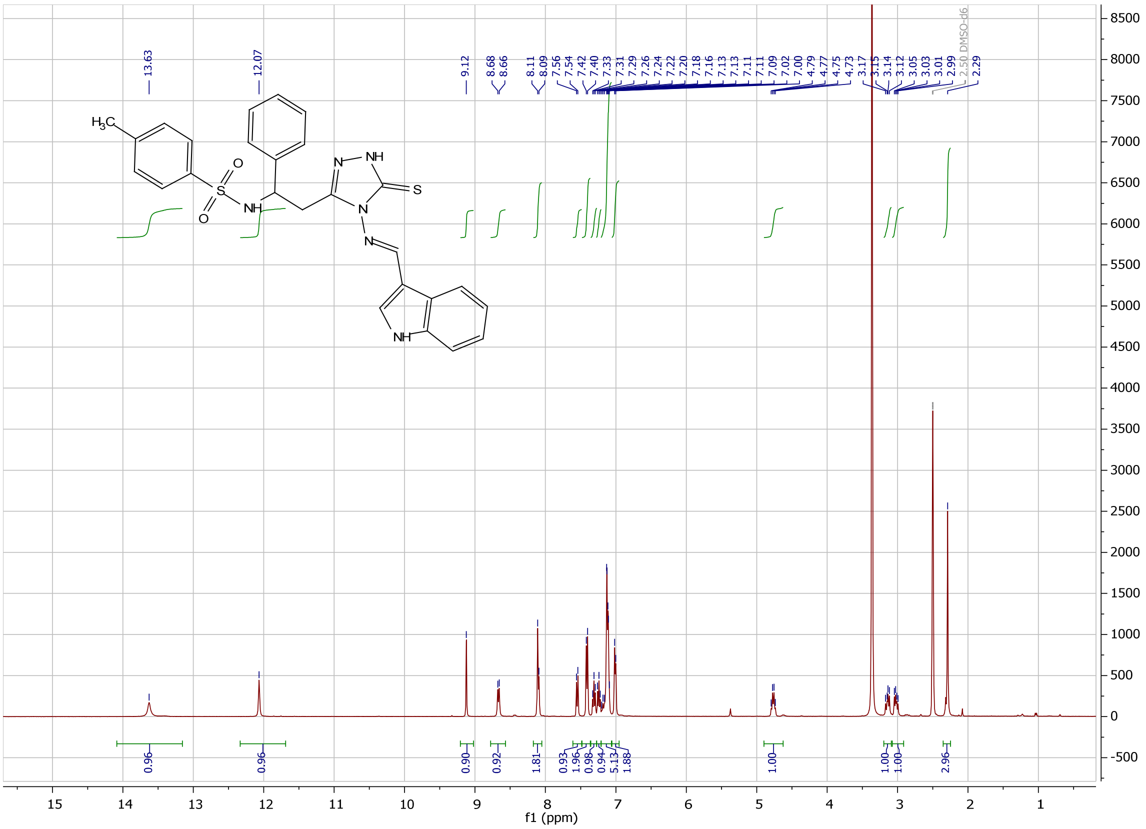

S35. ^{1}H NMR spectra of (*E*)-*N*-[2-{4-[[(1*H*-indol-3-yl)methylene]amino]-5-thioxo-4,5-dihydro-1*H*-1,2,4-triazol-3-yl}-1-phenylethyl]-4-methylbenzenesulfonamide (**13f**)

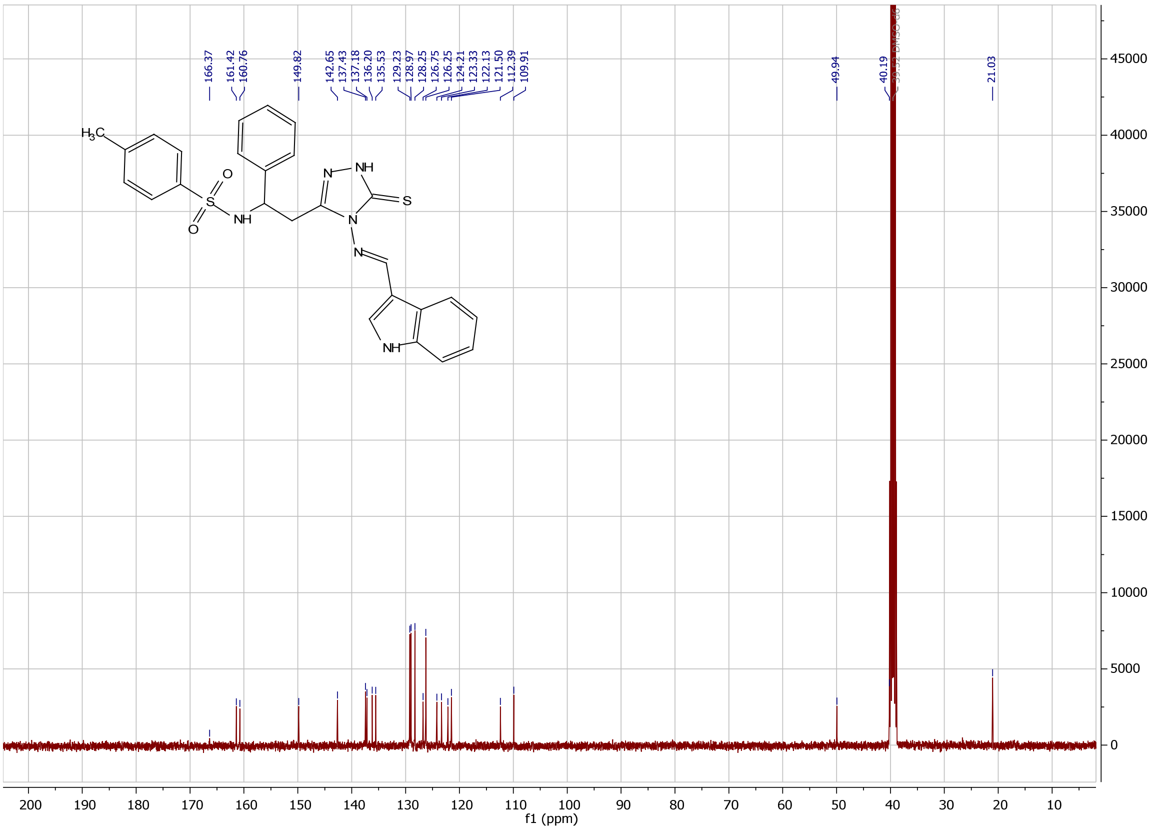

S36. ^{13}C NMR spectra of (*E*)-*N*-[2-{4-[[(1*H*-indol-3-yl)methylene]amino]-5-thioxo-4,5-dihydro-1*H*-1,2,4-triazol-3-yl}-1-phenylethyl]-4-methylbenzenesulfonamide (**13f**)

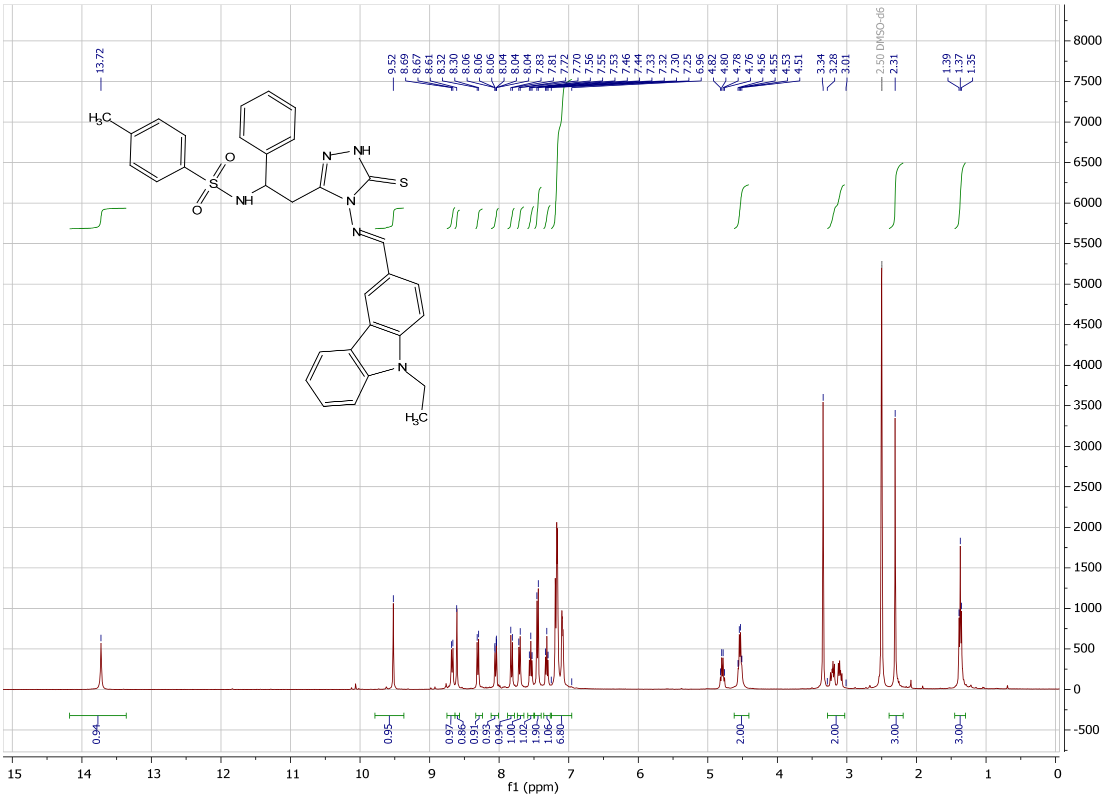

S37. ^{1}H NMR spectra of (*E*)-*N*-[2-{4-[[(9-ethyl-9*H*-carbazol-3-yl)methylene]amino]-5-thioxo-4,5-dihydro-1*H*-1,2,4-triazol-3-yl}-1-phenylethyl]-4-methylbenzenesulfonamide (**13g**)

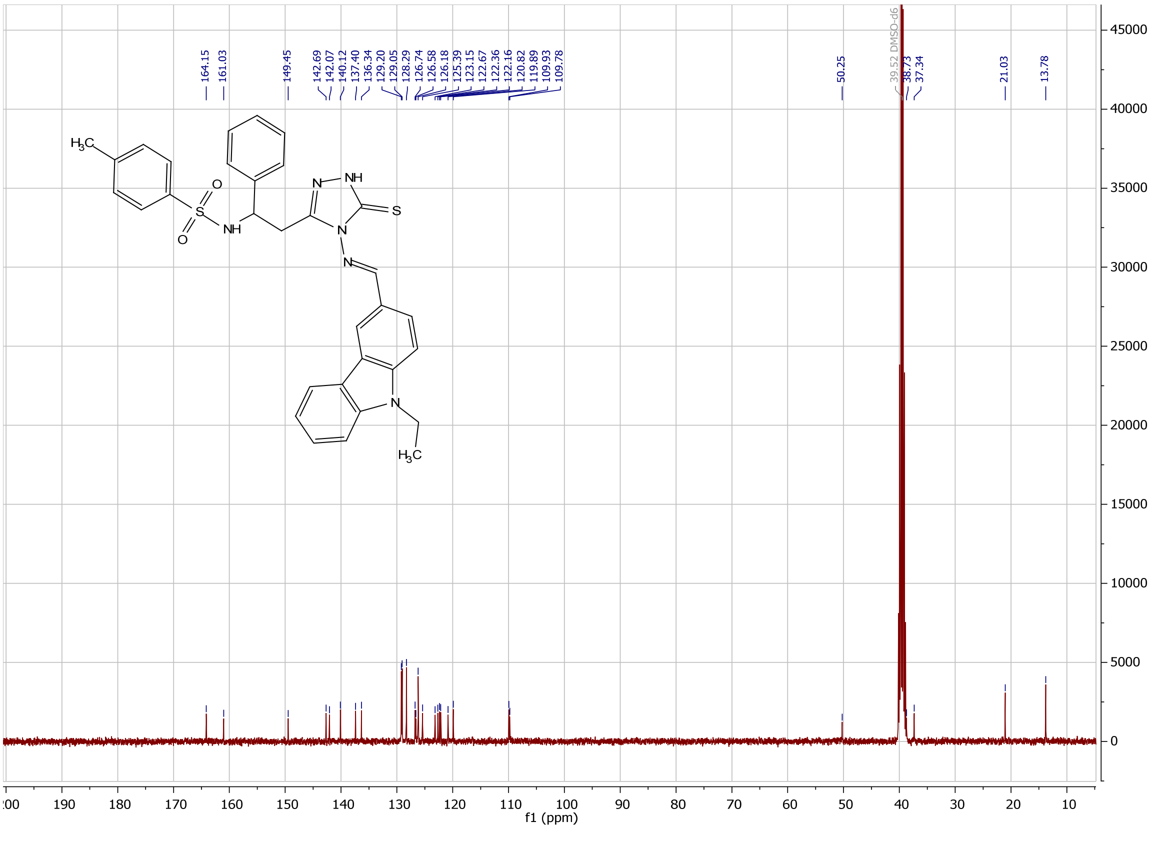

S38. ^{13}C NMR spectra of (*E*)-*N*-[2-{4-[[(9-ethyl-9*H*-carbazol-3-yl)methylene]amino]-5-thioxo-4,5-dihydro-1*H*-1,2,4-triazol-3-yl}-1-phenylethyl]-4-methylbenzenesulfonamide (**13g**)

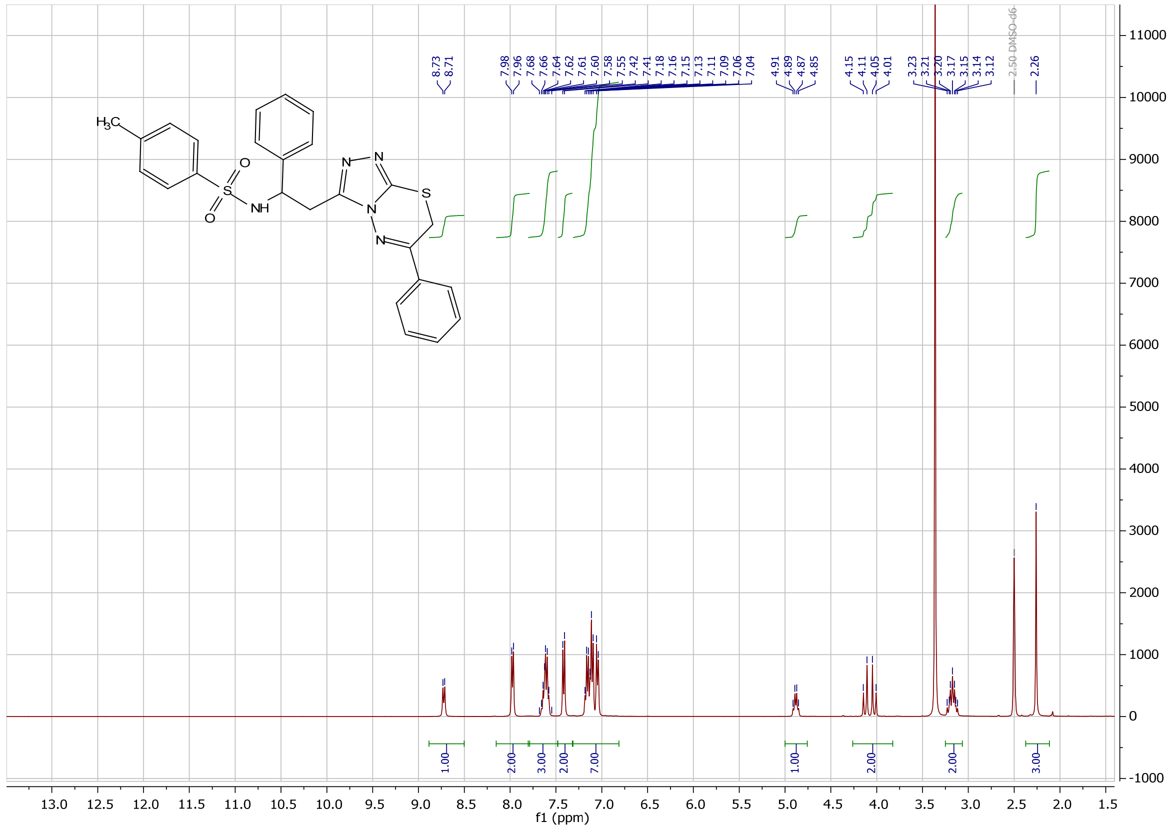

S39. ^{1}H NMR spectra of 4-methyl-*N*-[1-phenyl-2-(6-phenyl-7*H*-[1,2,4]triazolo[3,4-*b*][1,3,4]thiadiazin-3-yl)ethyl]benzenesulfonamide (**14a**)

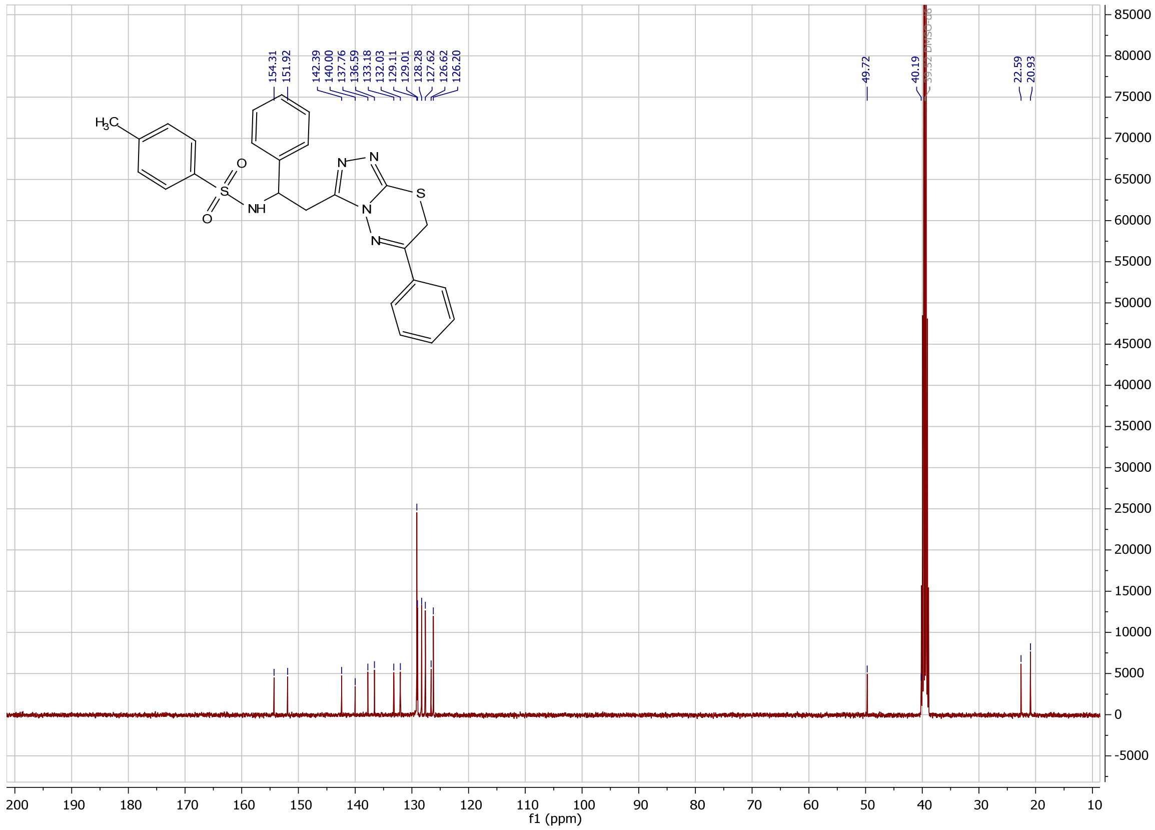

S40. ^{13}C NMR spectra of 4-methyl-*N*-[1-phenyl-2-(6-phenyl-7*H*-[1,2,4]triazolo[3,4-*b*][1,3,4]thiadiazin-3-yl)ethyl]benzenesulfonamide (**14a**)

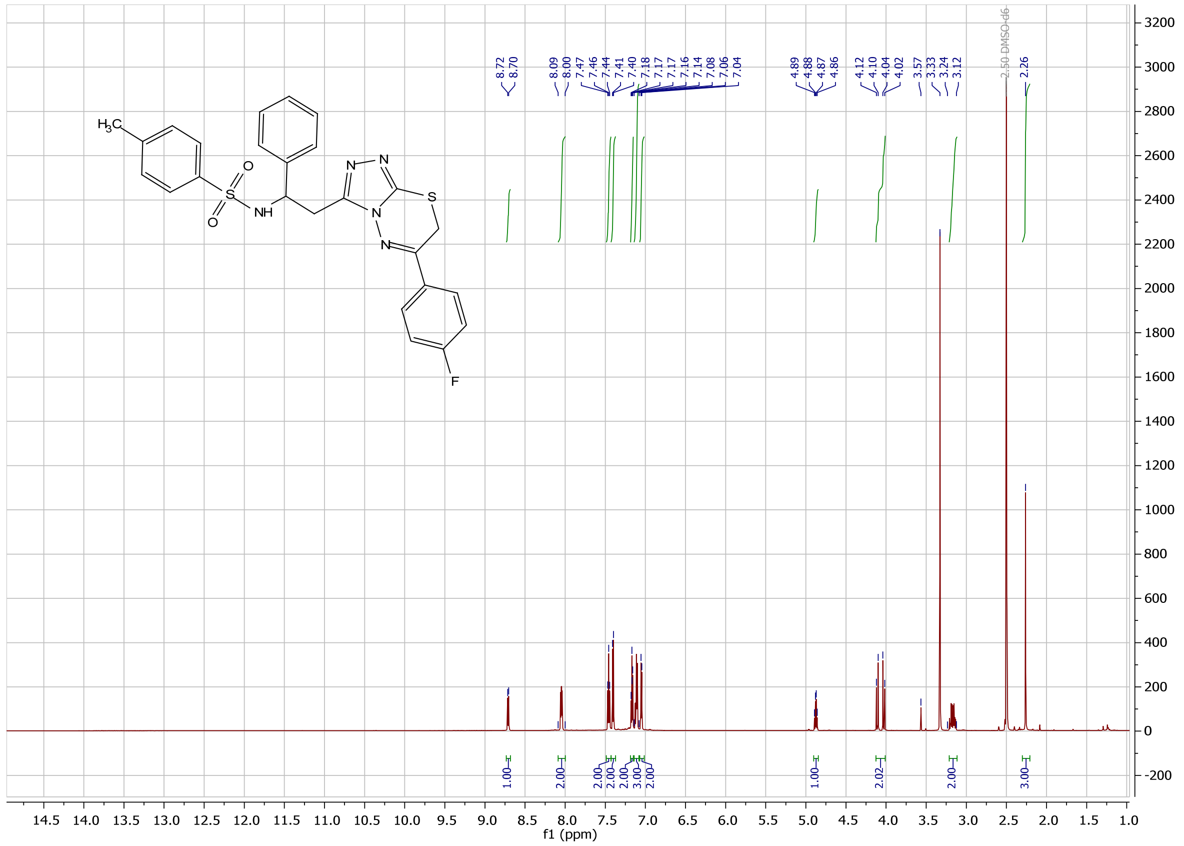

S41. ^{1}H NMR spectra of *N*-{2-[6-(4-fluorophenyl)-7*H*-[1,2,4]triazolo[3,4-*b*][1,3,4]thiadiazin-3-yl]-1-phenylethyl}-4-methylbenzenesulfonamide (**14b**)

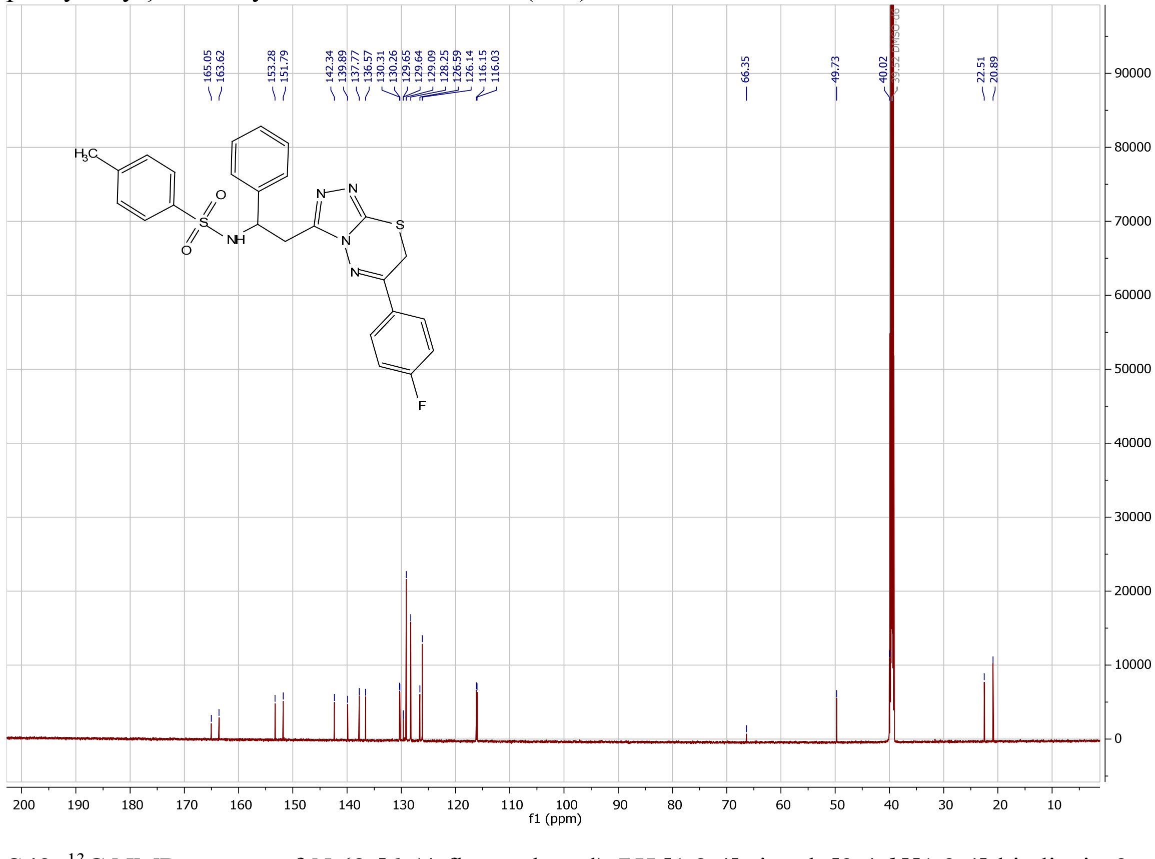

S42. ^{13}C NMR spectra of *N*-{2-[6-(4-fluorophenyl)-7*H*-[1,2,4]triazolo[3,4-*b*][1,3,4]thiadiazin-3-yl]-1-phenylethyl}-4-methylbenzenesulfonamide (**14b**)

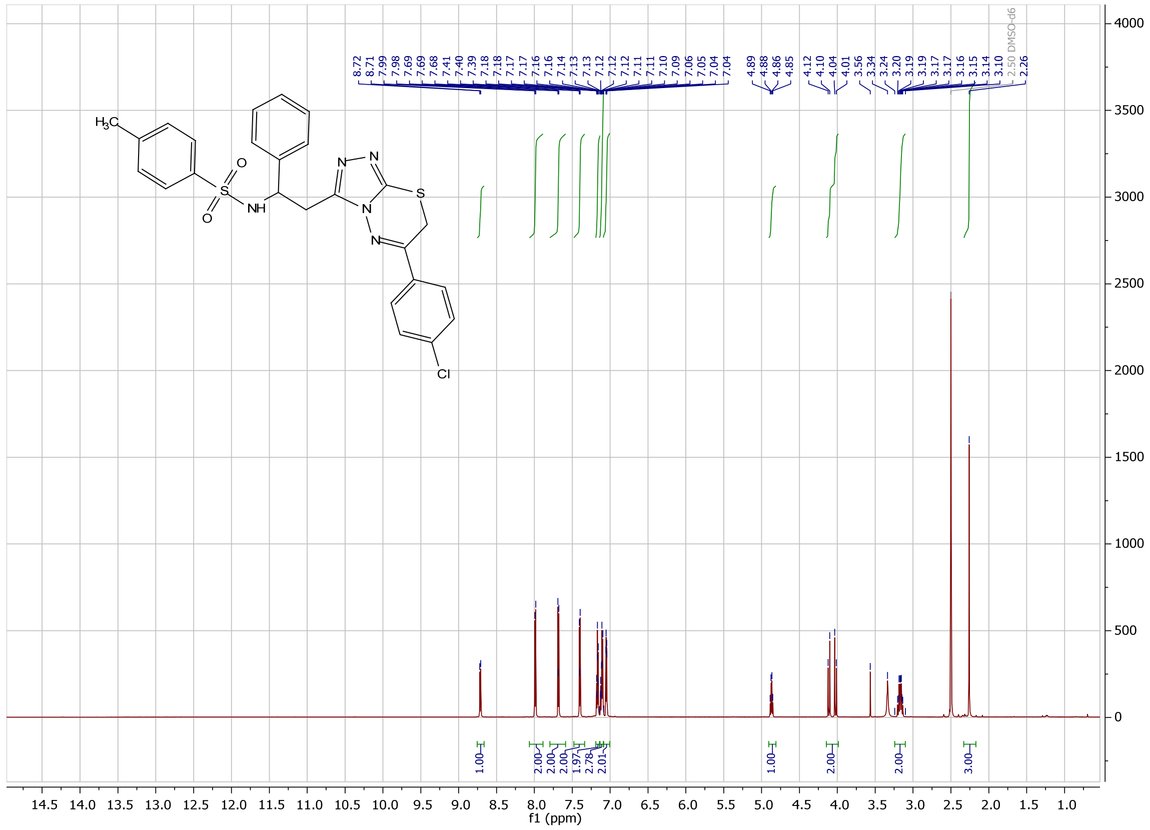

S43. ^{1}H NMR spectra of *N*-{2-[6-(4-chlorophenyl)-7*H*-[1,2,4]triazolo[3,4-*b*][1,3,4]thiadiazin-3-yl]-1-phenylethyl}-4-methylbenzenesulfonamide (**14c**)

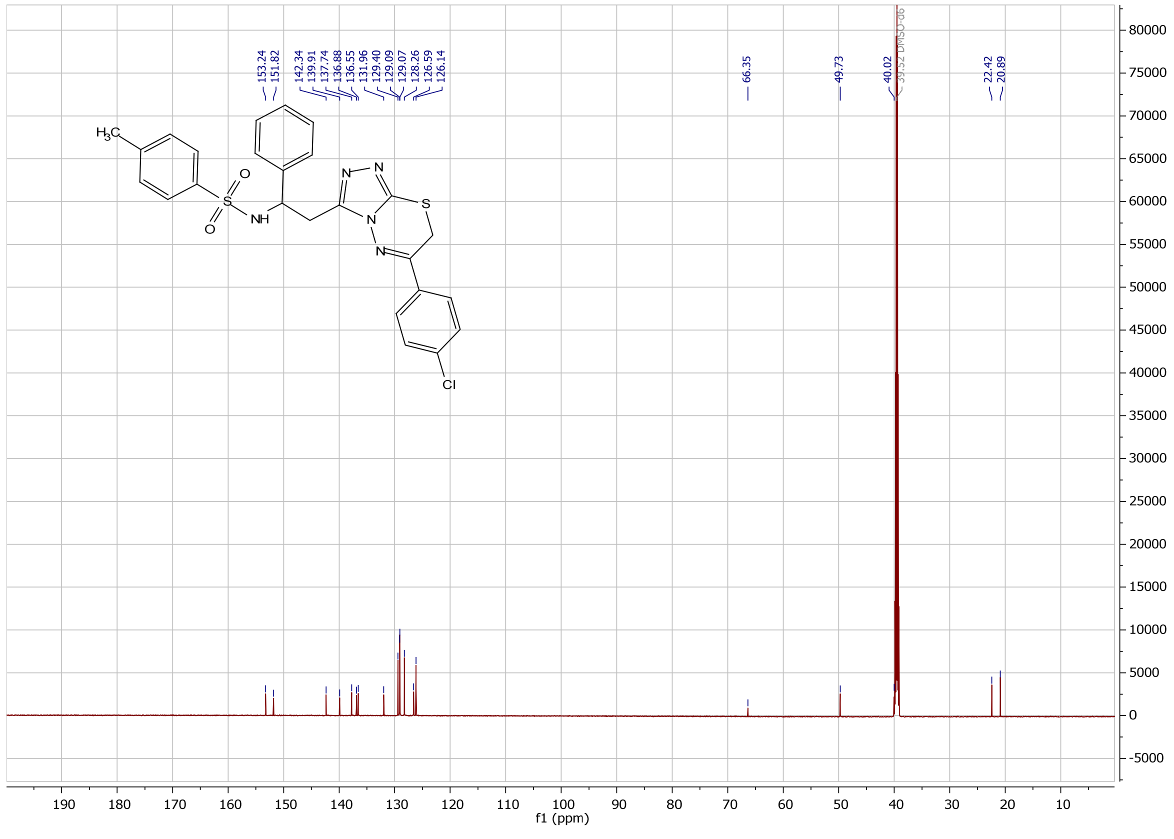

S44. ^{13}C NMR spectra of *N*-{2-[6-(4-chlorophenyl)-7*H*-[1,2,4]triazolo[3,4-*b*][1,3,4]thiadiazin-3-yl]-1-phenylethyl}-4-methylbenzenesulfonamide (**14c**)

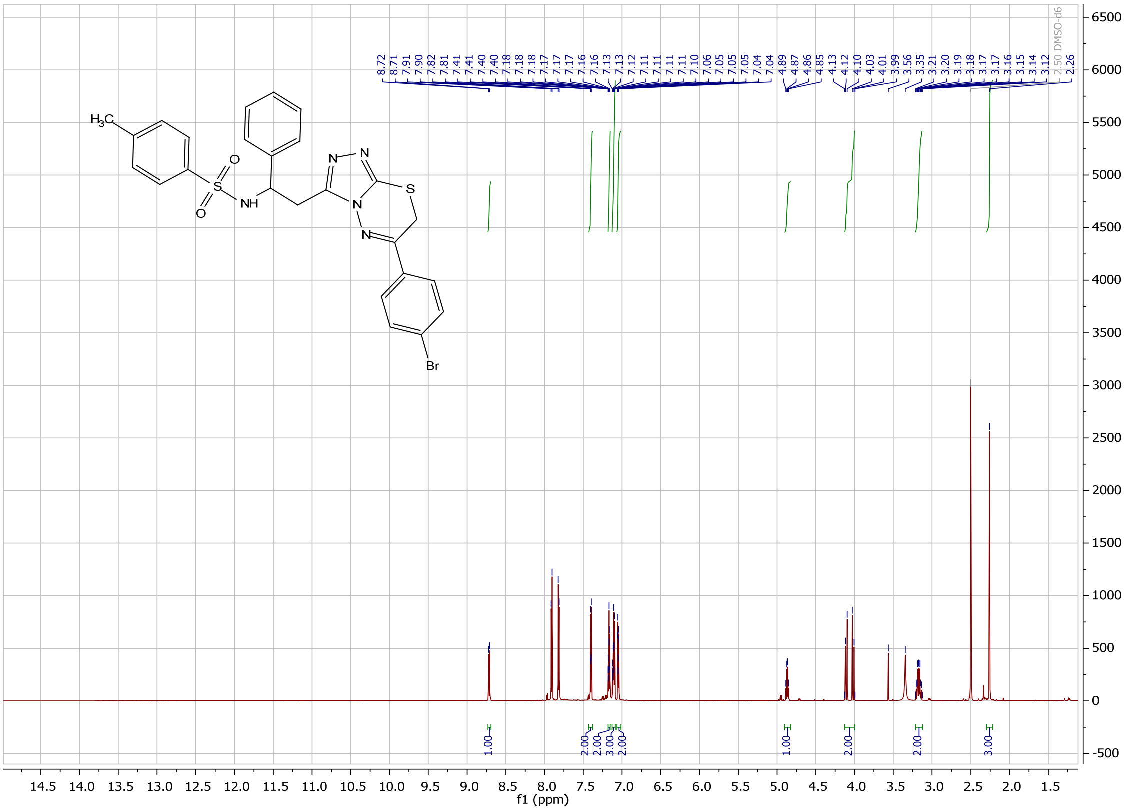

S45. ^{1}H NMR spectra of *N*-{2-[6-(4-bromophenyl)-7*H*-[1,2,4]triazolo[3,4-*b*][1,3,4]thiadiazin-3-yl]-1-phenylethyl}-4-methylbenzenesulfonamide (**14d**)

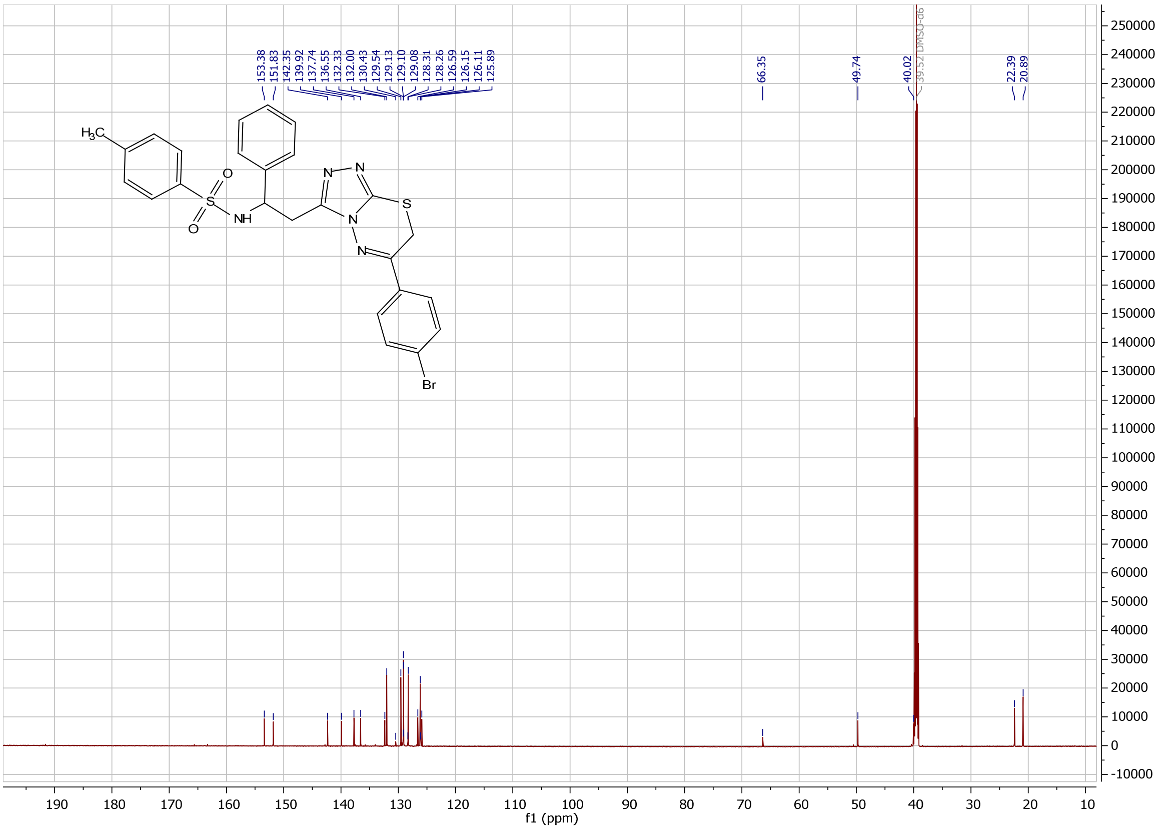

S46. ^{13}C NMR spectra of *N*-{2-[6-(4-bromophenyl)-7*H*-[1,2,4]triazolo[3,4-*b*][1,3,4]thiadiazin-3-yl]-1-phenylethyl}-4-methylbenzenesulfonamide (**14d**)

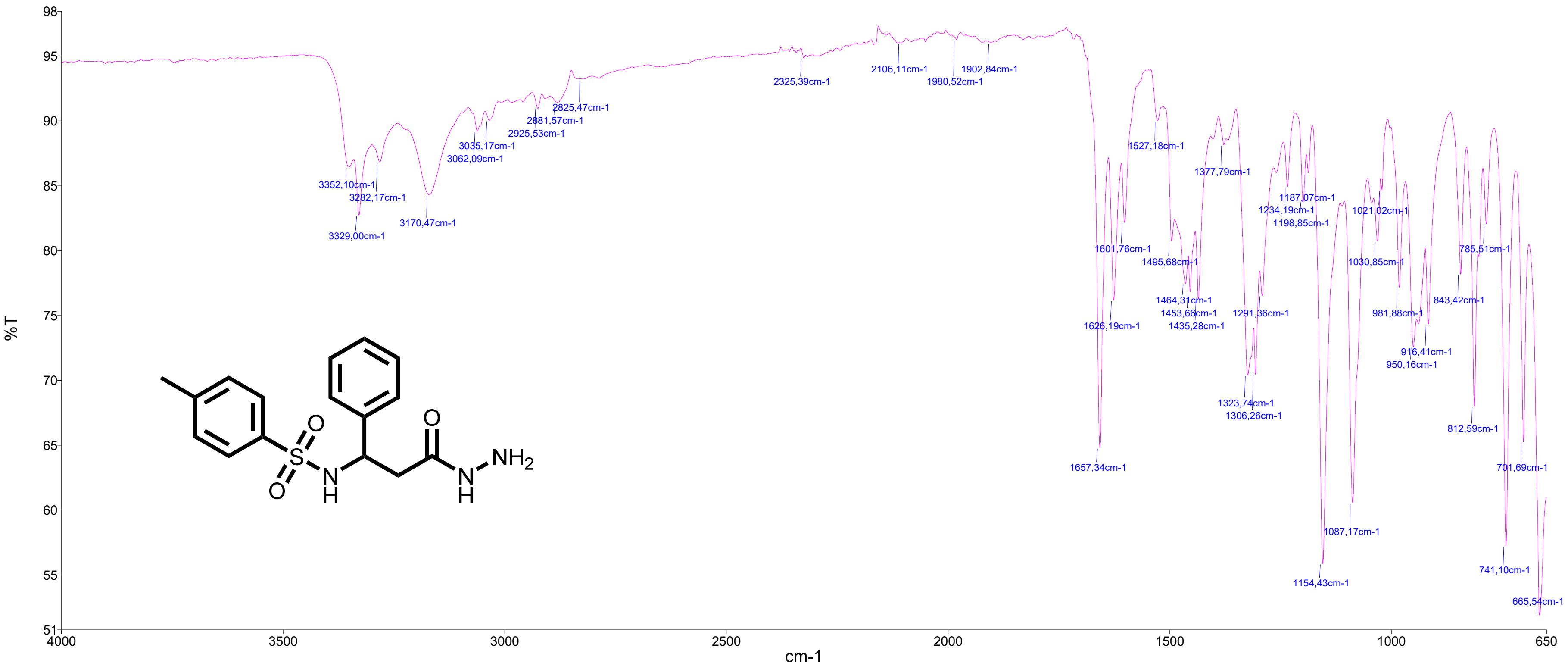

S47. FTIR spectra of *N*-[3-hydrazinyl-3-oxo-1-phenylpropyl]-4-methylbenzene-1-sulfonamide (**4**)

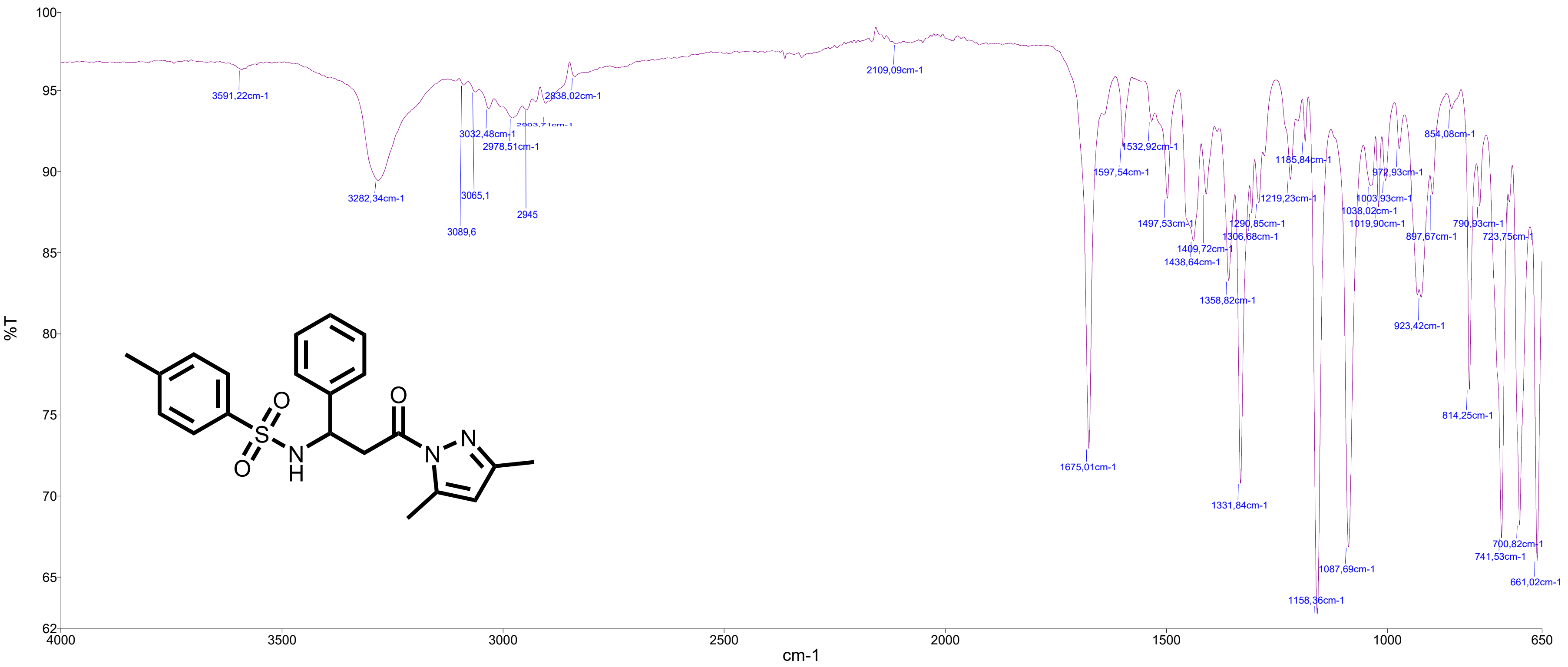

S48. FTIR spectra of *N*-[3-(3,5-dimethyl-1*H*-pyrazol-1-yl)-3-oxo-1-phenylpropyl]-4-methylbenzenesulfonamide (**5**)

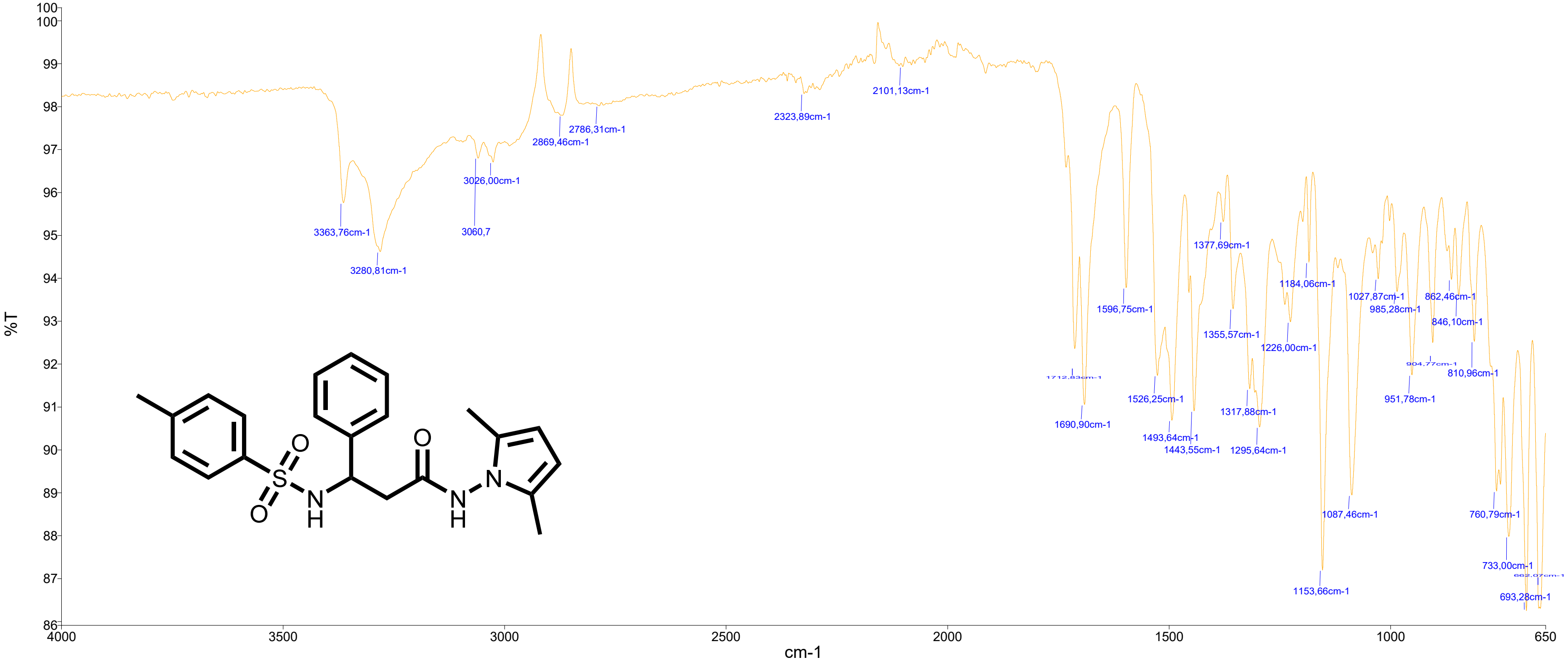

S49. FTIR spectra of *N*-(2,5-dimethyl-1*H*-pyrrol-1-yl)-3-[(4-methylphenyl)sulfonamido]-3-phenylpropanamide (**6**)

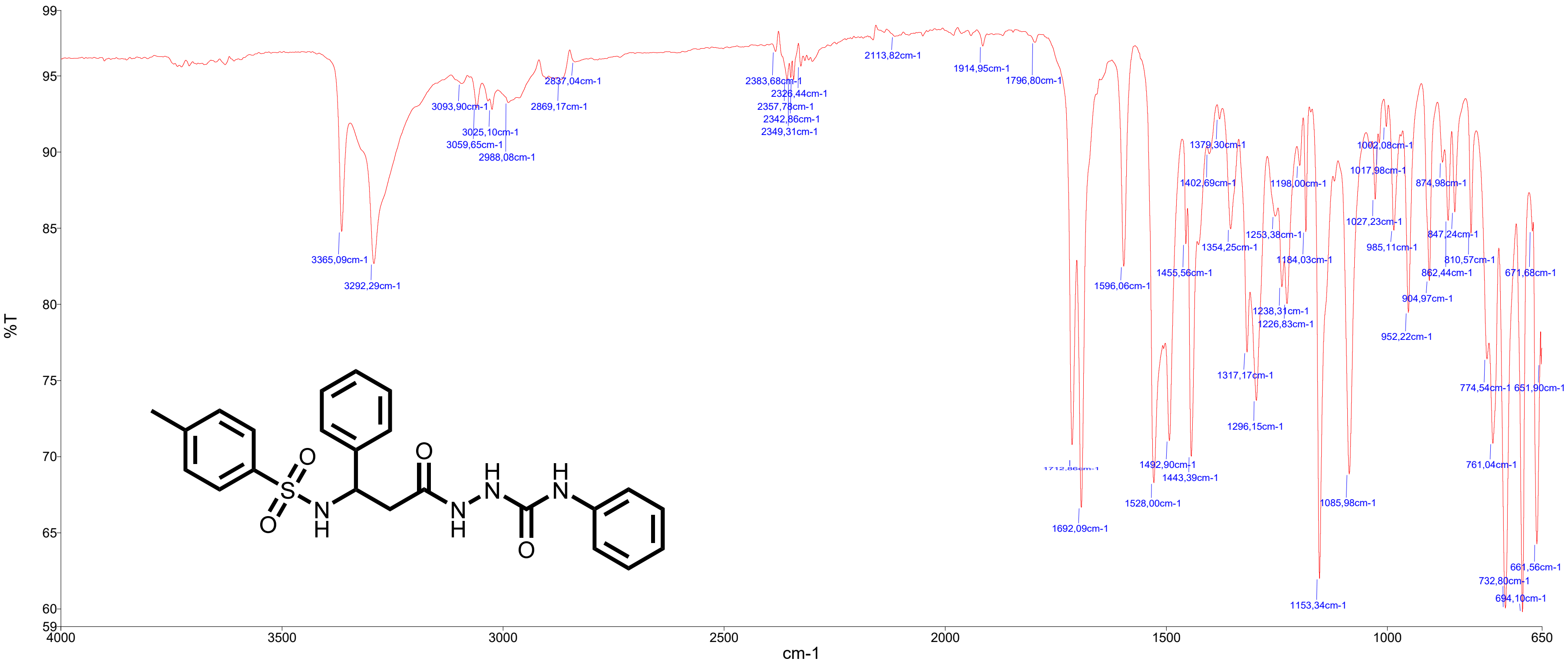

S50. FTIR spectra of 2-[3-[(4-methylphenyl)sulfonamido]-3-phenylpropanoyl]-*N*-phenylhydrazine-1-carboxamide (**7a**)

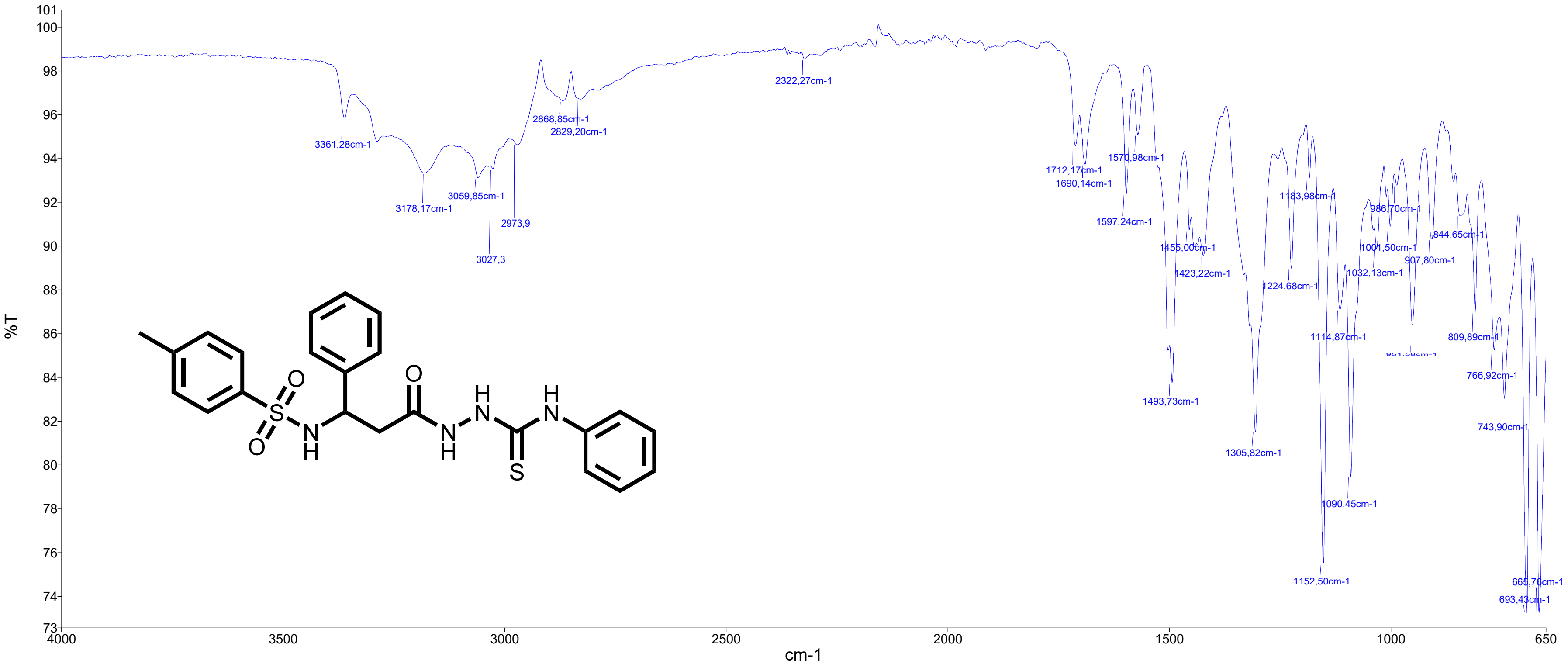

S51. FTIR spectra of 2-[3-[(4-methylphenyl)sulfonamido]-3-phenylpropanoyl]-*N*-phenylhydrazine-1-carbothioamide (**7b**)

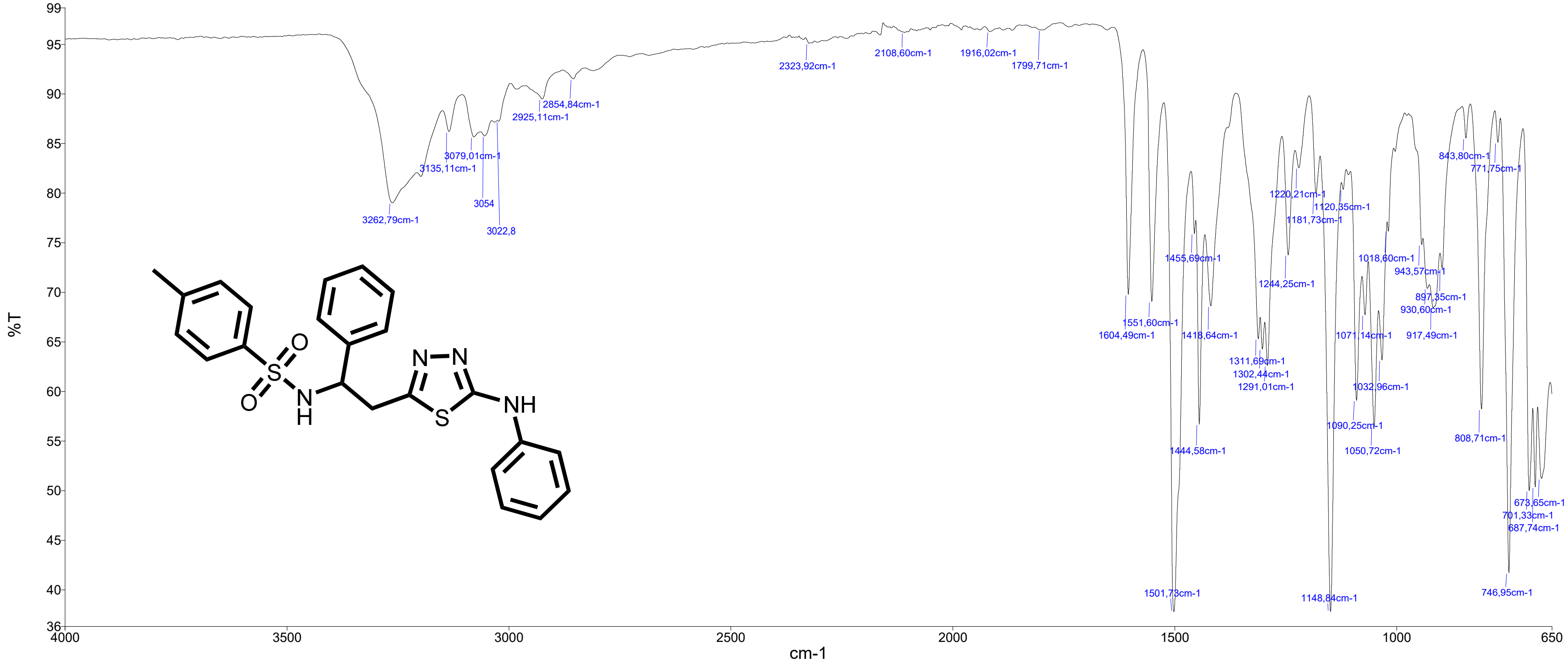

S52. FTIR spectra of 4-methyl-*N*-[1-phenyl-2-[5-(phenylamino)-1,3,4-thiadiazol-2-yl]ethyl]benzenesulfonamide (**8**)

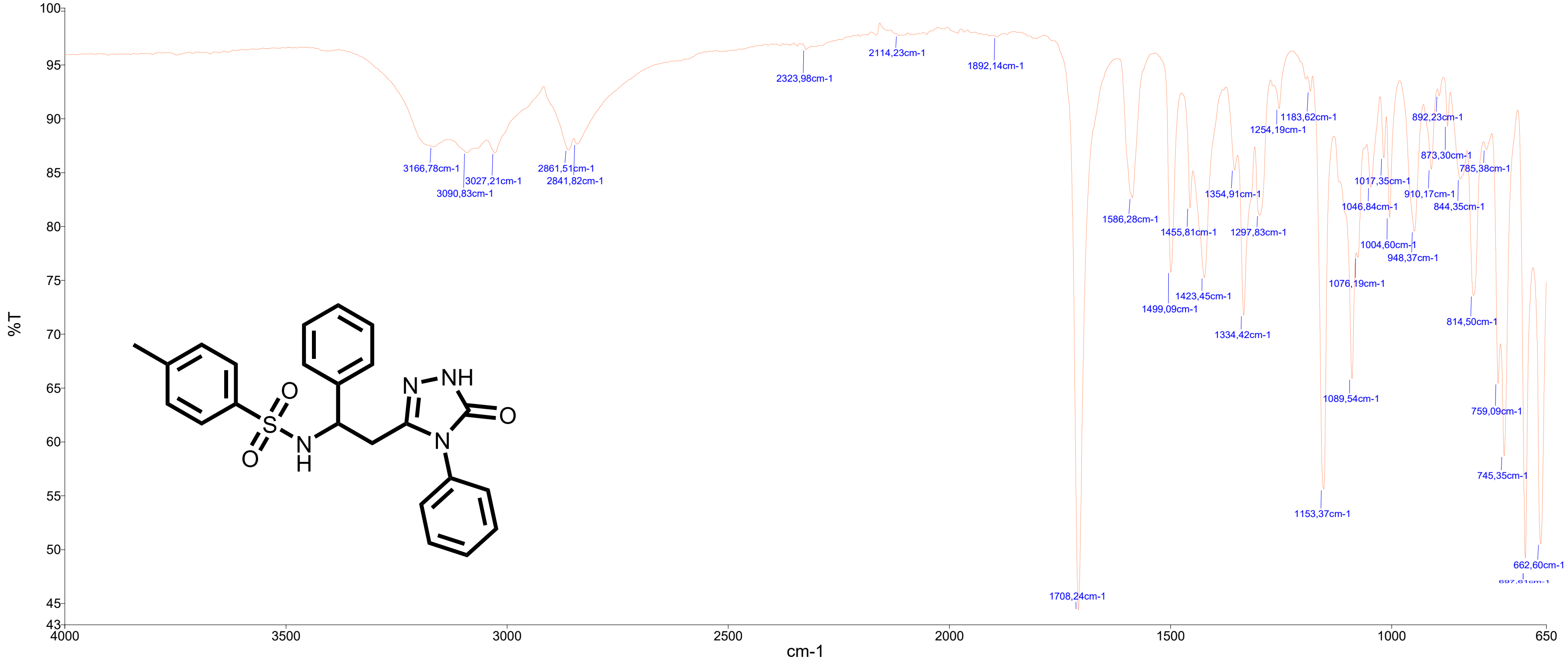

S53. ^{1}H NMR spectra of 4-methyl-*N*-[2-(5-oxo-4-phenyl-4,5-dihydro-1*H*-1,2,4-triazol-3-yl)-1-phenylethyl]benzenesulfonamide (**9a**)

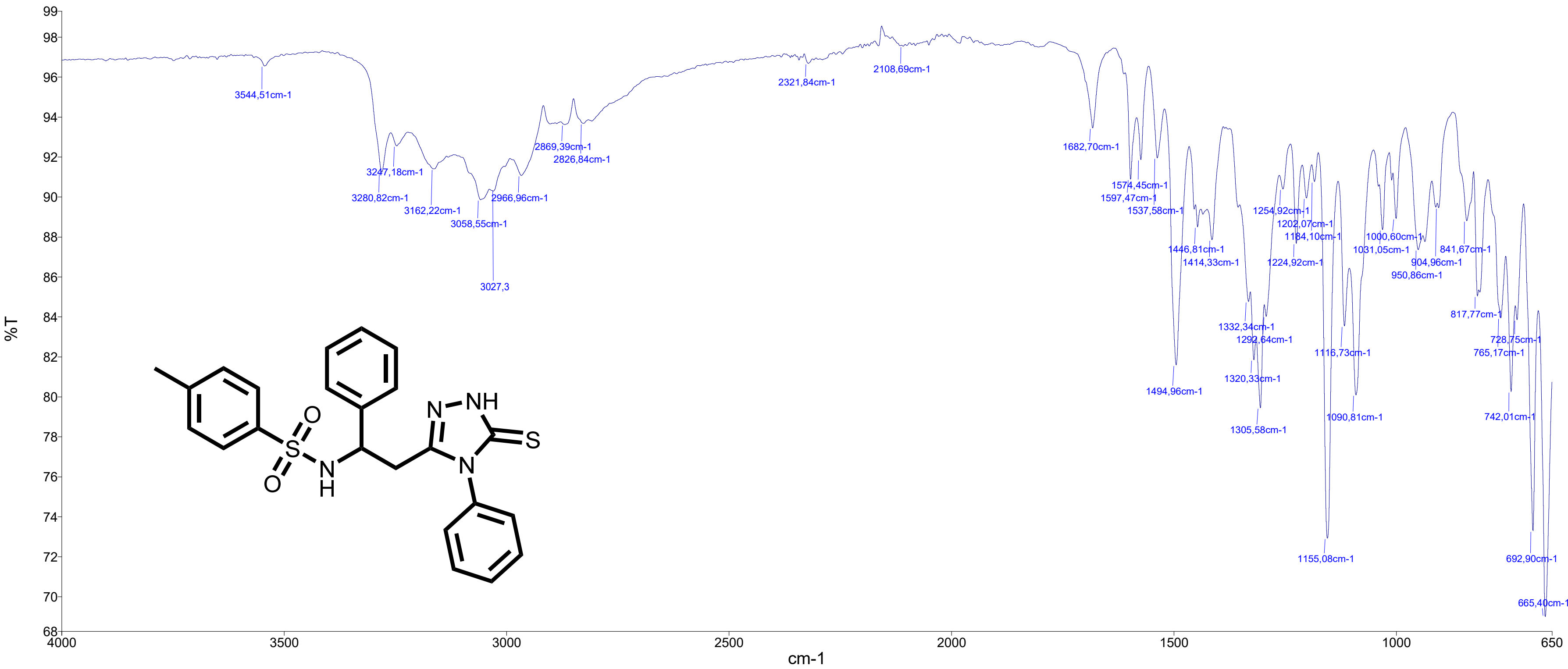

S54. FTIR spectra of **S20.** ^{13}C NMR spectra of 4-methyl-*N*-[2-(5-thioxo-4-phenyl-4,5-dihydro-1*H*-1,2,4-triazol-3-yl)-1-phenylethyl]benzenesulfonamide (**9b**)

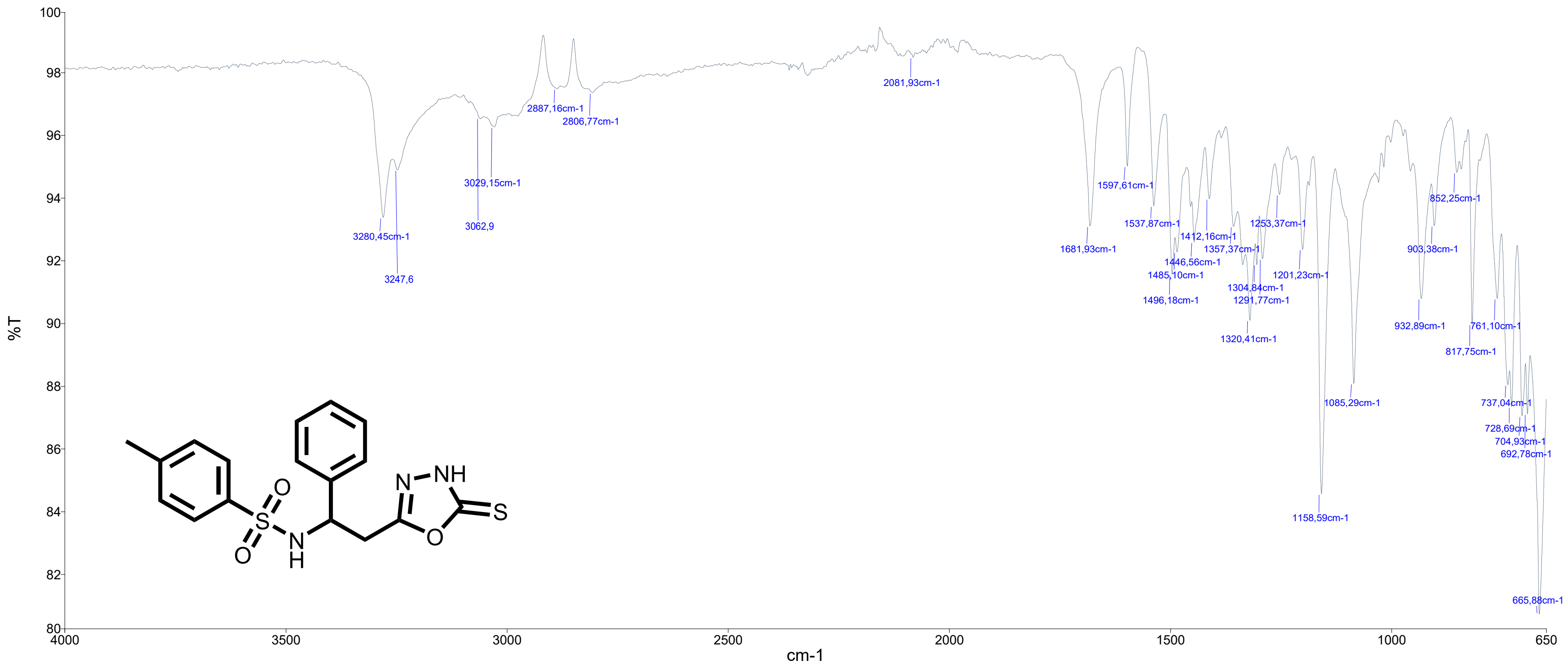

S55. FTIR spectra of 4-methyl-*N*-[1-phenyl-2-(5-thioxo-4,5-dihydro-1,3,4-oxadiazol-2-yl)ethyl]benzenesulfonamide (**11**)

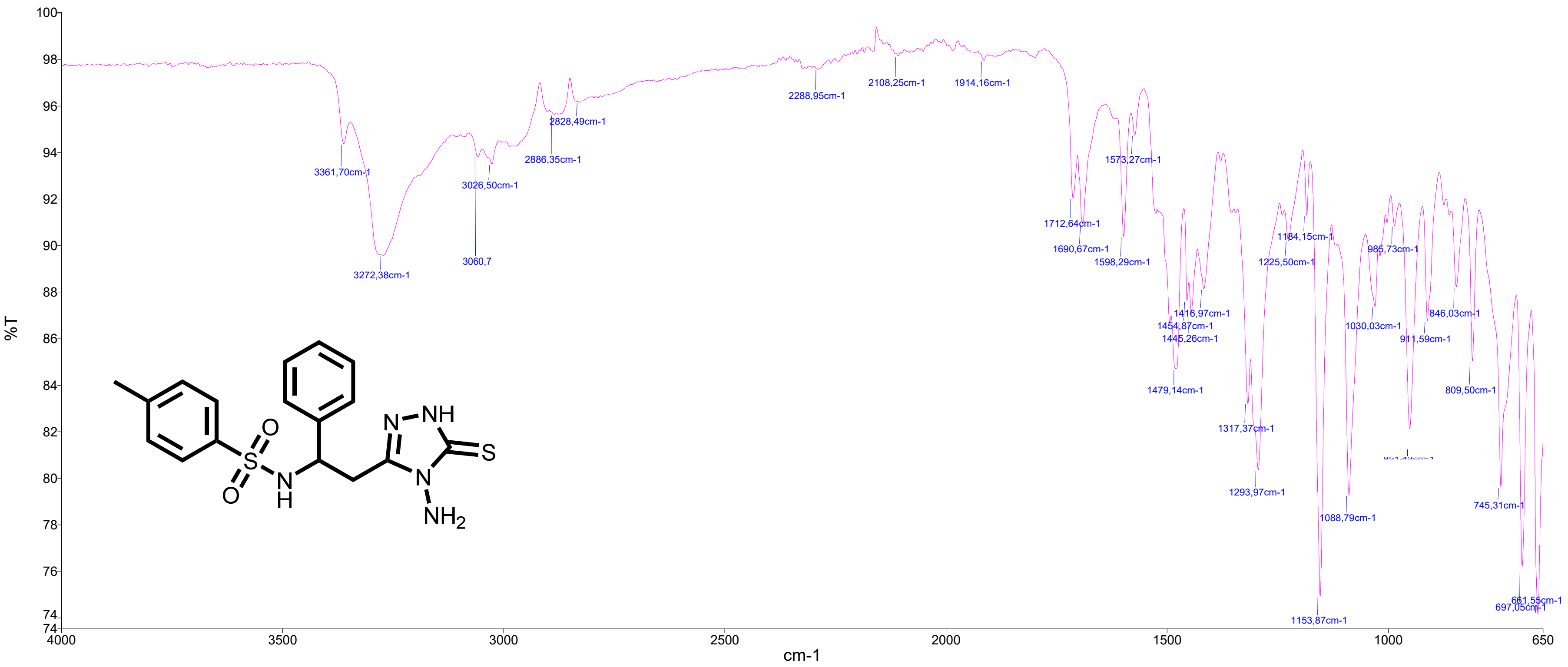

S56. FTIR spectra of *N*-[2-(4-amino-5-thioxo-4,5-dihydro-1*H*-1,2,4-triazol-3-yl)-1-phenylethyl]-4-methylbenzenesulfonamide (**12**)

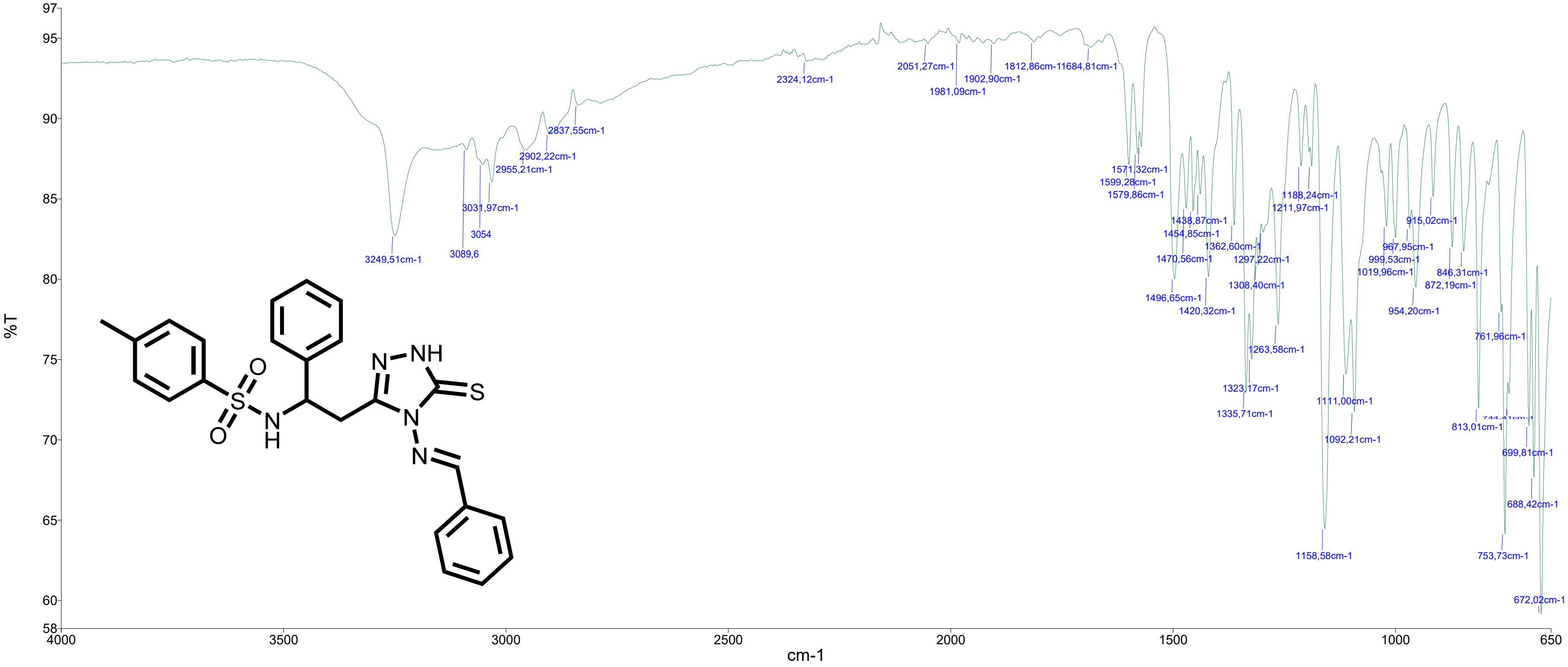

S57. FTIR spectra of (*E*)-*N*-[2-[4-(benzylideneamino)-5-thioxo-4,5-dihydro-1*H*-1,2,4-triazol-3-yl]-1-phenylethyl]-4-methylbenzenesulfonamide (**13a**)

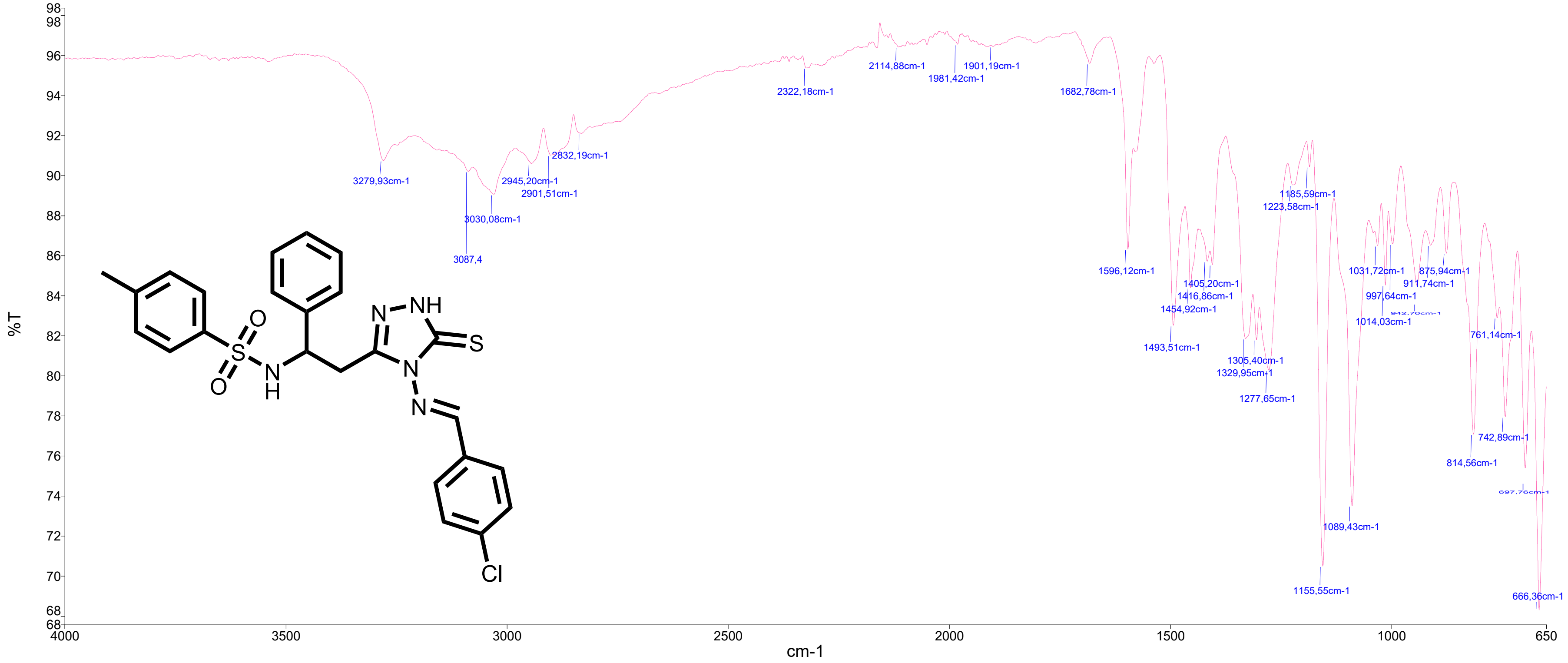

S58. FTIR spectra of (*E*)-*N*-{2-[4-[(4-chlorobenzylidene)amino]-5-thioxo-4,5-dihydro-1*H*-1,2,4-triazol-3-yl]-1-phenylethyl}-4-methylbenzenesulfonamide (**13b**)

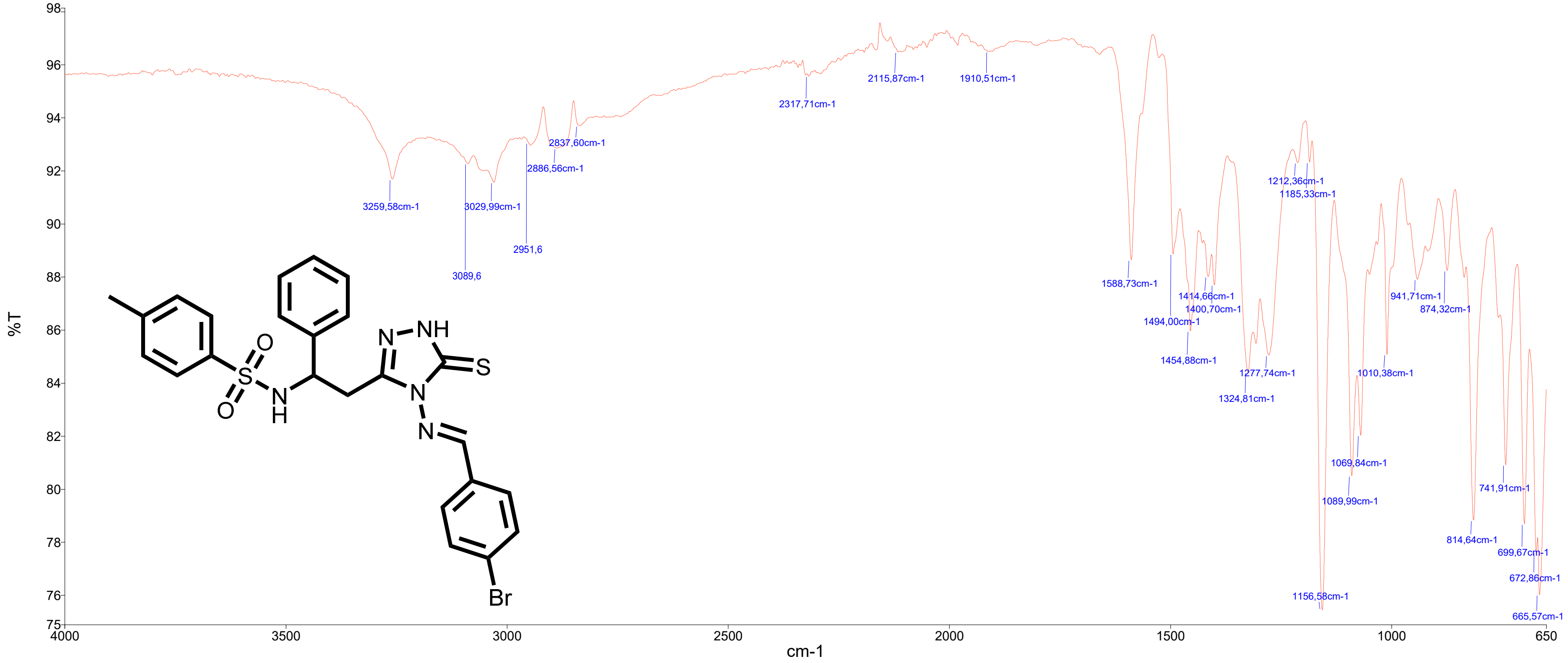

S59. ^{1}H NMR spectra of (*E*)-*N*-{2-[4-[(4-bromobenzylidene)amino]-5-thioxo-4,5-dihydro-1*H*-1,2,4-triazol-3-yl]-1-phenylethyl}-4-methylbenzenesulfonamide (**13c**)

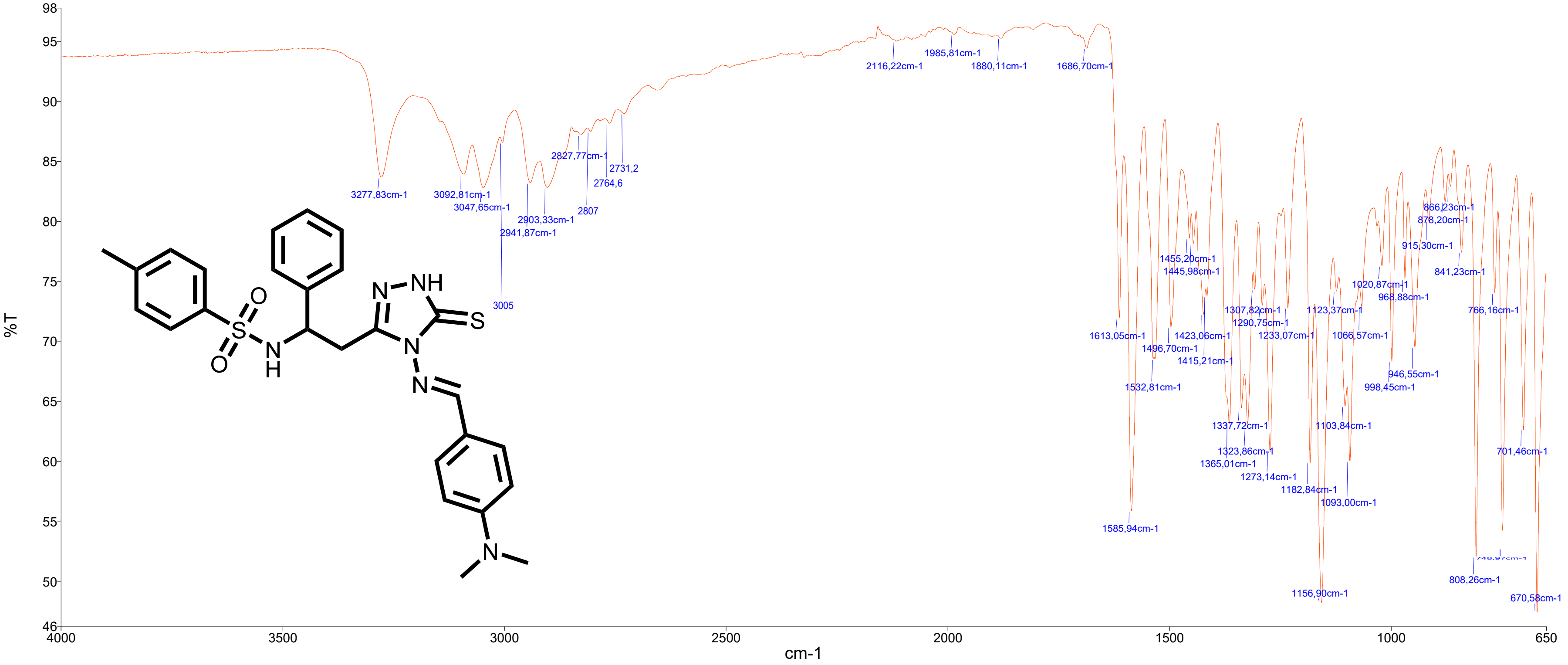

S60. FTIR spectra of (*E*)-*N*-{2-[4-[[4-(dimethylamino)benzylidene]amino]-5-thioxo-4,5-dihydro-1*H*-1,2,4-triazol-3-yl]-1-phenylethyl}-4-methylbenzenesulfonamide (**13d**)

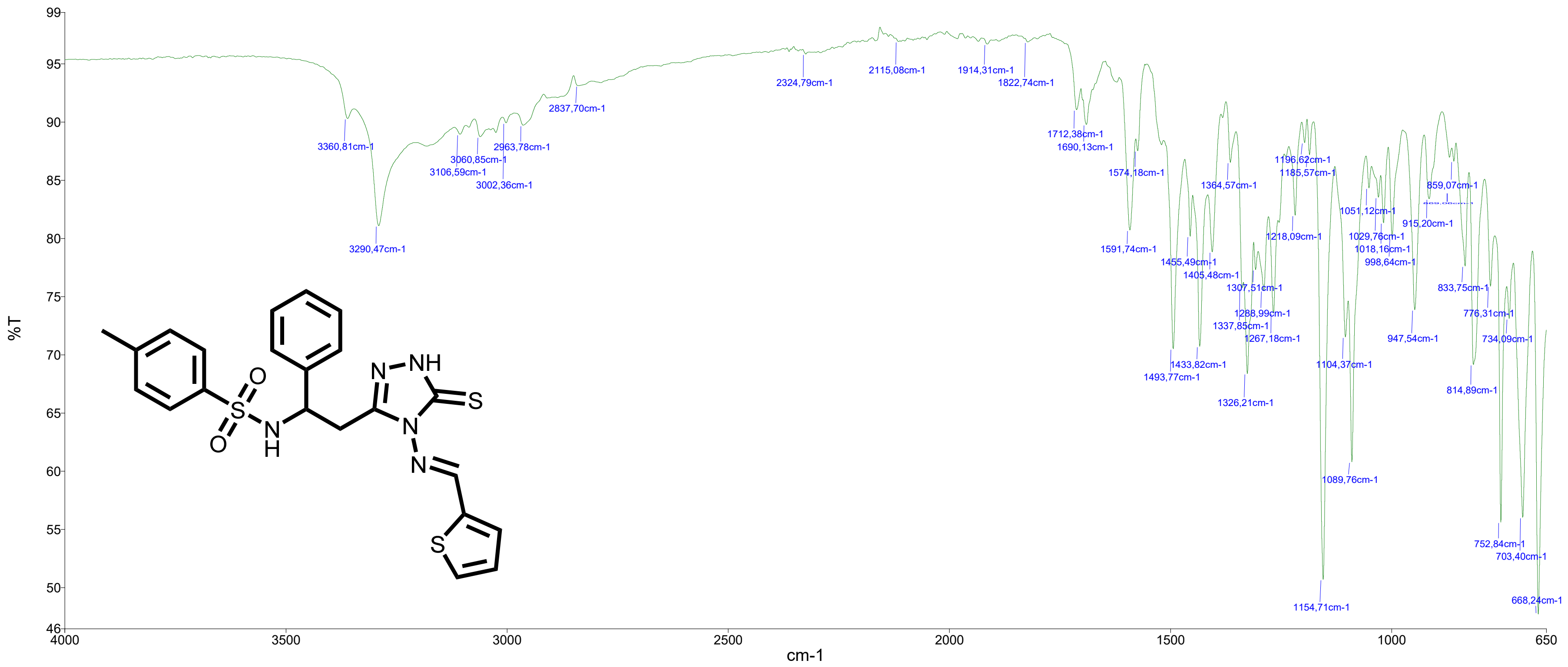

S61. FTIR spectra of (*E*)-4-methyl-*N*-{1-phenyl-2-[4-[(thiophen-2-ylmethylene)amino]-5-thioxo-4,5-dihydro-1*H*-1,2,4-triazol-3-yl]ethyl}benzenesulfonamide (**13e**)

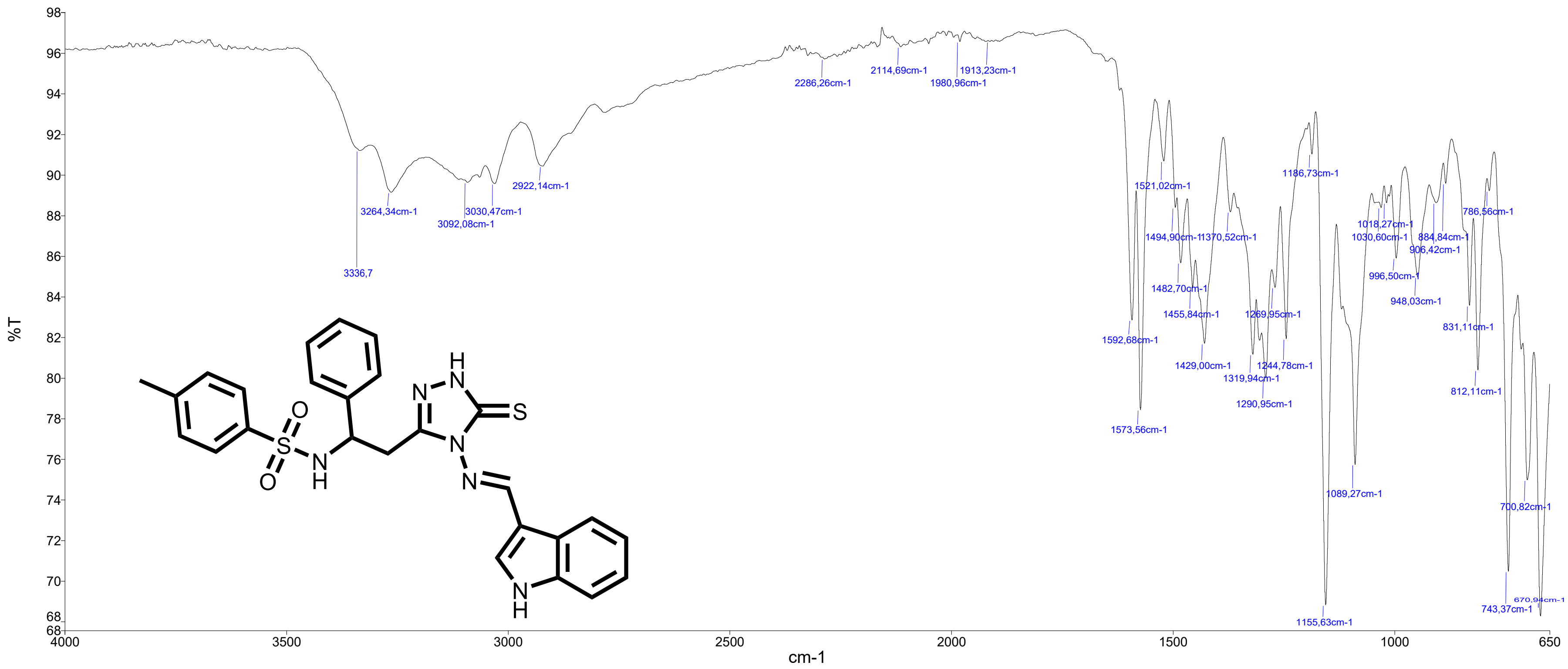

S62. FTIR spectra of (*E*)-*N*-[2-{4-[[(1*H*-indol-3-yl)methylene]amino]-5-thioxo-4,5-dihydro-1*H*-1,2,4-triazol-3-yl}-1-phenylethyl]-4-methylbenzenesulfonamide (**13f**)

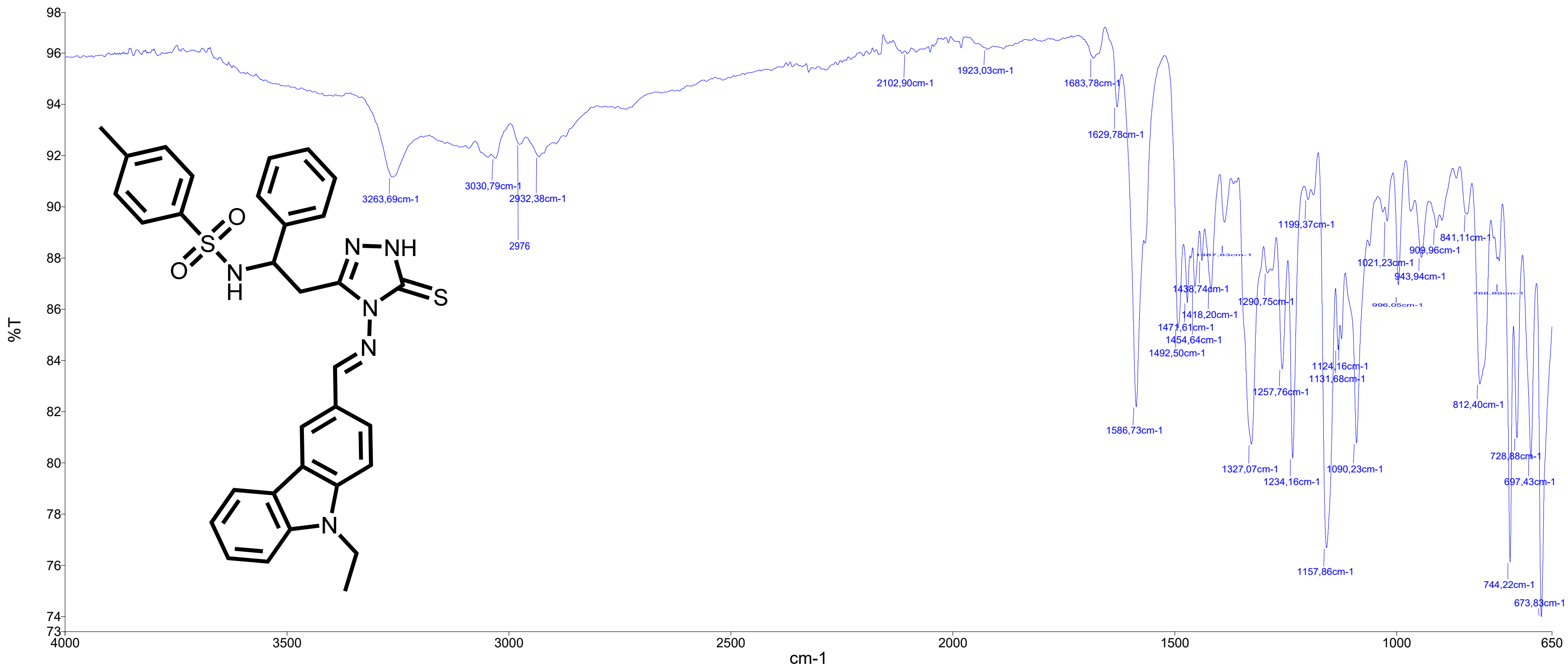

S63. FTIR spectra of (*E*)-*N*-[2-{4-[[(9-ethyl-9*H*-carbazol-3-yl)methylene]amino]-5-thioxo-4,5-dihydro-1*H*-1,2,4-triazol-3-yl}-1-phenylethyl]-4-methylbenzenesulfonamide (**13g**)

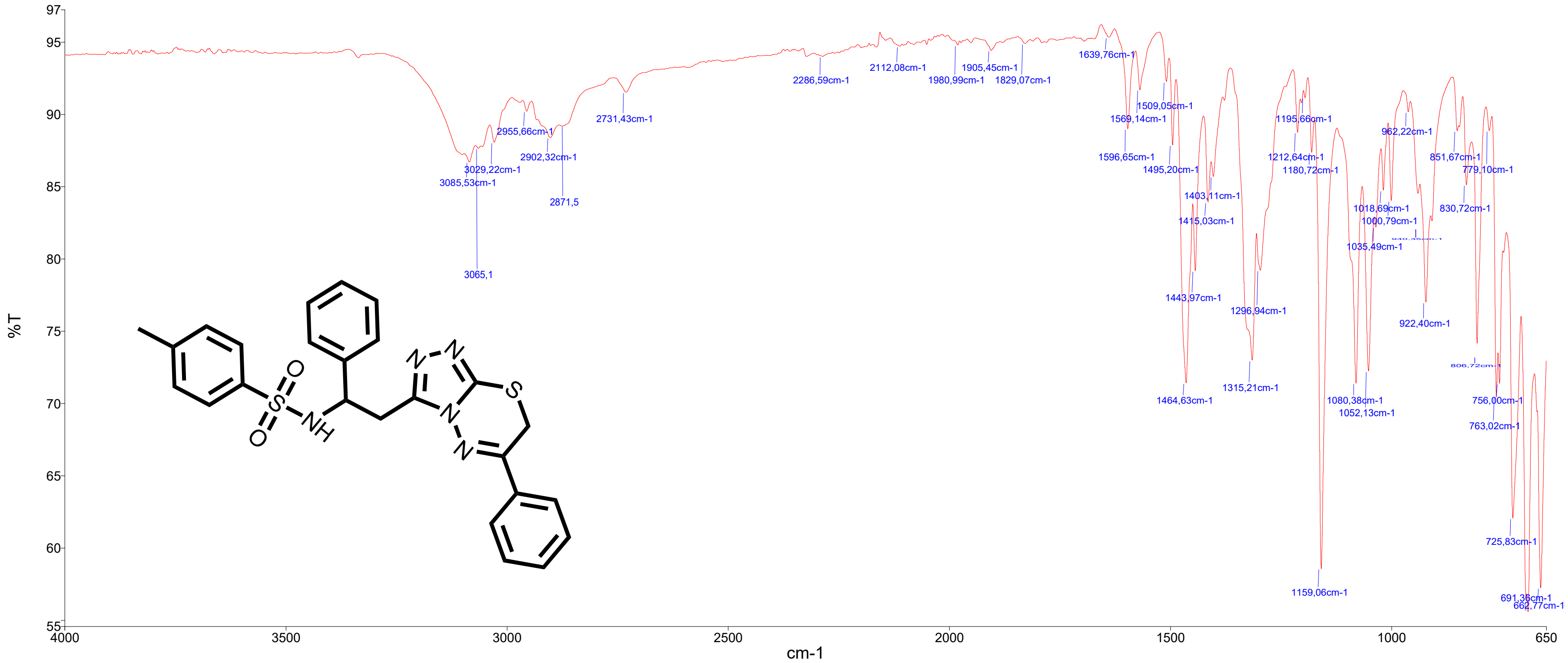

S64. FTIR spectra of 4-methyl-*N*-[1-phenyl-2-(6-phenyl-7*H*-[1,2,4]triazolo[3,4-*b*][1,3,4]thiadiazin-3-yl)ethyl]benzenesulfonamide (**14a**)

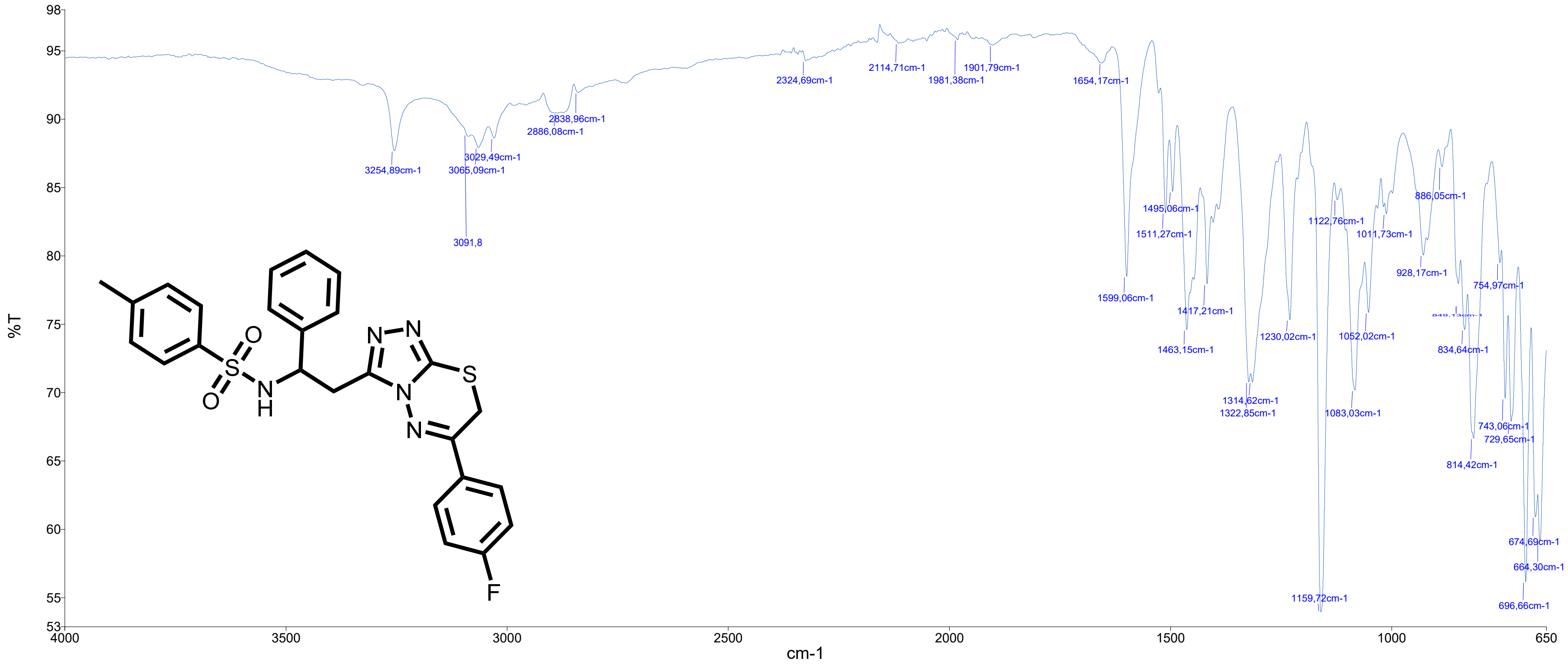

S65. FTIR spectra of *N*-{2-[6-(4-fluorophenyl)-7*H*-[1,2,4]triazolo[3,4-*b*][1,3,4]thiadiazin-3-yl]-1-phenylethyl}-4-methylbenzenesulfonamide (**14b**)

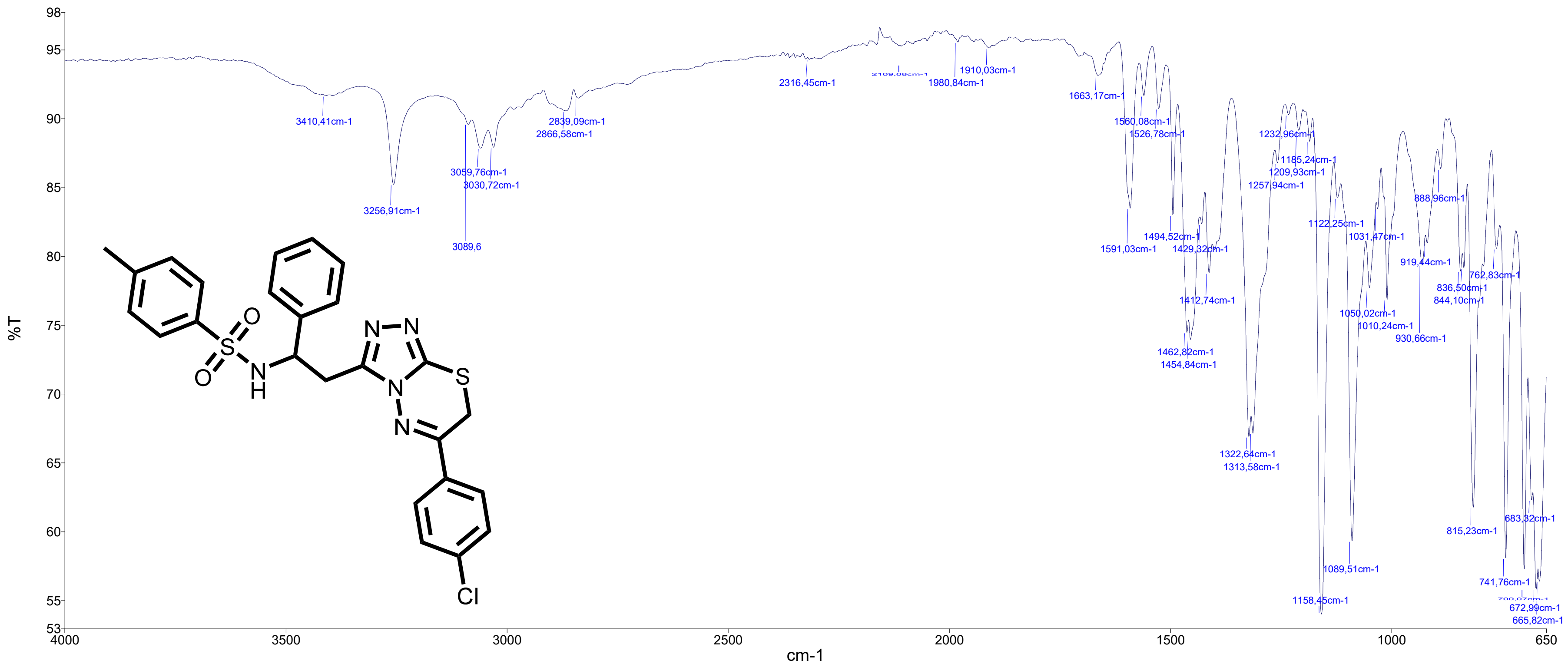

S66. FTIR spectra of *N*-{2-[6-(4-chlorophenyl)-7*H*-[1,2,4]triazolo[3,4-*b*][1,3,4]thiadiazin-3-yl]-1-phenylethyl}-4-methylbenzenesulfonamide (**14c**)

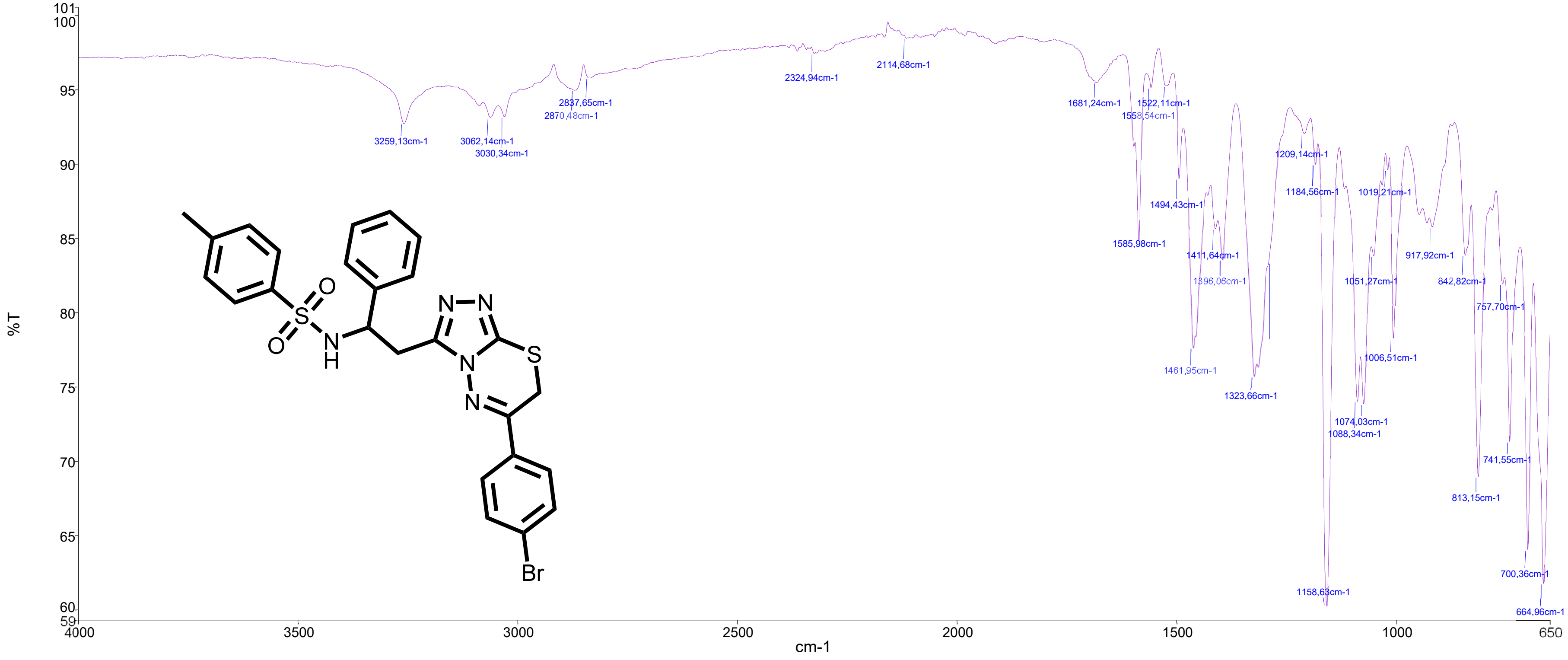

S67. FTIR spectra of *N*-{2-[6-(4-bromophenyl)-7*H*-[1,2,4]triazolo[3,4-*b*][1,3,4]thiadiazin-3-yl]-1-phenylethyl}-4-methylbenzenesulfonamide (**14d**)

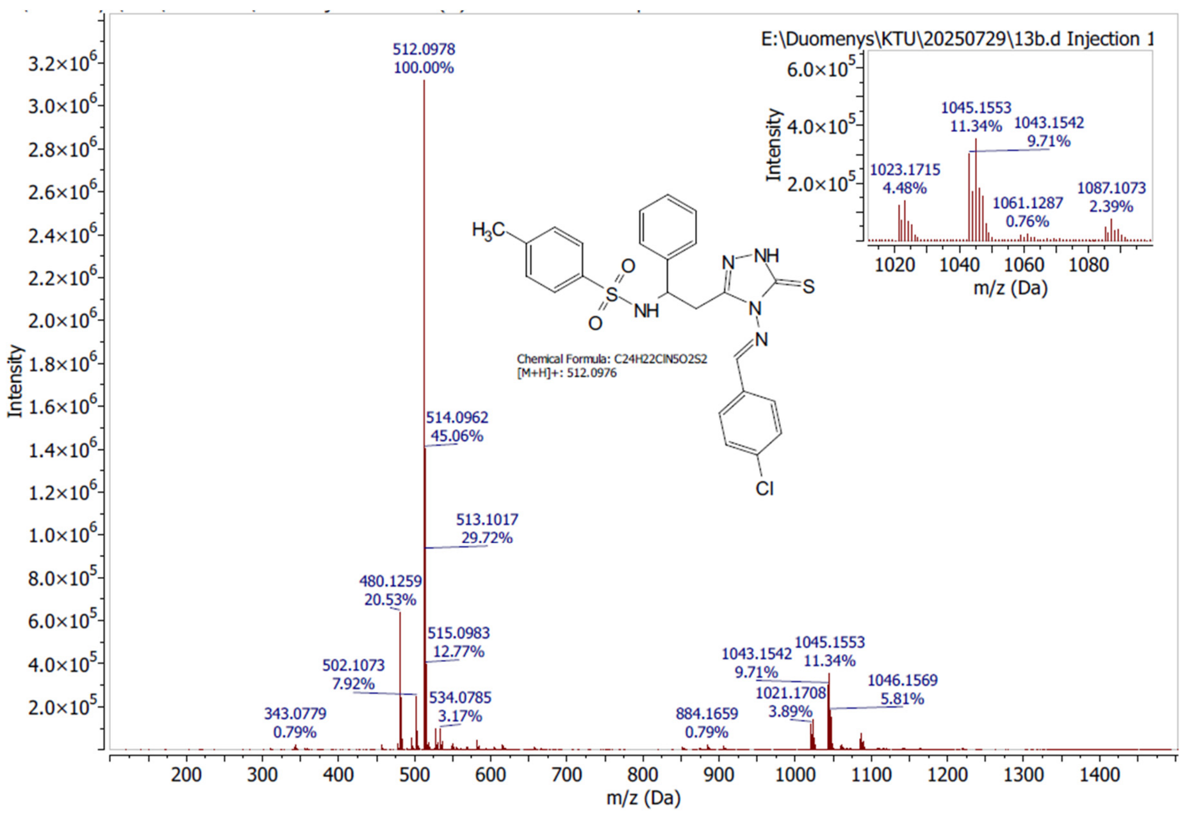

S68. HRMS spectra of (*E*)-*N*-{2-[4-[(4-chlorobenzylidene)amino]-5-thioxo-4,5-dihydro-1*H*-1,2,4-triazol-3-yl]-1-phenylethyl}-4-methylbenzenesulfonamide (**13b**)

Reference

1. Babij, N.R.; Mccusker, E.O.; Whiteker, G.T.; Canturk, B.; Choy, N.; Creemer, L.C.; De Amicis, C. V; Hewlett, N.M.; Johnson, P.L.; Knobelsdorf, J.A.; et al. NMR Chemical Shifts of Trace Impurities: Industrially Preferred Solvents Used in Process and Green Chemistry. **2016**, doi:10.1021/acs.oprd.5b00417.

2. Fulmer, G.R.; Miller, A.J.M.; Sherden, N.H.; Gottlieb, H.E.; Nudelman, A.; Stoltz, B.M.; Bercaw, J.E.; Goldberg, K.I. NMR Chemical Shifts of Trace Impurities: Common Laboratory Solvents, Organics, and Gases in Deuterated Solvents Relevant to the Organometallic Chemist. *Organometallics* **2010**, *29*, 2176–2179, doi:10.1021/OM100106E/SUPPL_FILE/OM100106E_SI_001.PDF.
